# Severity of the Omicron SARS‐CoV‐2 variant compared with the previous lineages: A systematic review

**DOI:** 10.1111/jcmm.17747

**Published:** 2023-05-18

**Authors:** Maryam Arabi, Yousef Al‐Najjar, Nada Mhaimeed, Mohammad A. Salameh, Pradipta Paul, Jamal AlAnni, Ali A. Abdelati, Ibrahim Laswi, Bushra Khanjar, Dana Al‐Ali, Abdallah Elshafeey, Omar Mhaimeed, Zain Burney, Ashton D'Souza, Pratyaksha Sinha, Mohammad Bhatti, Krishnadev V. Pillai, Moayad Homssi, Khalifa Bshesh, Lina Yagan, Dalia Zakaria

**Affiliations:** ^1^ Medical Division Weill Cornell Medicine‐Qatar Doha Qatar; ^2^ Department of Obstetrics and Gynecology Mayo Clinic Rochester Minnesota USA; ^3^ Cincinnati Children's Hospital Medical Center (CCHMC) Cincinnati Ohio USA; ^4^ Department of Dermatology Hamad Medical Corporation Doha Qatar; ^5^ Department of Pediatrics Hamad Medical Corporation Doha Qatar; ^6^ Department of Medicine The Johns Hopkins Hospital Baltimore Maryland USA; ^7^ Medicine Institution Cleveland Clinic Cleveland Ohio USA; ^8^ Department of Medicine University of Pennsylvania Hospital Philadelphia Pennsylvania USA; ^9^ Premedical Department Weill Cornell Medicine‐Qatar Doha Qatar

**Keywords:** Alpha, Beta, COVID‐19, death, Delta, hospitalization, ICU, Omicron, SARS‐CoV‐2, severity

## Abstract

The Omicron variant was first detected in October 2021, which evolved from the original SARS‐CoV‐2 strain and was found to possess many mutations. Immune evasion was one of the notable consequences of these mutations. Despite Omicron exhibiting increased transmissibility, the rates of hospitalizations and deaths among patients infected with this variant were substantially lower when compared to other strains. However, concluding that the Omicron variant is less severe than other variants of SARS‐CoV‐2 requires consideration of multiple factors, including the vaccination status of infected patients as well as any previous infections with other variants. This review compiled data about any reported indicators of severity in Omicron‐infected patients, including studies comparing Omicron with other variants while adjusting for confounders. A comprehensive search was conducted using different databases to target any studies about Omicron. In total, 62 studies met our inclusion criteria and were included in this study. Many studies reported a significantly reduced risk of hospitalization, ICU admission, need for oxygenation/ventilation, and death in Omicron‐infected patients compared to patients infected with other variants, such as Delta. Some studies, however, reported comparable severity in Omicron infected patients as to other variants emphasizing a substantial risk for severe illness. Furthermore, the COVID‐19 vaccines were less effective against Omicron relative to previous lineages, except after receiving the booster dose. One study recommended vaccination during pregnancy, which may help prevent future cases of severe SARS‐CoV‐2 pneumonia in neonates and young infants due to the transfer of humoral response from the mother.

## 1 INTRODUCTION

SARS‐CoV‐2 is a virus that emerged in December of 2019 and spread rapidly across the world causing an ongoing global COVID‐19 pandemic. Since its emergence, there have been nearly 45 million confirmed cases and over 6 million deaths.^1^ As of December 2021, five variants of concern have been identified by the World Health Organization: Alpha (B.1.1.7), Beta (B.1.351), Gamma (P.1), Delta (B.1.617.2 and AY lineages) and Omicron (B.1.1.529, then reclassified into BA lineages, notably, BA.1 and BA.2). In comparing these variants, studies show that some variants, such as Alpha, are more transmissible while others, such as Delta, are more pathogenic. The Delta variant is one of special interest and concern as it might cause more severe illness.^2^ The Omicron variant was first detected in South Africa on 24 October, 2021,^3^ and the subsequent discoveries made regarding the variant have been very troublesome. Research shows that the Omicron variant has 97 mutations when compared to the original SARS‐CoV‐2 strain, while the Delta variant only has 45 mutations in comparison to the original strain.^3^ One of the most concerning consequences of these mutations is that they render the virus to evade the immune response and recognition by T lymphocytes.^3^ Despite Omicron exhibiting increased transmissibility, the rates of hospitalizations and deaths are substantially lower relative to other strains.^4^ However, in order to draw conclusions related to the decreased severity of Omicron infections, multiple confounding factors including vaccination status and previous COVID‐19 infections must be taken into consideration.

This review compiles data about any reported severe symptoms or outcomes due to COVID‐19 infections with the Omicron variant. Reviewing the types of reported symptoms/complications post‐Omicron infections may give insight into the severity of the Omicron variant. However, comparative studies analysing the severity of Omicron and other variants of SARS‐CoV‐2, especially when adjusting for confounders, will give better insight into the possible risks posed by Omicron infections and potential methods for protection and treatment.

## 2 METHODS

The preferred reporting items for systematic reviews and metanalysis (PRISMA) statement was used to develop the protocol of this systematic review.^5^

### 2.1 Information sources and search strategy

A comprehensive search was conducted to target any studies about the new variant of SARS‐CoV‐2 using the following two keywords: Omicron or B.1.1.529. The following databases were searched in March 2022: PubMed, Medline, Embase, Scopus, Web of Science, Science Direct, MedRxiv and Lens.org. All searches were limited by year to 2021 through 2022.

### 2.2 Eligibility criteria

We conducted a comprehensive literature search of medical studies that reported any clinical data related to COVID‐19 infections with the Omicron variant. No restrictions were made based on country, age or gender. Any articles that did not have primary data, such as review articles, were excluded from the study after removing the duplicates. Furthermore, studies that were not in English were excluded. During the full‐text screening, any studies that reported positive Omicron infection were included, whether the subjects were symptomatic or asymptomatic. Only studies that stratified the data based on the type of SARS‐CoV‐2 variant were included.

### 2.3 Study selection and data collection

Title and abstract screening, full‐text screening and data extraction were conducted by two independent reviewers for each study using Covidence and disagreements were resolved by consensus.

### 2.4 Data items

Demographic and clinical data, including age, sex, comorbidities, treatments and outcomes, were collected. Continuous variables were expressed as mean ± standard deviation or range of results. Categorical variables were expressed as percentages.

### 2.5 Data analysis

Indicators of severity were classified based on the following criteria: Hospitalization, intensive care unit (ICU) admission, the need for oxygenation or ventilation, cardiovascular and haematological complications, other complications and death. The other complications included any of the above categories that were reported by some studies without separating the data of each category or those that described the symptoms as severe without specifying the symptoms or type of intervention. Furthermore, emergency department (ED) visits, respiratory failure, pneumonia, new renal replacement therapy (NRRT), lung filtrates on chest X‐ray/CT scan (CXR/CT), the use of vasopressors or the loss/impairment of smell and/or taste, were reported in this review under the category of other complications.

## 3 RESULTS

**FIGURE 1:**
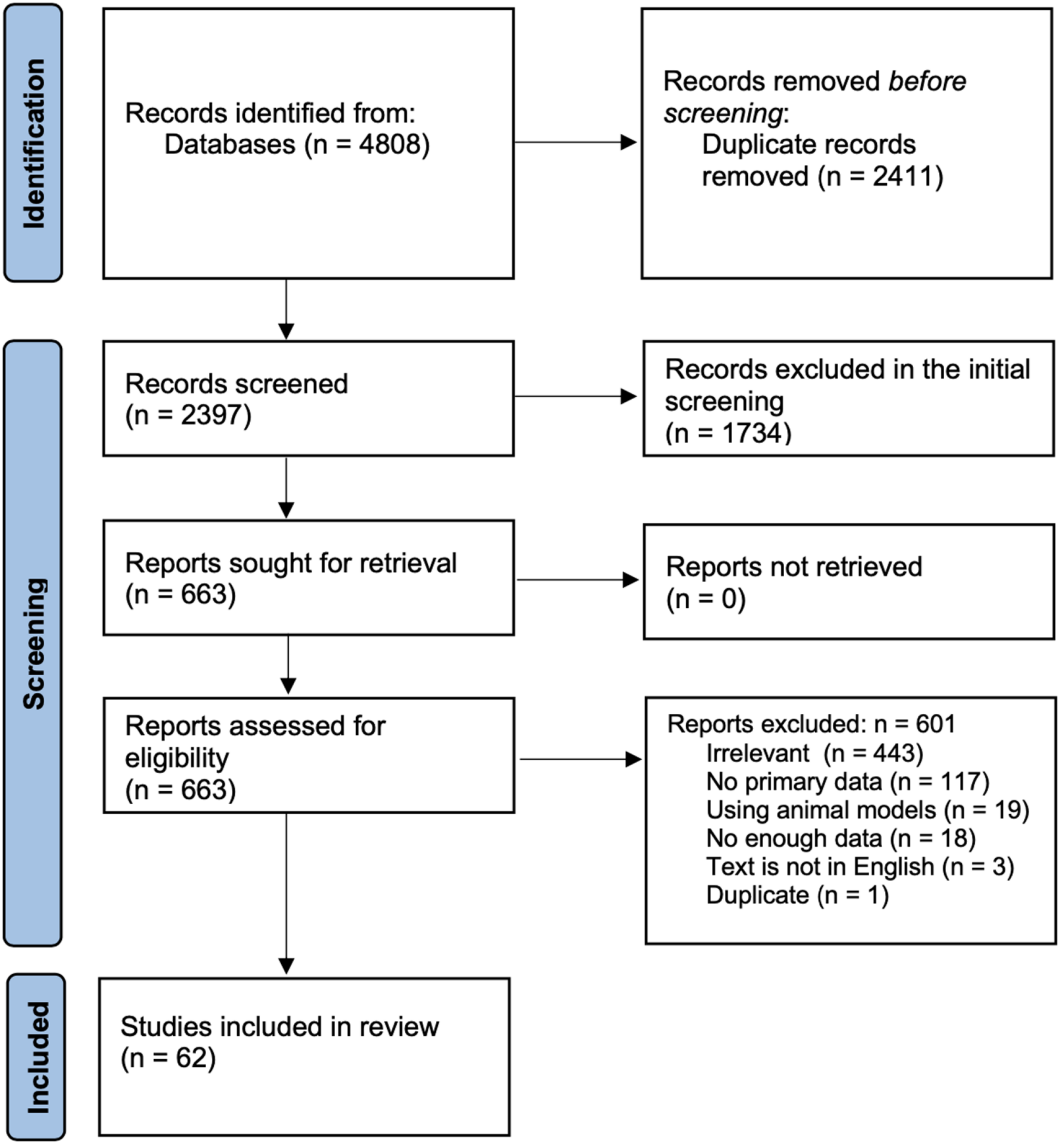
Screening and study selection protocol.

Figure 1 shows the flow diagram of our protocol. After removing the duplicates, the titles and abstracts of 2397 studies were screened, of which 663 were selected for full‐text screening. Only 62 studies met our inclusion criteria. Of the 601 excluded studies, 443 were irrelevant, 18 did not have enough data, 117 had no primary data, 3 were not in English, 19 used animal models, and 1 was a duplicate of another study. Tables S1 and S2 summarize the demographic and clinical data of the included subjects who had reported vaccination statuses (regardless of the dose).^5^, ^6^, ^7^, ^8^, ^9^, ^10^, ^11^, ^12^, ^13^, ^14^, ^15^, ^16^, ^17^, ^18^, ^19^, ^20^, ^21^, ^22^, ^23^, ^24^, ^25^, ^26^, ^27^, ^28^, ^29^, ^30^, ^31^, ^32^, ^33^ Table S3 summarizes the demographic and clinical data of the subjects in studies that did not report vaccination statuses.^34^, ^35^, ^36^, ^37^, ^38^, ^39^, ^40^, ^41^, ^42^, ^43^, ^44^, ^45^, ^46^, ^47^, ^48^, ^49^, ^50^, ^51^, ^52^, ^53^, ^54^, ^55^, ^56^, ^57^, ^58^, ^59^, ^60^, ^61^, ^62^, ^63^, ^64^, ^65^, ^66^, ^67^

### 3.1 Types of studies and demographic data

Among the 62 included studies, 23 were from the US, 11 from South Africa, 5 from France, 3 from Italy, 2 from South Korea, 3 from the UK, 2 from Norway, 3 from Denmark and 1 each from Canada, Qatar, Jordan, India, Sweden, Turkey, Spain, Japan, Israel, Brazil and Portugal. The studies included 46 cohort studies, 6 case‐control studies, 6 case series, 2 case reports and 2 surveys, which reported a total of 8,388,855 subjects infected with SARS‐CoV‐2. These studies collectively reported 2,746,072 subjects infected with Omicron, 3,787,701 infected with Delta, 101,839 infected with Alpha, 918,101 infected with Beta, 246,608 infected with the Ancestral strain, and 587,901 infected with other variants of SARS‐CoV‐2. However, we have not compiled the number of subjects across the included studies who had certain indicators of severity due to the possible overlap between some of the studies, which used database registries as the source of their reported data. We, therefore, reported the data as rates of incidence of certain severe symptoms, outcomes or interventions among each cohort.

### 3.2 Clinical data

Tables S2 and S3 summarize the clinical data of the subjects that took part in each study, including comorbidities and any reported clinical manifestations associated with Omicron infections such as symptomatic and asymptomatic infections. We also noted any clinical data related to COVID‐19 infections with other variants whenever reported. Micheli et al.,^19^ Piersiala et al.,^23^ and Accorsi et al. reported asymptomatic or non‐severe symptoms of Omicron infection.^64^

**FIGURE 2:**
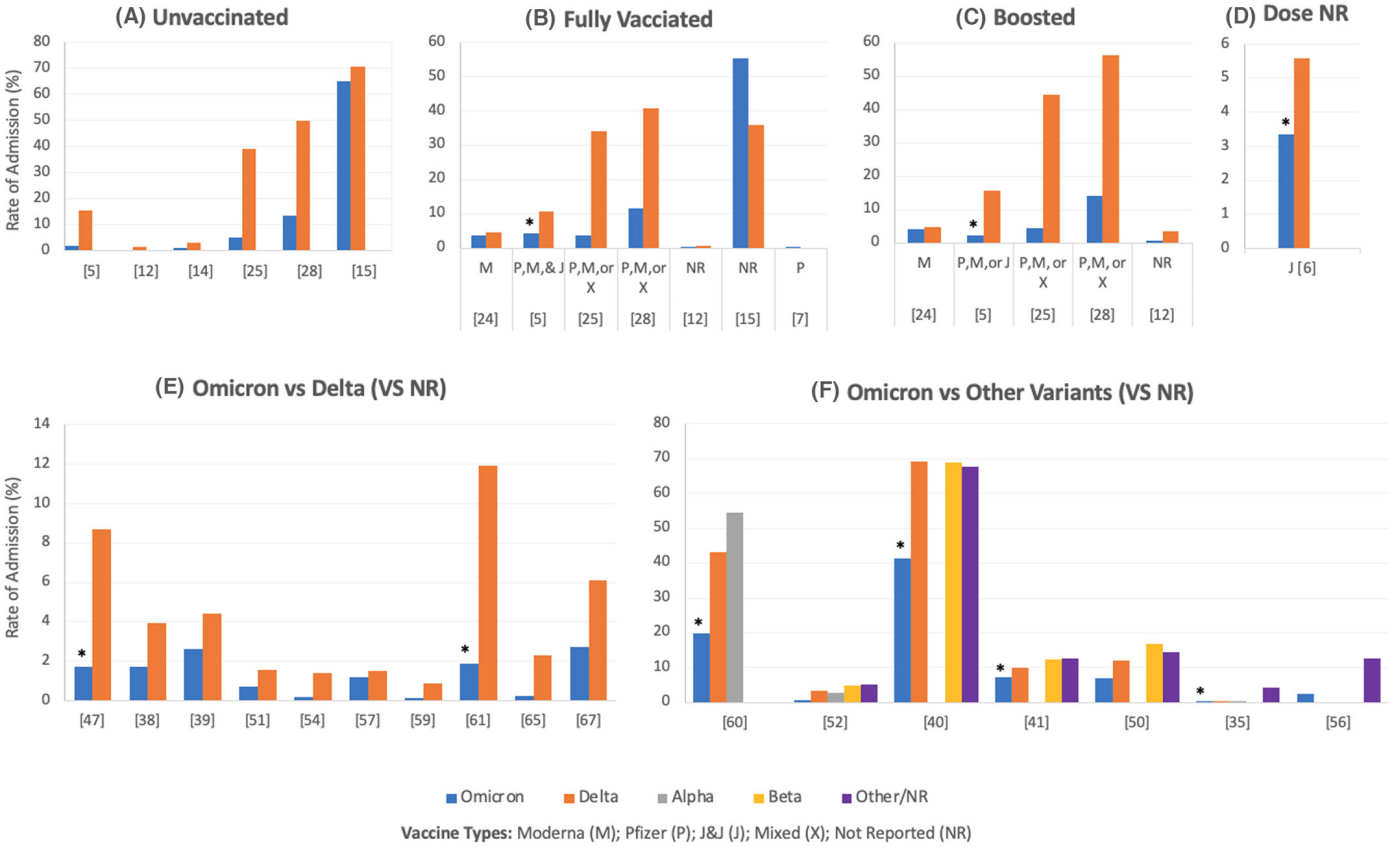
Rates of hospital admissions among patients infected with Omicron compared to other variants of SARS‐CoV‐2. (A–F) Illustrate the rates in unvaccinated patients (A), fully vaccinated patients (B), vaccine‐boosted patients (C), mixed data for partially and fully vaccinated patients (D) and those who did not have known/reported vaccination status (E for Omicron vs. Delta, F for Omicron vs. multiple variants). All the studies showed lower admission rates among Omicron‐infected patients compared to the other variants, regardless of the vaccination status or doses. Some studies reported the significance of the difference between the Omicron variant and the other variants. Specifically, *p* < 0.0001 and 0.00007 were reported for fully vaccinated (B) and boosted patients (C), respectively (Fall et al.^5^). Goga et al.^6^ reported significantly lower admission rates among the J&J fully vaccinated and boosted patients (D) infected with Omicron compared to those infected with Delta (*p* < 0.001). Krutikov et al.,^47^ Houhamdi et al.^61^ (E) and Maslo et al.^40^ (F). reported significantly lower rates of hospitalization among Omicron‐infected patients with unknown/unreported vaccination status compared to Delta patients (*p* < 0.0001, *p* < 0.0001 and *p* < 0.001, respectively). When Omicron was compared with other variants (including Delta, F), the rates of hospital admissions were also reported to be significantly lower among Omicron‐infected patients by Christensen et al.^60^ (*p* < 0.0001 for Omicron and either Delta or Alpha), Jassat et al.^41^ (*p* < 0.001 for Omicron and either Delta or Beta or D614G) and Dinh et al.^35^ (*p* < 0.0001 for Omicron and Alpha). In Figure F, no values are shown for some variants because the variants were not reported by these studies, not because the admission rate was 0% as illustrated in Table S4. *Rate is significantly lower for Omicron as compared to other variants (specified in the legend).

**FIGURE 3:**
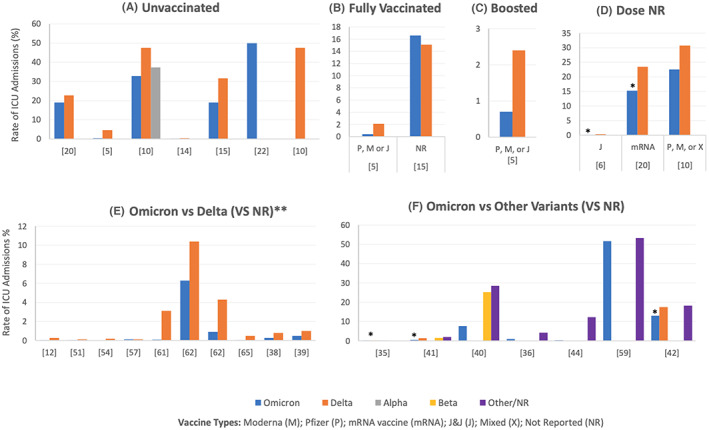
ICU admissions among patients infected with Omicron compared to other variants of SARS‐CoV‐2. (A–F) Illustrate the rates in unvaccinated patients (A), fully vaccinated patients (B), vaccine‐boosted patients (C), mixed data for partially and fully vaccinated patients (D) and those who did not have known/reported vaccination status (E for Omicron vs. Delta, F for Omicron vs. multiple variants). All the studies showed lower admission rates among Omicron‐infected patients compared to other variants regardless of the vaccination status or the number of doses, except for Marks et al.,^15^ who reported a higher admission rate among fully vaccinated Omicron‐infected patients without reporting the significance of the difference. Most studies did not report the statistical significance of the difference between different variants. However, Modes et al.^20^ and Fall et al.^5^ reported that the differences were not significant for unvaccinated patients (A). Similarly, there was no significant difference between the ICU admissions in boosted Omicron and Delta‐infected patients (C, Fall et al.^5^). Significantly lower admission rates were reported among fully vaccinated and boosted Omicron‐infected patients compared with Delta‐infected patients (D, Goga et al.,^6^ *p* < 0.001; Modes et al.,^20^ *p* = 0.01). Furthermore, among the studies that did not report the vaccination status of the included subjects, significantly lower ICU admission rates were reported by Houhamdi et al.^61^ (E, *p* < 0.0001 for Omicron and Delta), Dinh et al.^35^ (F, *p* < 0.0001 for Omicron and Alpha), Iuliano et al.^42^ (*p* < 0.05 for Omicron and Delta and Omicron and the variants of the winter period), Maslo et al.^42^ (F, *p* < 0.001 for Omicron and Delta) and Jassat et al.^41^ (F, *p* < 0.001 for Omicron and either Delta or Beta or D614G). In Figure F, some values are not shown for some variants (except Omicron) because the variants were not reported by these studies, and not because the admission rate was 0%. However, sometimes no values are shown for Omicron because either the value is very low or 0% as illustrated in Table S5. *Rate is significantly lower for Omicron as compared to other variants (specified in the legend). **Vieillard‐Baron et al.^13^ reported 67.2% and 94.8% ICU admissions for Omicron and Delta, respectively. Data are not shown in the figure since the percentages are too high for the scale. ICU, intensive care unit.

**FIGURE 4:**
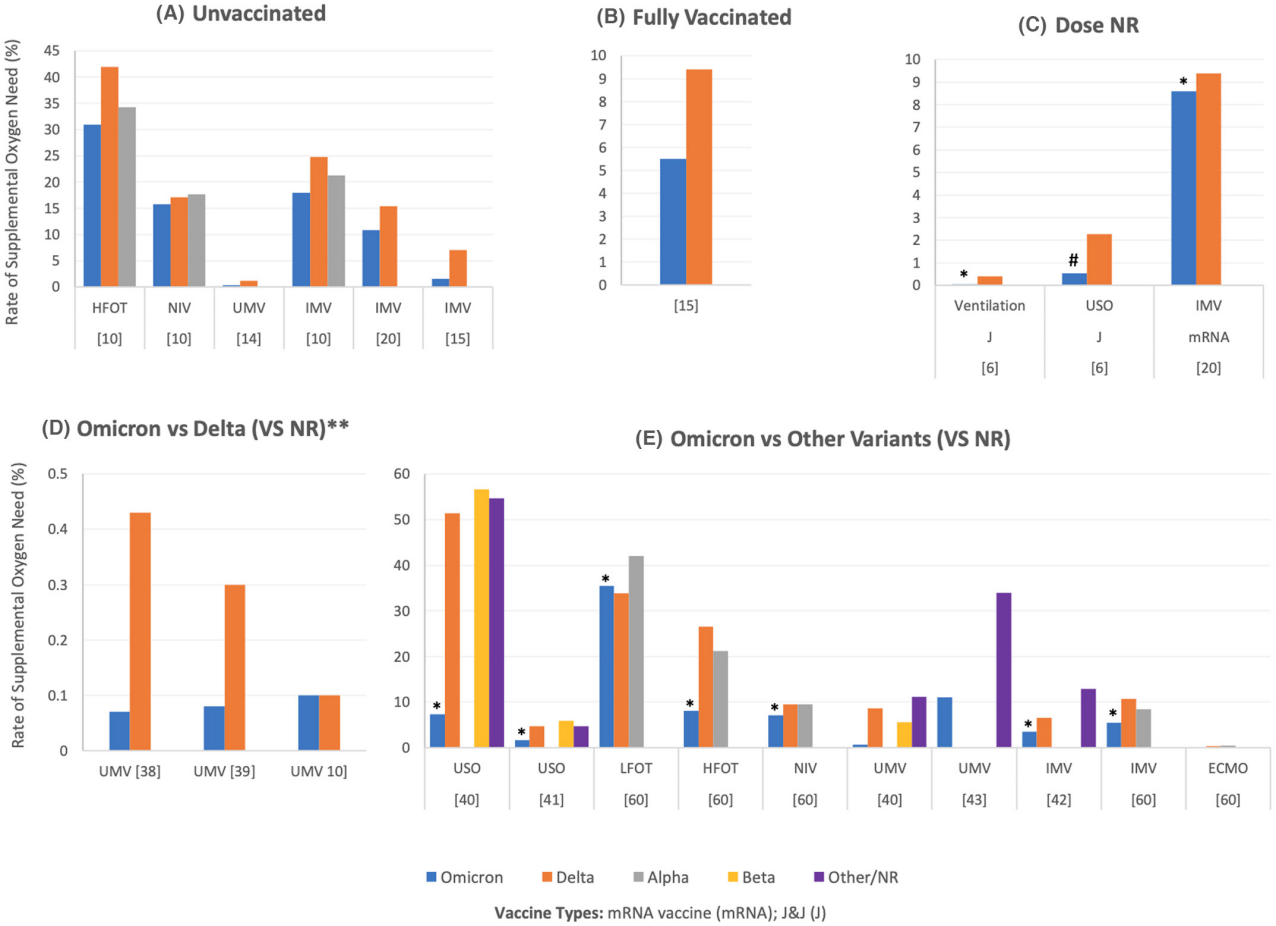
Rates of the need for oxygenation/ventilation among patients infected with Omicron compared to other variants of SARS‐CoV‐2. (A–E) illustrate the rates in (A) unvaccinated patients, (B) fully vaccinated patients, (C) vaccine‐boosted patients, (D) mixed data for partially and fully vaccinated patients, and (E) those who did not have known/reported vaccination status. All the studies showed lower rates among Omicron patients compared to other variants regardless of the vaccination status or the number of doses, except for Goga et al.^3^ (C) who reported a significantly higher rate of need for oxygenation among vaccinated Omicron‐infected patients (dose NR) as compared to Beta and Delta and Lewnard et al., who reported similar rates of UMV in the Omicron and Delta infected patients without reporting their vaccination status. While some studies did not report the statistical significance of the difference between the different variants, Modes et al.^20^ showed that the difference in the rate of IMV was not significant between the unvaccinated Omicron and Delta patients. A significantly lower rate of ventilation was reported by Goga et al.^3^ (C) among fully vaccinated or boosted Omicron patients compared to the Delta variant (*p* < 0.001), while the rate of need for oxygenation was significantly higher among Omicron patients relative to Delta patients (Goga et al.^3^ *p* < 0.001). The need for IMV was significantly lower among Omicron patients compared to Delta patients (C, Modes et al.,^20^ *p* = 0.03). Significantly lower rates were reported by Maslo et al.^40^ (E, *p* < 0.001 for USO and UMV in Omicron and Delta patients). Jassat et al.^41^ (E, *p* < 0.001 for USO in Omicron and D614G) and Christensen et al.^60^ (E, *p* < 0.0001 for LFOT, HFOT, NIV, IMV and ECMO in Omicron vs. either Delta or Alpha variants). In Figure E, values are not shown for some variants (except Omicron) because the variants were not reported by the corresponding studies, and not because the rate was 0%. However, sometimes no values are shown for Omicron because the value is very low as illustrated in Table S6. *Rate is significantly lower for Omicron as compared to other variants (specified in the legend). ^#^Rate is significantly higher for Omicron. **Vieillard‐Baron et al.^13^ reported 67.2% and 94.8% ICU admissions for Omicron and Delta, respectively. Data are not shown in the figure since the percentages are too high for the scale. ECMO, extracorporeal membrane oxygenation; HFOT, high flow oxygen therapy; IMV, invasive mechanical ventilation; LFOT, low flow oxygen therapy; NIV, non‐invasive ventilation; NOS, nasal oxygen support; NPO, nasal prong oxygen; NR, not reported; UMV, unspecified mechanical ventilation; USO, unspecified supplementary oxygen.

**FIGURE 5:**
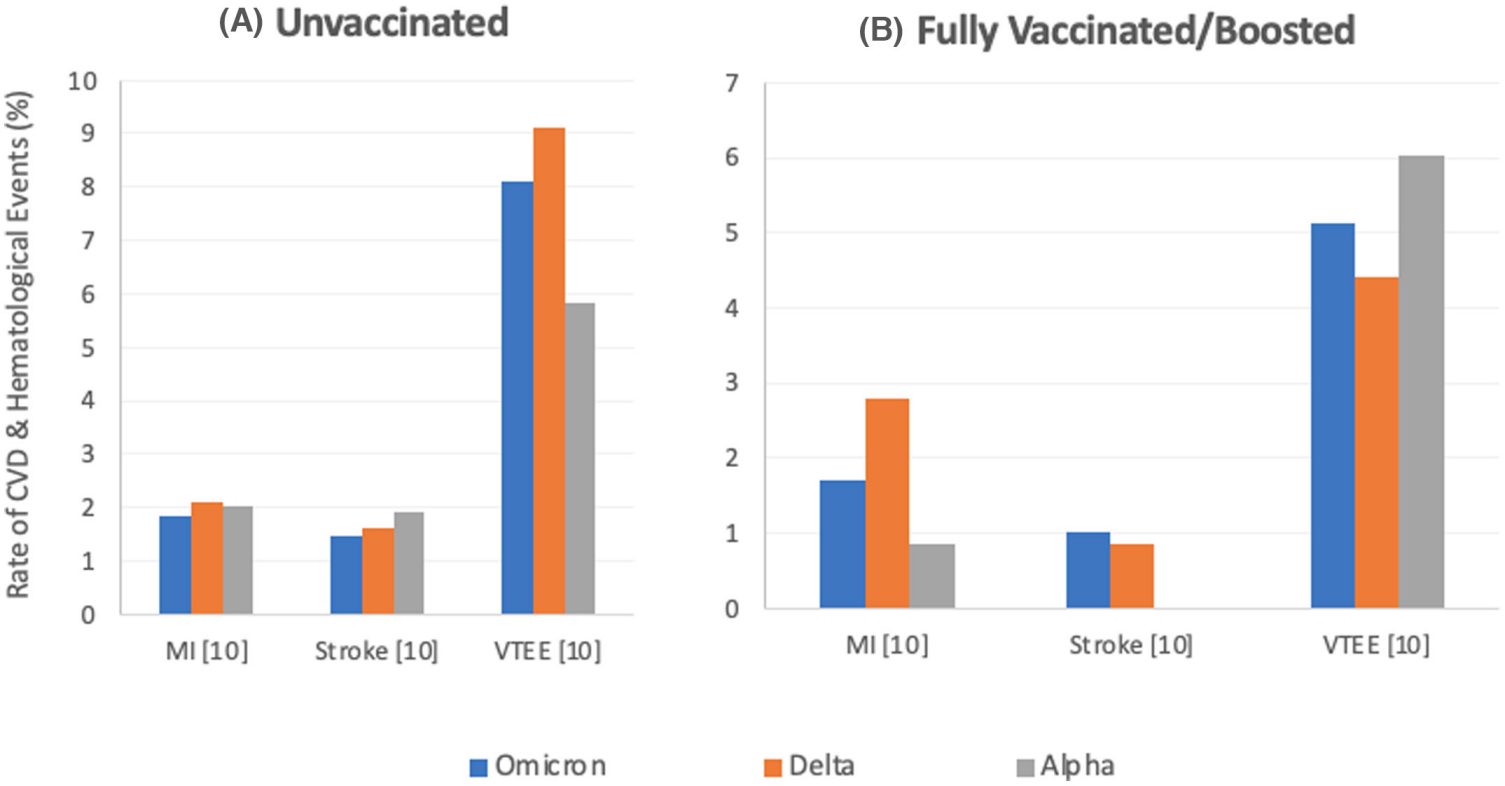
Rates of incidence of cardiovascular and/or haematological events among patients infected with Omicron compared to other variants of SARS‐CoV‐2 with different vaccination statuses as reported by the included studies. (A) Rates among unvaccinated patients infected with Omicron, Alpha, or Delta as reported by one study (Lauring et al.^10^), which showed lower rates of MI and stroke in Omicron‐infected patients compared to both Alpha and Delta. Lower rates of VTEE in Alpha‐infected patients were reported compared with both Omicron and Delta. However, the statistical significance of the differences was not reported. (B) Rates among fully vaccinated and boosted patients infected with Omicron, Alpha, or Delta as reported by Lauring et al.,^10^ which showed lower rates of MI in Omicron‐infected patients compared with both Alpha and Delta‐infected patients. Higher rates of strokes and VTE were reported among Omicron‐infected patients compared to Delta‐infected patients. However, the statistical significance of the differences was not reported. More details are reported in Table S7. CVST, cerebral venous sinus thrombosis; MI, myocardial infarction; NR, not reported; VTEE, venous thromboembolic events.

**FIGURE 6:**
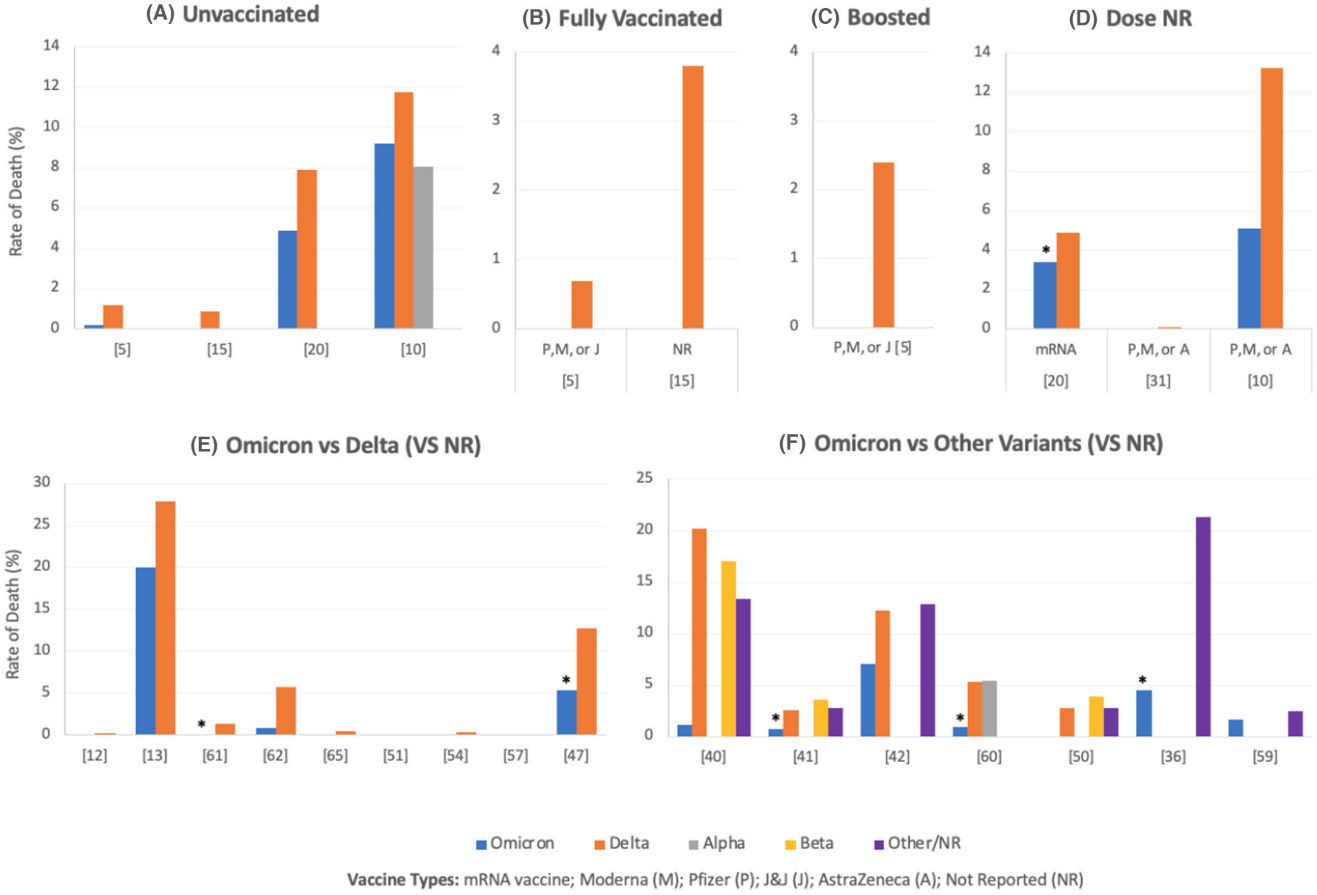
Death rates among patients infected with Omicron compared to other variants of SARS‐CoV‐2. (A–F) Illustrate the rates in unvaccinated patients, fully vaccinated patients (B), vaccine‐boosted patients (C), mixed data for partially and fully vaccinated patients (D) and those who did not have known/reported vaccination status (E for Omicron vs. Delta, f for Omicron vs. multiple variants). All the studies showed lower rates among Omicron‐infected patients compared to other variants, regardless of the vaccination status or the number of doses, except for Lauring et al.^10^ (A) who reported a higher death rate among unvaccinated Omicron‐infected patients compared to Alpha without reporting the statistical significance of the difference. Modes et al.^20^ (D) reported no significant difference in the death rates in the unvaccinated patients of Omicron and Delta; however, the rate was significantly lower in the fully vaccinated Omicron‐infected patients (*p* = 0.02). Furthermore, the death rate among Omicron‐infected patients (vaccination status NR) was reported to be significantly lower than other variants by Houhamdi et al.^61^ (E, *p* < 0.0001, for Omicron and Delta), Krutikov et al.^47^ (E, *p* < 0.0001, for Omicron and Delta), Jassat et al.^41^ (F, *p* < 0.001 for Omicron and either Delta, Beta or D614G), Christensen et al.^60^ (F, *p* < 0.0001 for Omicron and Delta, *p* < 0.0001 for Omicron and Alpha) and Abdullah et al.^36^ (F, *p* < 0.00001 for Omicron and Delta). In Figure F, values are not shown for some variants (except Omicron) because the variants were not reported by these studies, and not because the death rate was 0%. However, sometimes no values are shown for Omicron because the value is very low or 0% as illustrated in Table S8. *Rate is significantly lower for Omicron as compared to other variants (specified in the legend).

Tables [Link], [Link], [Link], [Link], [Link], [Link] summarize the rates of hospitalization, admission to the ICU, the need for oxygenation/ventilation, cardiovascular and haematological complications, death and other complications in COVID‐19 patients infected with different SARS‐CoV‐2 variants, respectively. The data has been classified based on the vaccination status, vaccine type and dose when specified. In addition, Figures 2, 3, 4, 5, 6 illustrate the rates of hospitalization, admission to the ICU, the need for oxygenation/ventilation, cardiovascular and haematological complications and death in Omicron infected patients compared to the other variants. Therefore, the figures present only the data reported by the studies that compared Omicron with other variants. Any data reported specifically for Omicron is found in Tables [Link], [Link], [Link], [Link], [Link], [Link], [Link], [Link], [Link].

As not all studies reported the statistical significance of differences between the cohorts, Table 1 summarizes the studies that tested the statistical significance of the differences between the cohorts infected with Omicron compared with other variants, whether the results were significant or not.

**TABLE 1:** Studies that tested the significance of the severity difference between Omicron and the other variants.

Study	Vaccine	Type of event	Omicron				Other variants					*p*‐value
			UV	FV	Boosted	VS NR	Variant	UV	FV	Boosted	VS NR	
Fall et al.^5^	Pfizer, Moderna, or J&J	Hospitalization	1.8	4.3	2.2	–	Delta	15.4	10.6	15.7	–	*p* < 0.0001 (Omicron/Delta) *p* = 0.00007 (Omicron/Delta boosted)
Goga et al.^6^	J&J	Hospitalization	–	3.34 (1–2D)		–	Delta	–	5.59 (1–2D)		–	*p* < 0.001 (Omicron/Delta)
Dinh et al.^35^	NR	Hospitalization	–	–	–	0.06	Alpha	–	–	–	0.19	*p* < 0.0001 (Omicron/Alpha)
Maslo et al.^40^	Pfizer or J&J	Hospitalization	–	–	–	–	Delta	–	–	–	69.3	*p* < 0.001 (Omicron/Delta)
Jassat et al.^41^	Pfizer or J&J	Hospitalization	–	–	–	7.29	D614G	–	–	–	12.72	*p* < 0.001 (Omicron/D614G)
	Pfizer or J&J	Hospitalization	–	–	–		Beta	–	–	–	12.43	*p* < 0.001 (Omicron/Beta)
	Pfizer or J&J	Hospitalization	–	–	–	–	Delta	–	–	–	9.84	*p* < 0.001 (Omicron/Delta)
Krutikov et al.^47^	Pfizer, AstraZeneca, or NR	Hospitalization	–	–	–	4.5	Delta	–	–	–	10.5	*p* < 0.0001 (Omicron/Delta)
Christensen et al.^60^	Pfizer, Moderna, or J&J	Hospitalization	–	–	–	19.8	Alpha	–	–	–	54.6	*p* < 0.0001 (Omicron /Alpha)
	Pfizer, Moderna, or J&J	Hospitalization	–	–	–	–	Delta	–	–	–	43.1	*p* < 0.0001 (Omicron /Delta)
Houhamdi et al.^61^	NR	Hospitalization	–	–	–	1.9	Delta	–	–	–	11.9	*p* < 0.0001 (Omicron /Delta)
Wang et al.^14^	NA	Hospitalization	1.04	–	–	–	Delta	3.14	–	–	–	RR 0.33 (0.26–0.4)
Wang et al.^38^	Pfizer, Moderna or J&J	Hospitalization	–	–	–	1.75	Delta	–	–	–	3.95	RR 0.44 (0.38–0.52)
Wang et al.^39^	NR	Hospitalization	–	–	–	2.6	Delta	–	–	–	4.44	RR 0.58 (0.55–0.6)
Veneti et al.^12^	NR	Hospitalization	0.186	0.22	0.87	–	Delta	1.46	0.64	3.65	–	HR 0.13 (0.07–0.23) UV HR 0.19 (0.08–0.43) boosted
Lewnard et al.^51^	Pfizer, Moderna, or J&J	Hospitalization	–	–	–	0.73	Delta	–	–	–	1.57	aHR 0.59 (0.51–0.69) overall aHR 0.40 (0.33–0.49) UV aHR 0.99 (0.61–1.61) boosted (aHR also available for other vaccination statuses)
	Pfizer, Moderna, or J&J	Hospitalization	–	–	–	1.47 BA2	–	–	–	–	–	aHR 1.26 (0.83–1.92) overall BA.1 versus BA.2
	Pfizer, Moderna, or J&J	Hospitalization	–	–	–	1.33 BA1	–	–	–	–	–	–
Marks et al.^15^	NR	Hospitalization	65.0	55.5	–	–	Delta	70.8	35.8	–	–	RR 5.4 (4.0–7.2) 0–4 years overall RR 2.3 (1.5–3.6) 5–11 years overall RR 3.5 (2.5–5.0) 12–17 years overall RR 6.3 (4.4–8.6) 12–17 years UV versus V
Paredes et al.^52^	Pfizer, Moderna, or J&J	Hospitalization	–	–	–	0.7	Ancestral	–	–	–	2.3	HR 0.92 (0.56–1.52) overall HR 0.79 (0.37–1.67) UV HR 0.49 (0.29–0.83) 1–2 doses HR 0.44 (0.21–0.93) boosted
	Pfizer, Moderna, or J&J	Hospitalization	–	–	–	–	Alpha	–	–	–	2.7	–
	Pfizer, Moderna, or J&J	Hospitalization	–	–	–	–	Beta	–	–	–	4.8	–
	Pfizer, Moderna, or J&J	Hospitalization	–	–	–	–	Gamma	–	–	–	5.3	=
	Pfizer, Moderna, or J&J	Hospitalization	–	–	–	–	Delta	–	–	–	3.3	HR 0.34 (0.23–0.50) overall HR 0.37 (0.21–0.66) UV HR 0.23 (0.14–0.39) 1–2 doses HR 0.19 (0.09–0.41) boosted
	Pfizer, Moderna, or J&J	Hospitalization	–	–	–	–	Epsilon	–	–	–	2.1	–
	Pfizer, Moderna, or J&J	Hospitalization	–	–	–	–	Iota	–	–	–	2.0	–
Peralta‐ Santos et al.^54^	Pfizer, Moderna, Astra Zeneca, or J&J	Hospitalization	–	–	–	0.2	Delta	–	–	–	1.39	*p* < 0.001 (Omicron/Delta) aHR 0.25 (0.15–0.43) overall Omicron had 75% risk reduction (57%–85%) compared to Delta aHR 0.21 (0.08–0.51) UV aHR 0.34 (0.13–0.87) V
Ulloa et al.^65^	NR	Hospitalization	–	–	–	0.24	Delta	–	–	–	2.28	–
Fall et al.^5^	Pfizer, Moderna, or J&J	ICU	0.4	0.4	0.7	–	Delta	4.6	2.1	2.4	–	*p* < 0.00001 (Omicron/Delta) *p* = 0.6 (Omicron/Delta boosted)
Goga et al.^6^	J&J	ICU	–	0.11 (1–2D)		–	Delta	–	0.37 (1–2D)		–	*p* < 0.001 (Omicron/Delta V)
Vieillard Baron et al.^13^	Pfizer, Moderna, or AstraZeneca	ICU	–	–	–	67.2	Delta	–	–	–	94.8	*p* < 0.001 (Omicron/Delta) hospitalized
Iuliano et al.^42^	NR	ICU	–	–	–	13.0	Delta	–	–	–	17.5	*p* < 0.05 (Omicron/Delta) hospitalized
	NR	ICU	–	–	–	–	Winter‐ period	–	–	–	18.2	*p* < 0.05 (Omicron/Winter‐ period) hospitalized
Modes et al.^20^	mRNA vaccine	ICU	19.0	–	–	–	Delta	22.8	23.5 (2–3D)		–	*p* = 0.10 (Omicron/Delta V) *p* = 0.27 (Omicron/Delta UV) *p* = 0.01 (Omicron/Delta) hospitalized
Dinh et al.^35^	NR	ICU	–	–	–	–	Alpha	–	–	–	0.10	*p* < 0.0001 (Omicron /Alpha)
Abdullah et al.^36^	NR	ICU	–	–	–	1.0	Ancestral, Beta, Delta	–	–	–	4.3	*p* = 0.0007 (Omicron/Delta) hospitalized
Maslo et al.^40^	NR	ICU	–	–	–	–	Delta	–	–	–	20.78	*p* < 0.001 (Omicron/Delta)
Jassat et al.^41^	NR	ICU	–	–	–	0.46	D614G	–	–	–	2.01	*p* < 0.001 (Omicron /D614G)
	NR	ICU	–	–	–		Beta	–	–	–	1.60	*p* < 0.001 (Omicron /Beta)
	NR	ICU	–	–	–	–	Delta	–	–	–	1.44	*p* < 0.001 (Omicron /Delta)
AraujodaSilva et al.^48^	NR	ICU	–	–	–	51.6	Pre‐Omicron	–	–	–	53.3	*p* = 0.894 (Omicron/pre‐Omicron) hospitalized children
Houhamdi et al.^61^	NR	ICU	–	–	–	0.1	Delta	–	–	–	3.1	*p* < 0.0001 (Omicron /Delta)
Wang et al.^14^	NA	ICU	0.14	–	–	–	Delta	0.43	–	–	–	RR 0.32 (0.16–0.66)
Wang et al.^38^	Pfizer, Moderna, or J&J	ICU	–	–	–	0.26	Delta	–	–	–	0.78	RR 0.33 (0.23–0.48)
Wang et al.^39^	NR	ICU	–	–	–	0.47	Delta	–	–	–	1	RR 0.47 (0.43–0.51)
Lewnard et al.^51^	Pfizer, Moderna, or J&J	ICU	–	–	–	0.03	Delta	–	–	–	0.12	aHR 0.50 (0.29–0.87) overall aHR 0.34 (0.17–0.66) UV aHR 0.95 (0.33–2.76) V
	Pfizer, Moderna, or J&J	ICU	–	–	–	0.05 BA2	–	–	–	–	–	–
	Pfizer, Moderna, or J&J	ICU	–	–	–	0.05 BA1	–	–	–	–	–	–
Peralta‐ Santos et al.^54^	Pfizer, Moderna, AstraZeneca, or J&J	ICU	–	–	–	0	Delta	–	–	–	0.18	*p* = 0.317 hospitalized
Ulloa et al.^65^	NR	ICU	–	–	–	0.02	Delta	–	–	–	0.47	–
Goga et al.^6^	J&J	Ventilation	–	0.05 (1–2D)			Delta	–	0.40 (1–2D)		–	*p* < 0.001 (Omicron/Delta V) hospitalized
	J&J	Oxygenation	–	0.52 (dose NR)			Delta	–	2.26 (1–2D)		–	*p* < 0.001 (Omicron/Delta V) hospitalized
Vieillard‐ Baron et al.^13^	Pfizer, Moderna, or AstraZeneca	IMV	–	–	–	41.0	Delta	–	–	–	51.0	*p* = 0.02 (Omicron /Delta) hospitalized
Iuliano et al.^42^	NR	IMV	–	–	–	3.5	Delta	–	–	–	6.6	*p* < 0.001 (Omicron/Delta) hospitalized
	NR	IMV	–	–	–	–	Winter 2020/2021	–	–	–	7.5	*p* < 0.001 (Omicron/Winter period) hospitalized
Modes et al.^20^	mRNA vaccine	IMV	10.8	8.6 (2–3D)		–	Delta	15.4	9.4 (2–3D)		–	*p* = 0.82 (Omicron/Delta V) *p* = 0.11 (Omicron/Delta UV) *p* = 0.03 (Omicron/Delta) hospitalized
Maslo et al.^40^	Pfizer or J&J	MV	–	–	–	–	Delta	–	–	–	8.64	*p* < 0.001 (Omicron/Delta)
	Pfizer or J&J	Oxygen therapy	–	–	–		Delta	–	–	–	51.4	*p* < 0.001 (Delta & Omicron)
Jassat et al.^41^	Pfizer or J&J	Supplemental oxygen	–	–	–	1.68	D614G	–	–	–	4.68	*p* < 0.001 (Omicron /D614G)
	Pfizer or J&J	Supplemental oxygen	–	–	–	–	Beta	–	–	–	5.95	*p* < 0.001 (Omicron /Beta)
	Pfizer or J&J	Supplemental oxygen	–	–	–	–	Delta	–	–	–	4.69	*p* < 0.001 (Omicron /Delta)
Christensen et al.^60^	Pfizer, Moderna or J&J	IMV	–	–	–	5.5	Alpha	–	–	–	8.4	*p* < 0.0001 (Omicron /Alpha)
	Pfizer, Moderna or J&J	IMV	–	–	–	–	Delta	–	–	–	10.7	*p* < 0.0001 (Omicron /Delta)
	Pfizer, Moderna or J&J	NIV	–	–	–	7.1	Alpha	–	–	–	9.5	*p* < 0.0001 (Omicron /Alpha)
	Pfizer, Moderna or J&J	NIV	–	–	–	–	Delta	–	–	–	9.5	*p* < 0.0001 (Omicron /Delta)
	Pfizer, Moderna or J&J	ECMO	–	–	–	0.1	Alpha	–	–	–	0.4	*p* < 0.0001 (Omicron /Alpha)
	Pfizer, Moderna or J&J	ECMO	–	–	–	–	Delta	–	–	–	0.3	*p* < 0.0001 (Omicron /Delta)
	Pfizer, Moderna or J&J	HFOT	–	–	–	8.1	Alpha	–	–	–	21.2	*p* < 0.0001 (Omicron /Alpha)
	Pfizer, Moderna or J&J	HFOT	–	–	–	–	Delta	–	–	–	26.5	*p* < 0.0001 (Omicron /Delta)
	Pfizer, Moderna or J&J	LFOT	–	–	–	35.5	Alpha	–	–	–	42.0	*p* < 0.0001 (Omicron /Alpha)
	Pfizer, Moderna or J&J	LFOT	–	–	–	–	Delta	–	–	–	33.8	*p* < 0.0001 (Omicron /Delta)
Wang et al.^14^	NA	MV	0.33	–	–	–	Delta	1.15	–	–	–	RR 0.29 (0.18–0.46)
Wang et al.^38^	Pfizer, Moderna or J&J	MV	–	–	–	0.07	Delta	–	–	–	0.43	RR 0.16 (0.08–0.32)
Wang et al.^39^	NR	MV	–	–	–	0.08	Delta	–	–	–	0.3	RR 0.25 (0.2–0.31)
Lewnard et al.^51^	Pfizer, Moderna, or J&J	MV	–	–	–	0.1	Delta	–	–	–	0.1	aHR 0.36 (0.18–0.72) overall aHR 0.24 (0.12–0.48) UV aHR 1.50 (0.56–3.99) V
	Pfizer, Moderna, or J&J	MV	–	–	–	0.05 BA2	–	–	–	–	–	–
	Pfizer, Moderna, or J&J	MV	–	–	–	0.01 BA1	–	–	–	–	–	–
Marks et al.^15^	NR	IMV	1.6	5.5	–	–	Delta	7.1	9.4	–	–	–
Fall et al.^5^	Pfizer, Moderna or J&J	Death	0.2	0	0		Delta	1.2	0.7	2.4		*p* < 0.00001 (Omicron/Delta) *p* = 0.1 (Omicron/Delta boosted)
Viellard‐ Baron et al.^13^	Pfizer, Moderna or AstraZeneca	Death	–	–	–	20.0	Delta	–	–	–	27.9	*p* = 0.08 (Omicron /Delta) hospitalized
Modes et al.^20^	mRNA vaccine	Death	4.9	3.4 (2–3D)		–	Delta	7.9	4.9 (2–3D)		–	*p* = 0.02 (Omicron/Delta V) *p* = 0.21 (Omicron /Delta UV) *p* = 0.01 (Omicron/Delta) hospitalized
Abdullah et al.^36^	NR	Death	–	–	–	4.5	Ancestral, Beta, Delta	–	–	–	21.3	*p* < 0.00001 (Omicron /Delta) hospitalized
Maslo et al.^40^	Pfizer or J&J	Death	–	–	–	–	Delta	–	–	–	20.25	*p* < 0.001 (Omicron /Delta)
Jassat et al.^41^	Pfizer or J&J	Death	–	–	–	0.78	D614G	–	–	–	2.73	*p* < 0.001 (Omicron /D614G)
	Pfizer or J&J	Death	–	–	–	–	Beta	–	–	–	3.58	*p* < 0.001 (Omicron /Beta)
	Pfizer or J&J	Death	–	–	–	–	Delta	–	–	–	2.60	*p* < 0.001 (Omicron /Delta)
Krutikov et al.^47^	AstraZeneca, Pfizer or NR	Death	–	–	–	5.3	Delta	–	–	–	12.8	*p* < 0.0001 (Omicron/Delta)
Araujoda Silva et al.^48^	NR	Death	–	–	–	1.7	Pre–Omicron	–	–	–	2.5	*p* = 0.894 (Omicron /pre–Omicron) hospitalized children
Ward et al.^31^	Pfizer, Moderna or AstraZeneca	Death	–	0.02 (1–3D)		–	Delta	–	0.09		–	67% lower (HR = 0.33, (0.24–0.45) for Omicron compared to Delta infections (fully adjusted)^a^
Christensen et al.^60^	Pfizer, Moderna or J&J	Death	–	–	–	0.9	Alpha	–	–	–	5.4	*p* < 0.0001 (Omicron/Alpha)
	Pfizer, Moderna or J&J	Death	–	–	–	–	Delta	–	–	–	5.3	*p* < 0.0001 (Omicron /Delta)
Houhamdi et al.^61^	NR	Death	–	–	–	0.1	Delta	–	–	–	1.3	*p* < 0.0001 (Omicron/Delta)
Lewnard et al.^51^	Pfizer, Moderna, or J&J	Death	–	–	–	0.01	Delta	–	–	–	–	aHR 0.21 (0.10–0.44) overall aHR 0.14 (0.07–0.28) UV aHR 0.25 (0.09–0.70) V
	Pfizer, Moderna, or J&J	Death	–	–	–	0.1 BA2	Delta	–	–	–	0.08	aHR 1.16 (0.25–5.29) overall BA.1 versus BA.2
	Pfizer, Moderna, or J&J	Death	–	–	–	0.13 BA1	–	–	–	–		
Peralta‐ Santos et al.^54^	Pfizer, Moderna, AstraZeneca, or J&J	Death	–	–	–	0	Delta	–	–	–	0.3	*p* < 0.001 (Omicron/Delta) aOR 0.14 (0.0011–1.12) overall Omicron had 86% reduction in odds of death compared to Delta
Ulloa et al.^65^	NR	Death	–	–	–	0.01	Delta	–	–	–	0.54	–
Jassat et al.^41^	Pfizer or J&J	Severe symptoms	–	–	–	2.45	D614G	–	–	–	6.65	*p* < 0.001 (Omicron/D614G)
	Pfizer or J&J	Severe symptoms	–	–	–	–	Beta	–	–	–	7.88	*p* < 0.001 (Omicron/Beta)
	Pfizer or J&J	Severe symptoms	–		–	–	Delta	–	–	–	6.20	*p* < 0.001 (Omicron/Delta)
Martin et al.^43^	NR	Severe symptoms^b^	–	–	–	<11	Pre‐ Omicron	–	–	–	38.8	*p* < 0.001 (Omicron/pre‐Omicron) hospitalized paediatric
Auvigne et al.^59^	Pfizer, Moderna, AstraZenecaor J&J	Severe hospital events (ICU/death)	–	–	–	0.12	Delta	–	–	–	0.89	*p* < 0.001 (Omicron/Delta)
Viellard‐ Baron et al.^13^	Pfizer, Moderna, or AstraZeneca	Pneumonia	80.7	62.1 (1–3D)		–	Delta	97.8	87.5 (1–3D)		–	*p* < 0.001 (Omicron /Delta) hospitalized
Boscolo‐ Rizzo et al.^58^	NR	Smell and taste impairments	–	–	–	19.2	–	–	–	–	–	*p* < 0.001 (Omicron/comparator)
Robinson et al.^21^	Pfizer, Moderna, or J&J	Severe/death	9.35	4	–	–	Alpha	30.84	22.22	–	–	
	Pfizer, Moderna, or J&J	Severe/death	–	–	–	–	Delta	20.77	17.18	–	–	RR 0.78 (0.62–0.97) overall
	Pfizer, Moderna, or J&J	Severe/death	–	–	–	–	Ancestral	1.98	0	–	–	RR 1.04 (0.84–1.24) overall risk difference 0.01 (−0.04–0.06)
	Pfizer, Moderna, or J&J	Severe/death	–	–	–	–	Other	26.5	0	–	–	

^a^ The risk of death involving COVID‐19 was 67% lower (HR = 0.33, 95%CI: 0.24, 0.45) for Omicron compared to Delta infections fully adjusted for sex, age, vaccination status, previous infection, calendar time, ethnicity, Index of Multiple Deprivation rank, household deprivation, university degree, keyworker status, country of birth, main language, region, disability and health risk factors.

^b^ Defined as requiring invasive ventilation, vasopressors, or extracorporeal membrane oxygenation or death.

#### 3.2.1 Hospital admissions

Figure 2 shows the rates of hospital admissions among patients infected with Omicron compared to other variants of SARS‐CoV‐2 Figure 2A–F illustrate these rates in unvaccinated patients, fully vaccinated patients, vaccine‐boosted patients, mixed data for partially and fully vaccinated patients, and those who did not have known/reported vaccination status, respectively. All the studies showed lower admission rates among the Omicron infected patients compared to the other variants, regardless of the vaccination status or doses. Significant differences are reported in the figure title.

#### 3.2.2 ICU admission

Figure 3 shows the rates of ICU admission among patients infected with Omicron compared to other variants of SARS‐CoV‐2. Figure 3A–F illustrates the rates in unvaccinated patients, fully vaccinated patients, vaccine‐boosted patients, mixed data for partially and fully vaccinated patients, and those who did not have a known/reported vaccination status, respectively. All the studies showed lower ICU admission rates among Omicron‐infected patients compared to the other variants, regardless of the vaccination status or doses, except for Marks et al.,^15^ who reported a higher ICU admission rate among the fully vaccinated Omicron‐infected patients without reporting the statistical significance of the difference. Significant differences are reported in the figure title.

#### 3.2.3 Need of oxygenation/ventilation

Figure 4 shows the rates of the need for oxygenation/ventilation among patients infected with Omicron compared to other variants of SARS‐CoV‐2. Figure 4A–F illustrates the rates in unvaccinated patients, fully vaccinated patients, vaccine‐boosted patients, mixed data for partially and fully vaccinated patients, and those who did not have known/reported vaccination status, respectively. All the studies showed lower rates of low‐flow oxygen therapy (LFOT), high‐flow oxygen therapy (HFOT), unspecified supplementary oxygen (USO), invasive mechanical ventilation (IMV), non‐invasive ventilation (NIV), unspecified mechanical ventilation (UMV), and extracorporeal membrane oxygenation (ECMO) among Omicron infected patients compared to the other variants, regardless of the vaccination status or doses, except for Goga et al.,^6^ who reported a significantly higher rate of need for oxygenation among the vaccinated Omicron‐infected patients (dose not reported (NR)) compared to Delta and Lewnard et al.,^51^ who reported similar rates of MV in the Omicron and Delta‐infected patients without reporting their vaccination status. Significant differences are reported in the figure title.

#### 3.2.4 Cardiovascular and/or haematological complications

Figure 5 illustrates the rates of incidence of cardiovascular and/or haematological events among patients infected with Omicron compared to other variants of SARS‐CoV‐2. Figure 4A illustrates the rates among unvaccinated patients infected with Omicron, Alpha, or Delta as reported by one study, which presented lower rates of myocardial infarction (MI) and stroke in Omicron‐infected patients compared to both Alpha and Delta.^10^ Lower rates of venous thromboembolic event (VTEE) in Alpha patients were reported compared with both Omicron and Delta. However, the statistical significance of these differences was not reported. Figure 4B illustrates the rates among fully vaccinated or boosted patients infected with Omicron, Alpha, or Delta as reported by Lauring et al., which showed lower rates of MI in Omicron‐infected patients compared with both Alpha and Delta.^10^ Higher rates of stroke and venous thromboembolism (VTE) were reported in the Omicron‐infected patients compared to Delta. However, the statistical significance of the differences was not reported.

#### 3.2.5 Death

Figure 6 shows the death rates among patients infected with Omicron compared to other variants of SARS‐CoV‐2. Figure 6A–F illustrates the rates in unvaccinated patients, fully vaccinated patients, vaccine‐boosted patients, mixed data for partially and fully vaccinated patients, and those who did not have known/reported vaccination status, respectively. All the studies showed lower death rates among Omicron‐infected patients compared to the other variants, regardless of the vaccination status or doses, except for Lauring et al.,^10^ who reported a higher death rate in the unvaccinated Omicron‐infected patients compared to Alpha without reporting the statistical significance of the difference. Significant differences are reported in the figure title.

#### 3.2.6 Other complications

Some studies reported the rates of combined events without separating the data of each category, such as ICU admission and death, and others described the symptoms as severe without specifying the type of symptoms or intervention. This, in addition to ED visits, respiratory failure, pneumonia, NRRT, lung infiltrates on CXR/CT or the use of vasopressors, were reported in this review under the category of other complications (Table S9). Whenever Omicron was compared with other variants, lower or similar rates were reported for the Omicron‐infected patients compared to the patients infected with other variants. However, the statistical significance of the differences between the variants was tested only in a few studies. For example, Viellard‐Baron et al. reported a significantly lower rate of pneumonia among Omicron‐infected patients compared to Delta‐infected patients (*p* < 0.01, partially, fully vaccinated and boosted).^13^ Jassat et al. reported a significantly lower rate of severe symptoms in Omicron‐infected patients compared to Delta, Beta, and the D614G variants (*p* < 0.001, vaccination status NR).^41^ Similarly, Martin et al.^43^ and Auvigne et al.^59^ found that the rate of incidence of severe symptoms among Omicron‐infected patients was significantly lower than those in pre‐Omicron and Delta patients, without reporting the vaccination status (*p* < 0.001). Some other complications such as ED visits, respiratory failure, pneumonia, NRRT, lung infiltrates on CXR/CT and the use of vasopressors were mentioned for Omicron infected patients in some studies without comparing them with other variants, all of which are summarized in Table S9. Loss/impairment of smell and/or taste in Omicron infected patients has been reported by 9 studies.^30^, ^46^, ^49^, ^53^, ^58^, ^63^, ^66^ The percentage of patients experiencing these symptoms ranged from 1.2% to 24% of the cohorts. They were either known to be vaccinated or their vaccination status was not reported.

## 4 DISCUSSION

Our systematic review included 62 articles that reported the demographic and clinical data of at least 8,385,353 subjects infected with SARS‐CoV‐2, of which 2,733,860 were infected with Omicron. Since the emergence of the Omicron variant, it was not clear how the severity of an Omicron infection compared to other variants, such as the Delta variant. It was not possible to compare the rates of hospitalization, ICU admission, need for oxygenation/ventilation, death, or other severe complications without accounting for multiple factors. For example, Iuliano et al. reported that during the beginning of the Omicron wave in the US, the rates of hospitalizations, death, and ICU admission were 2.7%, 0.9%, and 13.0%, respectively, which were significantly lower than the rates during the Delta wave (*p* < 0.05).^42^ This observed decrease in disease severity could be explained by multiple factors such as increased vaccination coverage,^66^, ^68^ use of vaccine boosters by recommended subgroups,^25^ infection‐acquired immunity,^51^, ^69^ and possibly the decreased virulence of the Omicron variant.^36^, ^51^, ^70^ Receipt of a third mRNA vaccine dose has proven to be highly effective at preventing ED visits and hospital admissions during both the Omicron and Delta predominant periods.^25^ Increases in ED visits and hospital visits during the Omicron period could be attributed to the high case counts and not increased disease severity. The relatively high increases in ED visits and hospitalizations among children during the Omicron wave were likely related to lower vaccination rates among this population. While the Omicron wave saw the highest reported numbers of COVID‐19 cases and hospitalizations compared to previous peaks of the pandemic, the disease is believed to be less severe as evidenced by lower ICU admissions and fatal outcomes.

In this review, we compiled all relevant complications, which have been reported post‐Omicron infections compared with other variants whenever reported. Some studies did not report any severe infections, rather, they specifically reported either asymptomatic or mild Omicron infections.^19^, ^23^, ^64^ Several other studies reported low rates of severe clinical presentation. For example, Dinh et al.,^35^ as well as other studies, confirm that the Omicron surge is highly critical considering the number of cases, although it presents with a lower rate of severe clinical presentation and deterioration. Similarly, Houhamdi et al.^61^ and Modes et al.^20^ suggested less severe infections with Omicron.

### 4.1 Rates of hospitalization and ICU admission among the Omicron infected patients compared to the other variants

Abdullah et al. investigated the clinical severity of the Omicron variant in 466 patients, who were admitted to the hospital in Tshwane with COVID‐19 since November 14, 2021, compared to 3962 patients admitted since May 4, 2020 prior to the Omicron variant outbreak.^36^ The rates of death and ICU admissions were significantly lower during the Omicron wave compared to previous waves. Furthermore, Krutikov et al. compared COVID‐19 cases in long‐term care facility residents in pre‐Omicron and Omicron periods.^47^ They reported a lower risk of hospitalization among Omicron cases compared to Delta (adjusted HR 0.33, *p* = 0.045), as well as a lower death rate. This suggested that the risk of severe outcomes in long‐term care facility residents with the Omicron variant is substantially lower than with other variants. Christensen et al. reported that Omicron‐infected patients were significantly younger, hospitalized less frequently and had shorter median hospital lengths of stay compared to Alpha and Delta variants.^60^ Omicron also demonstrated a greater percentage of breakthrough cases compared to the Alpha and Delta variants. They suggested that the lower age of Omicron‐infected patients might likely be due to riskier behaviour engaged by younger populations (less social distancing and face covering). Veneti et al. reported that Omicron cases were associated with a lower risk of hospitalization and severe disease compared to Delta with hospitalized Omicron‐infected patients showing a milder disease trajectory.^12^

### 4.2 Rates of death among Omicron infected patients compared to other variants

Ward et al. conducted a retrospective cohort study that assessed the risk of mortality, as identified by death certification records, attributed to COVID‐19 in patients who contracted Omicron or Delta in the UK.^31^ After adjusting for a spectrum of possible confounders such as age and vaccination status, there was a 67% reduction (HR = 0.33, 95% CI: 0.24–0.45) of mortality attributed to COVID‐19 with Omicron compared to Delta. When vaccination status was considered, the risk reduction of mortality between the 2 variants was even more significant in individuals who received a booster compared to those who received the primary vaccination series (2 doses) only, regardless of age. Therefore, the study results provide strong evidence that Omicron is associated with a significantly reduced risk of mortality compared to Delta in the UK.

### 4.3 Severity (different severe hospital events) of Omicron compared with other variants

Peralta‐Santos et al. reported that the Omicron variant was associated with 75% and 86% risk reduction of hospitalization and death, respectively, compared to the Delta variant.^54^ Similar results were obtained by Ulloa et al., who matched the Omicron cases with the Delta cases and reported a 65% lower risk of hospitalization or death and 83% lower risk of ICU admissions or death among Omicron infections compared to Delta.^65^

Some studies compared the severity of the different variants while considering the vaccination status and previous infection. Davies et al. compared the fourth Omicron‐driven wave with previous waves in South Africa.^62^ After adjusting for gender, age, comorbidities, prior infections and vaccination status, the study concluded a general patterned decrease in severity as the waves progressed. There was a significantly lower risk of death in the fourth wave compared to the third. The risk of any hospitalization or death was significantly lower for the Omicron wave compared to the Delta and Beta waves. Likewise, Lewnard et al. assessed the severity while accounting for age, sex, health status, history of previous infection and vaccination status and calculated the adjusted HR for the Omicron versus Delta variant for hospital admission, symptomatic hospital admission, ICU admission, mechanical ventilation and mortality to be 0.62 (95% CI: 0.54–0.72), 0.59 (95% CI: 0.51–0.69), 0.45 (95% CI: 0.26–0.78), 0.36 (95% CI: 0.18–0.74), 0.21 (95% CI: 0.10–0.42), respectively.^51^ Wang et al. compared the clinical severity of either Omicron or Delta as a first‐time COVID‐19 infection in the USA.^39^ Propensity‐score matching resulted in 2 variant cohorts with 147,107 patients each. Indicators of severity, including ED visits, hospitalizations, ICU admissions and MV, were compared between the two cohorts (stratified by age groups) and revealed a significant reduction in risk of severe clinical outcomes in the Omicron cohort compared to the Delta cohort across all ages. The findings of Auvigne et al.^59^ and Espenhain et al.^57^ concurred with the above, where the risk of a severe hospital event was found to be lower in those infected with Omicron compared to Delta. Maslo et al. reported a significantly lower rate of hospital admission for Omicron compared to the Ancestral, Beta, Delta, and Omicron waves (*p* < 0.001).^40^ ICU admissions, oxygen therapy, MV and deaths were significantly lower during the Omicron period relative to the previous waves (*p* < 0.001).

### 4.4 Studies reporting comparable severity in patients infected with Omicron or other variants

Unlike many studies that reported lower severity in Omicron cohorts compared to the other variants, Fall et al.^5^ concluded comparable severities in both Omicron and Delta. The study compared the clinical outcomes post‐infection with the Omicron variant compared to the Delta variant in the US population during a period where both variants were codominant. Results were stratified based on vaccination status to compare fully vaccinated patients to boosted patients. Analyses revealed an overall significantly reduced severity of Omicron when compared to Delta because of lower rates of admissions (3.0% vs. 13.8%), ICU (0.5% vs. 3.5%) and deaths (0.1% vs. 1.1%). However, among the admitted patients, there was no significant difference in the use of supplemental oxygen (*p* = 0.5), ICU admissions (*p* = 0.5) and deaths (*p* = 0.5), suggesting the importance of taking proper precautions against the Omicron variant and not underestimating its infectivity. Similarly, Paredes et al. reported that their results reinforce the need for sentinel surveillance, hospital readiness and vaccination due to the lack of statistical significance between hospitalizations due to Omicron and the Ancestral variant.^52^ Contrastingly, Lauring et al. reported that patients hospitalized in the Omicron period had lower severity but still had a substantial risk for severe illness.^10^

### 4.5 Severity (different severe hospital events) in children and adolescent Omicron‐infected patients compared with other variants

AraujodaSilva et al. looked at the severity of COVID‐19 hospitalizations in a total of 300 children in Rio De Janeiro.^48^ In the pre‐Omicron cohort, rates of ICU admission and death were 53.3% and 2.5%, respectively. The rates of the same were 50.9% and 1.7% in the Omicron cohort. Five of the Omicron cases were eligible to receive the vaccine at the time of their infection. Two of them received two doses of Pfizer and two received only one dose. None of the four vaccinated patients were admitted to the ICU and all of them survived the infection. Cloete et al. conducted a multicentre observation study in South Africa involving a pediatric population (age: birth to 18 years) who tested positive for SARS‐CoV‐2 and were hospitalized.^26^ The study aimed to describe the incidence of pediatric hospital admissions in South Africa and report the clinical manifestations and outcomes of hospitalized Omicron cases. During the period defined as the Omicron wave in South Africa, between October 31 and December 11, 2021, the number of pediatric cases recorded was higher than the previous waves. Of 138 pediatric patients—88% required standard inpatient care, 20% required oxygen therapy, 5% required mechanical ventilation, and 3% died. All children included in this study were unvaccinated, and 92% of their parents or guardians were unvaccinated. Ludvigsson et al.^22^ reported three pediatric patients admitted for convulsions that had also tested positive for COVID‐19 in the Omicron era. Patients were aged 3 months, 21 months, and 14 years, of whom the younger two were unvaccinated. The study reported convulsions and gastroenteritis as the two most frequent diagnoses that would lead to hospital admissions in children. Martin et al.^43^ looked at the presence of upper airway infections (UAI) in children infected with Omicron and its potential to develop severe disease. The study found a third of children developed a severe form of the disease, including the need for invasive ventilation and mortality. Marks et al. reported that a higher proportion of adolescents was admitted to the hospital (7.1% compared to 1.8%) and ICU (1.5% compared to 1.1%) with the Omicron variant compared to the Delta variant.^15^ That said, hospital deaths among adolescents were lower (0.6% vs. 0%) with the Omicron variant compared to the Delta variant. Vaccination of eligible persons, in addition to other prevention strategies such as masking, is critical to reducing the incidence of severe COVID‐19 among children and adolescents.

Wang et al. compared the clinical severity of either Omicron or Delta as a first‐time COVID‐19 infection in children under the age of five in the USA.^14^ Propensity‐score matching was done for demographics, socio‐economic determinants of health, comorbidities and medications, resulting in two variant cohorts with 7198 patients each. Severity outcomes of ED visits, hospitalizations, ICU admissions, and mechanical ventilation were compared between the two cohorts, which revealed a significant reduction in risk of severe clinical outcomes in the Omicron cohort compared to the Delta cohort. There was a 29% reduction in ED visits, 67% in hospitalizations, 68% in ICU admissions and 71% in MV. Since the study population was unvaccinated and not previously infected, the potential effects of pre‐existing immunity do not confound the results. Hence, this suggests that Omicron is integrally milder than Delta in the study population.

Goussard et al. reported a case of a 7‐week‐old male infant in South Africa infected with the Omicron variant leading to pediatric ICU admission and ultimately death.^27^ He was born pre‐term at 29‐weeks gestation and required continuous positive airway pressure soon after birth. The baby tested positive for SARS‐CoV‐2 on tracheal aspirate with no other bacteria or viruses identified. He was intubated for respiratory failure and apnoea and was switched to high‐frequency oscillatory ventilation due to bilateral pneumothoraxes, pulmonary interstitial emphysema and increasing oxygenation index. The baby was initially treated with dexamethasone, meropenem, ganciclovir and co‐trimoxazole with no improvement in his condition. He passed away 17 days after admission. The infant's mother was unvaccinated and tested negative for SARS‐CoV‐2 during his illness; however, she had high levels of antibodies against N‐protein likely suggesting a recent infection that could have occurred simultaneously with his illness. On autopsy, the lungs showed a pronounced organizing pneumonia pattern with focal subpleural haemorrhagic lung infarction and thrombosis in the pulmonary arteries and right ventricle. Given these findings, the study suggested the need to measure anticoagulation and d‐dimer in infants with severe SARS‐CoV‐2 pneumonia in addition to the benefit of the antenatal vaccination to protect the foetus via the transfer of humoral immunity from the mother. Therefore, vaccination during pregnancy is recommended to prevent future cases of severe SARS‐CoV‐2 pneumonia in neonates and young infants. Similarly, Vallejo et al. reported two cases of cerebral venous sinus thromboses requiring ICU admission associated with COVID‐19.^45^ The first patient was a 7‐year‐old boy with involvement of the cavernous sinus and bilateral internal jugular veins and the second patient was a 11‐year‐old boy with involvement of the superior sagittal sinus. The first patient was confirmed to have an infection with the Omicron variant, whereas the second patient was assumed to have an infection with the Omicron variant due to a predominance of the variant at the time.

### 4.6 Cardiovascular and/or haematological complications

Only 1 study reported cases of VTE, VTEE, MI and stroke in Omicron‐infected patients, which reflects the rarity of such severe complications in patients infected with Omicron.^10^ However, both Goussard et al.^27^ and Vallejo et al.^45^ reported thrombotic events in three children as discussed in the previous section. Unlike the infections by the Omicron variant, several studies reported many cases of cardiac injury and thrombosis during the pre‐Omicron period. For example, Tomerak et al. described many types of venous and arterial thrombosis in COVID‐19 patients.^71^ Furthermore, a systematic review conducted by Aldien et al. in 2022 reported that of 2204 COVID‐19 patients, 443 adult, children and adolescent patients developed cardiac injury.^72^

### 4.7 Neurological complications

Only 1 study reported 3 paediatric patients admitted for convulsions that had tested positive for COVID‐19 in the Omicron era confirming the presence of neurological symptoms in children with the SARS‐CoV‐2 infection.^22^ However, neurological complications were widely reported in COVID‐19 patients before the Omicron wave, where encephalopathy, neuromuscular disorders and cerebrovascular disorders were commonly reported complications.^73^

### 4.8 Other complications

Some studies reported other types of complications, such as respiratory failure, pneumonia, NRRT, lung infiltrates on CXR/CT and the use of vasopressors. Whenever Omicron was compared with other variants, lower or similar rates were reported for Omicron‐infected patients compared to patients infected with other variants. However, it is important to mention that many of the commonly reported complications before the Omicron era were not reported in Omicron‐infected patients. For example, shock, arrhythmia and acute cardiac injury were identified pre‐Omicron with a prevalence of 8.7%, 16.7% and 7.2%, respectively.^74^ Another study conducted by Zhou et al. in 2019 showed that, of 191 COVID‐19 patients, sepsis was the most common complication with a prevalence of 59%.^75^ Respiratory failure, acute respiratory distress syndrome (ARDS), heart failure and septic shock are other severe complications identified in the study, with a prevalence of 54%, 31%, 23% and 20%, respectively.^75^ Furthermore, the prevalence of kidney injury among patients hospitalized with COVID‐19 varies; however, the highest reported prevalence was 69%.^76^ In fact, in a meta‐analysis of 3062 patients with COVID‐19, 25.5% had an abnormal renal function.^77^ None of the above was identified as common complications of the Omicron variant.

Loss/impairment of smell and/or taste in Omicron‐infected patients has been reported by seven studies.^30^, ^46^, ^49^, ^53^, ^58^, ^63^, ^66^ The proportions of patients that experienced these symptoms ranged from 1.2% to 24% of the cohorts, which were reported as either vaccinated or the vaccination status was not reported. Boscolo‐Rizzo et al. reported that the prevalence of chemosensory dysfunction was found to be significantly lower during the Omicron wave compared to the control.^58^ The severity of chemosensory dysfunction was also found to be lower among cases in the Omicron period compared to the control period. One of the reasons hypothesized was that the vaccines could have impacted the clinical expression of SARS‐CoV‐2 infections.^78^ In conclusion, the prevalence and severity of chemosensory dysfunction among Omicron cases were significantly decreased compared to other variants of SARS‐CoV‐2.

### 4.9 Are the COVID‐19 vaccines effective against the Omicron variant?

A recent study suggested that COVID‐19 vaccines are effective in reducing the incidence of infections, hospital admissions, fatal outcomes as well as the severity of the infection.^79^ The Pfizer/BioNTech vaccine was found to be especially effective against pre‐Omicron variants B.1.1.7 and B.1.351.^79^ Furthermore, Abu Raddad et al. also reported that individuals who received the Pfizer booster vaccination had a reduced incidence of symptomatic infection with the Delta variant by 86% and with the Omicron variant by 50%.^7^ There was a 51% reduction of symptomatic Omicron infection in the population that received the Moderna booster dose. In conclusion, mRNA boosters are highly effective against infection with the Delta variant and less effective against the Omicron variant.^7^ Similar results were obtained by Accorsi et al. who reported that among 70,155 symptomatic adults, the incidence of complete vaccination with 3 doses of the mRNA vaccine was significantly lower than those who were unvaccinated in both the Omicron cases (OR, 0.33) and the Delta cases (OR, 0.065) when compared to the case‐negative control groups.^64^ This pattern was also seen when individuals with 3 doses were compared to those with two doses (Omicron OR, 0.34; Delta OR, 0.16). These results suggest that complete vaccination with three doses (compared to unvaccinated or vaccinated with only two doses) is associated with protection against the Omicron and Delta variants, with relatively lower protection against the Omicron variant as is suggested by the higher OR for association with Omicron infection. Brandal et al. reported that the Omicron variant of concern is a highly transmissible variant of SARS‐CoV‐2, even among fully vaccinated people, and that vaccination may be less effective in preventing infection compared to the Delta variant.^46^ Similarly, Klein et al. reported that the COVID‐19 vaccines were less effective against Omicron infections.^67^ Modes et al. reported that the 2‐dose vaccination showed decreasing protection from infection for Omicron compared to Delta, likely due to ‘relative resistance’ to Omicron neutralization and waning immunity.^20^ Those who received the booster dose exhibited decreased likelihood of ICU admissions, mechanical ventilation, and death. Veneti et al. reported that two‐dose vaccination between 7 and 179 days prior to infection had a lower protective effect for Omicron cases compared to Delta, while a booster dose had similar protective effects for both Omicron and Delta.^12^

### 4.10 Why are the COVID‐19 vaccines less effective against Omicron?

Riesgo‐Ferreiro et al. reported that Omicron exhibits numerous amino acid substitutions that result in increased immunogenicity.^80^ Studies show that Omicron shares numerous mutations and deletions with other strains that are well‐known for increasing viral transmissibility and binding affinity. It is also hypothesized that these mutations assist with immune evasion and antibody escape.^81^ For example, the combination of Q498R and N501Y mutations may facilitate the viral binding to angiotensin‐converting enzyme (ACE2) leading to viral invasion. In addition, some mutations may result in deletion of certain amino acids in the N‐terminal domain of the spike protein. This N‐terminal domain of the spike protein is a popular target for neutralizing antibodies, so the deletions introduced in that region alter its structure and prevent neutralizing antibodies from binding to it.^81^ This enables the virus to evade the immune response elicited from previous infections or vaccination.^81^ Despite these findings, there is still much uncertainty regarding the exact reason and mechanism Omicron is able to evade both natural and vaccine‐induced immunity.^82^

### 4.11 Why is Omicron less severe than the other variants?

Jassat et al. suggested that the decreased severity among Omicron infected patients is a combination of decreased virulence, increased vaccination rate, as well as immunity from previous infection.^41^ They also conclude that as the variants appear to evolve independently, it is not possible to predict the virulence of future variants. In comparing the Omicron variant to other variants, one of the most noticeable differences is that Omicron infects the upper respiratory tract while other strains infect the lower respiratory tract. Another finding was the inability of Omicron to bind to TMPRSS2, which protrudes from the surfaces of many cells in the lungs and other organs and is exploited by other variants to infect cells. With throat and nose cells lacking this protein, Omicron is able to invade the cells simply by endocytosis.^83^ The fact that Omicron does not invade the lungs makes it less severe than other strains, despite its greater transmissibility. A study conducted in hamsters showed that the Rpef and SpO_2_ values in Omicron infected hamsters were similar to those in uninfected subjects. This is evidence of less pathogenicity of Omicron than the B.1.1 and Delta variants.^3^ The authors characterized this by the fact that Omicron does not invade the lower respiratory tract, which makes it less harmful to humans. It is important to make a distinction between Omicron BA.1 and its subvariant, BA.2, which surpassed the original strain in March 2022, becoming the predominant variant in the United States.^82^ The increased transmissibility of Omicron BA.2 could be attributed to the dozens of mutations that distinguish BA.1 from BA.2, particularly, those mutations at key regions of the virus' spike protein, which are targeted by antibodies to block infection.^84^ These mutations on the spike protein would allow immune evasion, especially in those who have been on the spike protein. This would increase the virus' infectivity by lending it the ability to evade immune responses, particularly in those who have been previously infected but not vaccinated.^82^ Despite the increased transmissibility of the Omicron subvariant, a study published in Nature in 2022 showed that the severity of the BA.2 subvariant is similar to that of the original BA.1 strain.^85^

## 5 STUDY LIMITATIONS

There are some limitations for this study, which are related to the types of reported data in the included studies. The statistical significance of the differences in rates of infection, hospitalization, ICU admission, mortality, etc. between Omicron and the other variants were not reported by many studies. To overcome this problem, we compiled all the data that were statistically tested in Table 1, whether they were significant or not. The extraction of data from different databases may account for some overlap between studies in case the data was published to multiple databases. To overcome this problem, we presented the data that compares the severity among the different variants separately as reported in each study without compiling them. In some studies, when the rates of certain events (such as hospital admissions or death) were calculated, It was not clear whether they were caused directly by the COVID‐19 infections or by other factors. This limitation was also observed in the initial global reports of COVID‐19 clinical data. Some studies described some symptoms as mild, moderate, or systemic without specifying the types of events. In such cases, non‐objective data was excluded and we focused instead on the well‐defined events that were described in the ‘data analysis’ section. Another challenge was the overlap between the rates of some reported events, such as ICU admission and ventilation. For this reason, we avoided discussing the relative rates of incidence of certain events within each cohort as the percentages do not add to 100% due to the overlap.

## 6 CONCLUSION AND RECOMMENDATIONS

The compiled results demonstrated that the severity of the Omicron variant is less than that of the previous waves, including lower rates of hospitalization, ICU admissions, death, and need for oxygenation/ventilation. Some studies, however, still reported comparable severity in patients infected with Omicron as to other variants emphasizing that while the severity of infections in patients hospitalized during the Omicron wave may have been lower, there still was a very real and substantial risk for severe illness. Of note, Omicron‐infected patients were generally younger than patients infected with the other variants. It was suggested by several studies that the younger age of Omicron‐infected patients is likely due to riskier behaviours engaged in by younger populations such as less social distancing and mask‐wearing. While several studies reported that the COVID‐19 vaccines were less protective against severe infections caused by Omicron compared to the other variants including Delta, it was found that a booster dose provided similar protective effects against both Omicron and Delta. Interestingly, while reports of cardiovascular and/or haematological complications in Omicron‐infected patients were rare, it was suggested by one study that there is a need to study anticoagulation and d‐dimer measurement in infants with severe SARS‐CoV‐2 pneumonia. The study also suggested that antenatal vaccination is protective to the foetus due to the transfer of humoral response from the mother. Therefore, vaccination during pregnancy is recommended and may help prevent future cases of severe SARS‐CoV‐2 pneumonia in neonates and young infants.

## AUTHOR CONTRIBUTIONS

**Maryam A. Arabi:** Conceptualization (equal); data curation (lead); formal analysis (lead); writing – original draft (equal); writing – review and editing (equal). **Yousef Al‐Najjar:** Conceptualization (equal); data curation (lead); formal analysis (lead); writing – original draft (equal); writing – review and editing (equal). **Nada Mhaimeed:** Conceptualization (equal); data curation (lead); formal analysis (supporting); writing – original draft (lead); writing – review and editing (equal). **Mohammad Salameh:** Conceptualization (lead); data curation (lead); formal analysis (supporting); writing – original draft (equal); writing – review and editing (lead). **Pradipta Paul:** Conceptualization (lead); data curation (equal); writing – original draft (supporting); writing – review and editing (supporting). **Jamal AlAnni:** Conceptualization (equal); data curation (equal); writing – original draft (equal); writing – review and editing (supporting). **Ali A. Abdelati:** Data curation (lead); formal analysis (equal); writing – original draft (equal); writing – review and editing (supporting). **Ibrahim Laswi:** Conceptualization (equal); data curation (equal); writing – original draft (equal); writing – review and editing (supporting). **Bushra Khanjar:** Conceptualization (equal); data curation (equal); writing – original draft (equal); writing – review and editing (supporting). **Dana Al‐Ali:** Conceptualization (equal); data curation (equal); writing – original draft (equal); writing – review and editing (supporting). **Abdallah Elshafeey:** Conceptualization (equal); data curation (equal); writing – original draft (equal); writing – review and editing (supporting). **Omar Mahimeed:** Conceptualization (supporting); data curation (equal); writing – original draft (equal); writing – review and editing (supporting). **Zain Burney:** Conceptualization (equal); data curation (equal); writing – original draft (supporting); writing – review and editing (supporting). **Ashton D'Souza:** Conceptualization (supporting); data curation (equal); writing – original draft (equal); writing – review and editing (supporting). **Pratyaksha Sinha:** Conceptualization (supporting); data curation (equal); writing – original draft (equal); writing – review and editing (supporting). **Mohammad Bhatti:** Conceptualization (supporting); data curation (equal); writing – original draft (equal); writing – review and editing (supporting). **Krishnadev V. Pillai:** Conceptualization (supporting); data curation (equal); writing – original draft (equal); writing – review and editing (lead). **Moayad Homssi:** Conceptualization (equal); data curation (equal); writing – original draft (supporting); writing – review and editing (supporting). **Khalifa Bshesh:** Data curation (equal); writing – original draft (equal); writing – review and editing (equal). **Lina Yagan:** Data curation (equal); writing – original draft (supporting); writing – review and editing (supporting). **Dalia Zakaria:** Conceptualization (lead); data curation (lead); formal analysis (lead); investigation (lead); methodology (lead); project administration (lead); supervision (lead); writing – original draft (lead); writing – review and editing (lead).

## CONFLICT OF INTEREST STATEMENT

The authors declare no conflict of interest.

## Supporting information


**TABLE S1** Demographic data from studies that reported vaccination status in cases infected with Omicron or other variants.

**TABLE S2** Clinical data from studies that reported vaccination status in cases infected with Omicron or other variants.

**TABLE S3** Demographic and clinical data from studies that did not report vaccination status in cases infected with Omicron or other variants.

**TABLE S4** Hospital admissions reported according to the vaccination status in cases infected with Omicron or other variants.

**TABLE S5** ICU admissions reported according to the vaccination status in cases infected with Omicron or other variants.

**TABLE S6** Cases in need of oxygenation/ventilation reported according to the vaccination status in cases infected with Omicron or other variants.

**TABLE S7** Cases of cardiovascular and haematological complications reported according vaccination status in cases infected with Omicron or other variants.

**TABLE S8** Death cases reported according to the vaccination status in cases infected with Omicron or other variants.

**TABLE S9** Cases with other complications reported according to number of doses in cases with Omicron and other variants.

Data S1

## Data Availability

The data that supports the findings of this study are available in the supplementary material of this article.
