# Supplementary material for: Severity of the Omicron SARS‐CoV‐2 variant compared with the previous lineages: A systematic review
Source: J Cell Mol Med. 2023 May 18;27(11):1443–64. doi: 10.1111/jcmm.17747 (PMC10243162; doi:10.1111/jcmm.17747)
Supplement: Supplementary file 1 — TABLE S1 Demographic data from studies that reported vaccination status in cases infected with Omicron or other variants. [file JCMM-27-1443-s009.docx]

**Supplementary Table 1:** **Demographic data from studies that reported vaccination status in cases infected with Omicron or other variants**

| **Author(s) and study**  **Source of data**  **Type of population** | **Type of study (country)** | **Unvaccinated** | | **Vaccinated** | | **Notes** |
| --- | --- | --- | --- | --- | --- | --- |
|  |  | **N (number of subjects) and gender (F/M)** | **Age mean±SD or median (IQR)** | **N (number of subjects) and gender (XF/YM)** | **Age mean±SD or median (IQR)** |  |
| Fall et al. ^5^  Johns Hopkins Health System  General population | Cross-sectional cohort study  (USA) | 488 | 31.8 (18.8) | 461 | 32 (17.9) | Patients infected with Omicron were younger than those infected with Delta (p<0.001). |
| - | - | - | - | 134 | 34 (19.3) |  |
| - | - | 518 | 43 (23.6) | 282 | 36 (22.6) |  |
| - | - | - | - | 83 | 39 (25.1) |  |
| Goga et al. ^6^  Vaccination centers, across all provinces of South Africa  Healthcare workers | Multicenter, cross-sectional cohort study  (South Africa) | NR | NR | 26393 (20655/5738) | NR | Age was reported as follows:  18-30: 5342  31-54: 17583  55+: 3467 |
| - | - | NR | NR | 15195 (11540/3655) | NR | Age was reported as follows:  18-30: 2214  31-54: 9919  55+: 3062 |
| Abu-Raddad et al. ^7^  National database of Qatar  General population | Retrospective cohort study (Qatar) | NR | NR | 10,994 | NR |  |
| - | - | NR | NR | 7,210 | NR |  |
| - | - | NR | NR | 1,880 | NR |  |
| - | - | NR | NR | 1,436 | NR |  |
| BirolIlter et al. ^8^  Tertiary care facilities in Istanbul, Turkey and London, UK  Pregnant women | Retrospective cohort study (Turkey and UK) | 52 (52/0) | 31.0 (26.0–35.0) | 83 (83/0) | 31.0 (28.0–34.0) |  |
| Kuhlmann et al. ^9^  Diagnostic laboratories  Visitors | Case series (South Africa) | NR | NR | 7 (5/2) | 27.7**±**5.06 |  |
| Lauring et al. ^10^  Vaccine records from clinics and pharmacies  Hospitalized adults | Prospective, case-control study  (United States) | 953 (519/553)* | 56* (43-65.5)* | 119 (519/553)* | 56* (43-65.5)* |  |
| - | - | 2871 (1803/2148)* | 57* (43-69)* | 1080 (1803/2148)* | 57* (43-69)* |  |
| - | - | 268 (264/295)* | 62* (49-73)* | 291 (264/295)* | 62* (49-73)* |  |
| Lee et al. ^11^  Montefiore Health System  Cancer patients | Cross-sectional cohort study  (United States) | 56 (151/134)* | 52 | 205 (151/134)* | 64 | Age was reported as a median. |
| - | - | - | - | 9 (151/134)* | NR |  |
| Veneti et al. ^12^    Norwegian emergency preparedness registry for COVID19 (Beredt C19)    Normal population | Retrospective Cohort Study  (Norway) | 8046 (19685/19839)* | 29* (14-44)* | 2734 (19685/19839)* | 29* (14-44)* | Age median (IQR) reported overall, not by vaccination or variant |
| - | - | - | - | 25401 (19685/19839)* | 29* (14-44)* |  |
| - | - | - | - | 3343 (19685/19839)* | 29* (14-44)* |  |
| - | - | 22915 (25577/25904)* | 29* (14-44)* | 4290 (25577/25904)* | 29* (14-44)* |  |
| - | - | - | - | 22771 (25577/25904)* | 29* (14-44)* |  |
| - | - | - | - | 1505 (25577/25904)* | 29* (14-44)* |  |
| Vieillard-Baron et al. ^13^  APHP Reality registry    Hospitalized patients | Retrospective Cohort Study  (France) | 83 (75/154)* | 63* (49-71)* | 132 (75/154)* | 63* (49-71)* | Total patients were 888, but 161 patients had no variant info, 98 patients didn’t have enough viral load.  15 patients with known variants had unknown vaccination status |
| - | - | 279 (135/265)* | 62* (51-70)* | 120 (135/265)* | 62* (51-70)* |  |
| Wang et al. ^14^  TriNetX Analytics    Pediatric patients | Retrospective Cohort Study  (United States) | **After matching:**  7,198 (3,412 /3,786)  **Before matching:**  7,201 (3,413/3,788) | **After matching:**  1.49**±**1.42  **Before matching:**  1.49±1.42 | NR | NR |  |
| - | - | **After matching:**  7,198 (3,426/3,765)  **Before matching:**  63,203(29,326 F/33877 M) | **After matching:**  1.48**±**1.42  **Before matching:**  1.73**±**1.41 | NR | NR | 7 unknown cases after matching |
| Marks et al. ^15^  Coronavirus Disease 19–Associated  Hospitalization Surveillance Network  Hospitalized children and adolescents | Cohort study (USA) | 584 (318/266) | 15 (14-17) | 53 (33/20) | 15 (14-16) | Partially vaccinated (1 dose) was grouped with unvaccinated. |
| - | - | 63 (31/32) | 14 (13-16) | 18 (13/5) | 15 (14-16) |  |
| Maisa et al. ^16^  Regional Health Agencies  General population | Cohort study (France) | 92 (196/161)* | 35* | 302 (196/161)* | 35* | Total cases reported were 468 (vaccination status was reported for 394 patients only and F/M ratio was reported for 357 patients only) |
| Maruki et al. ^17^  General population | Case report  (Japan) | NR | NR | 1 (0/1) | 30s |  |
| - | - | NR | NR | 1 (0/1) | 20s |  |
| Loconsole et al. ^18^  Laboratory of Molecular Epidemiology and Public Health of the Hygiene Unit  Healthcare workers | Case series  (Italy) | NR | NR | 15 (10/5) | 31.73**±**5.27 |  |
| Micheli et al. ^19^  Clinical Laboratory of Microbiology, Virology and Bioemergencies of L. Sacco University Hospital  General Population | Case Report  (Italy) | NR | NR | 1 (0/1) | 48**±**0 |  |
| Modes et al. ^20^  EHRs at academic hospital in LA, California  General Population | Retrospective Cohort Study  (United States) | 416 (195/221) | 64 (48–78) | 292 (150/142) | 69 (51–80) |  |
| - | - | 241 (111/130) | 54 (38-68) | 85 (33/52) | 71 (5-82) |  |
| Robinson et al. ^21^  JH-CROWN, a registry of electronic medical record (EMR) data from Johns Hopkins Medicine (JHM)  Hospitalized patients | Retrospective, Observational cohort Study  (United States) | 2116 (1042/1074) | 65.0 (52.0-78.0) | 5 (3/2) | 69.0 (58.0-79.0) | Vaccinated column includes both vaccinated patients (2) and previously infected (3) (overlap).  Age and gender were not stratified for previously infected vs vaccinated. |
| - | - | 214 (110/104) | 59.0 (47.2-67.0) | 9 (6/3) | 59.0 (55.0-66.0) | Vaccinated column includes both vaccinated patients (8) and previously infected (1) (they overlap).  Age and gender were not stratified for previously infected vs vaccinated. |
| - | - | 568 (280/288) | 55.0 (41.0-67.0) | 291 (141/150) | 68.0 (59.0-80.0) | Vaccinated column includes both vaccinated patients (290) and previously infected (2) (they overlap).  Age and gender were not stratified for previously infected vs vaccinated. |
| - | - | 433 (224/209) | 62.0 (45.0-73.0) | 500 (230/270) | 69.0 (57.0-79.0) | Vaccinated column includes both vaccinated patients (499) and previously infected (1) (they overlap).  Age and gender were not stratified for previously infected vs vaccinated. |
| - | - | 34 (18/16) | 61.0 (50.2-66.2) | 3 (2/1) | 74.0 (65.5-82.5) | Vaccinated column includes both vaccinated patients (2) and previously infected (1) (they overlap).  Age and gender were not stratified for previously infected vs vaccinated. |
| Ludvigsson et al. ^22^  Department of Paediatrics at Orebro University Hospital, Orebro, Sweden  General pediatric population | Case Series  (Sweden) | 2 (0/2) | 12**±**12.7 | 1 (0/1) | 14±0 | Vaccinated child had a negative PCR test, but he was diagnosed with “clinical CoVid-19” because 3/3 of his family members tested positive. |
| Piersiala et al. ^23^  Karolinska University Hospital, Stockholm  Patients with odynophagia | Retrospective single-center case study  (Sweden) | 4 (9/11)* | 32±10* | 1 (9/11)* | 32±10* |  |
| - | - | **-** | - | 13 (9/11)* | 32±10* |  |
| - | - | **-** | - | 2 (9/11)* | 32±10* |  |
| Tseng et al. ^24^  US pharmacy-based testing program  General population | Case-control study  (United States) | NR | NR | 2942 (1550/1392) | 41.24±14.29 |  |
| - | - | NR | NR | 4117 (2224/1893) | 42.31±14.64 |  |
| - | - | NR | NR | 3021 (1594/1427) | 41.81±14.67 |  |
| - | - | NR | NR | 8947 (4996/3951) | 37.40±13.06 |  |
| - | - | NR | NR | 19395 (11124/8271) | 39.10±13.77 |  |
| - | - | - | - | 11217 (6345/4872) | 40.61±15.08 |  |
| Thompson et al. ^25^  VISION Network  General population | Multicenter cohort study (United States) | 36542 (29222/24497)” | NR | 10162 (29222/24497)” | NR | Age was reported as ranges.  18-49: 29494  50-64: 12435  65-74: 6492  75-84: 3723  >=85: 1575 |
| - | - | - | NR | 469 (29222/24497)” | NR |  |
| - | - | 3398 (29222/24497)” | NR | 2628 (29222/24497)” | NR |  |
| - | - | - | NR | 520 (29222/24497)” | NR |  |
| Cloete et al. ^26^  National electronic vaccination data system  Pediatric patients | Multicentre observational cohort study (South Africa) | 138 (61/77) | 4.2±4.1 | NR | NR |  |
| Goussard et al. ^27^  Paediatric Intensive Care Unit (PICU)  Pediatric population | Case report (United States) | 1 (0/1) | 7 weeks | NR | NR | Although no vaccination status was clearly mentioned since the patient was 7 weeks old, it was assumed he was unvaccinated. |
| Ferdinands et al. ^28^  VISION Network  Emergency department and urgent care patients | Test-negative case-control study  (United States) | 29063 (34,397/27,429)” | NR | 8136 (34,397/27,429)” | NR | Age ranges:  18-44: 30118  45-64: 18873  65-74: 6868  75-84: 4087  >=85: 1880 |
| - | - | - | NR | 347 (34,397/27,429)” | NR |  |
| - | - | 13991 (34,397/27,429)” | NR | 8351 (34,397/27,429)” | NR |  |
| - | - | - | NR | 1938 (34,397/27,429)” | NR |  |
| Gray 2021 et al. ^29^  350 vaccination centres and 43 clinical research sites  Healthcare workers | Cohort study (South Africa) | 18294 (11591/8430)* | NR | 1727 (11591/8430)* | NR | Age reported as ranges:  18-29: 5605  30-39: 7649  40-49: 3847  50-59: 1750  60-69: 703  70-79: 323  80+:144  All patients received 2 doses. The paper reports the number of patients who got infected 0-13 days after 2nd dose, 14-27 days after 2nd dose, and 27-87 days after 2nd dose. |
| Helmsdal et al. ^30^  Chief Medical Officer’s office  Healthcare workers | Cohort study  (Denmark) | NR | NR | 21 | 45 | Age was reported as median |
| Ward et al. ^31^  NHS Test and Trace PCR positive tests; Lighthouse laboratories  General population | Retrospective cohort study  (United Kingdom) | NR | NR | 221,146  (119,640/101,506) | NR |  |
| - | - | NR | NR | 814,003 (437,120/376,883) | NR |  |
| Sami et al. ^32^  COVID-19 surveillance systems and online survey  General population | Cross-section cohort study  (United States) | NR | NR | 119 (62/54) | 26.1 (22.2-31.4) | Some patients’ data were missing.  These results were based on a surveillance system after event-associated infections. |
| - | - | NR | NR | 48 (31/13) | 28 (23.0-35.0) | These results were based on an online survey. |
| Patalon et al. ^33^  Central  Bureau of Statistics  General population | Test-negative case-control design  (Israel) | NR | NR | 101737 | NR |  |
| - | - | NR | NR | 16938 | NR |  |

General abbreviations: NR: Not reported.

* Not stratified on vaccination status

” Not stratified on vaccination status or COVID-19 variant

- Data is same as data in row above
