# Supplementary material for: Severity of the Omicron SARS‐CoV‐2 variant compared with the previous lineages: A systematic review
Source: J Cell Mol Med. 2023 May 18;27(11):1443–64. doi: 10.1111/jcmm.17747 (PMC10243162; doi:10.1111/jcmm.17747)
Supplement: Supplementary file 2 — TABLE S2 Clinical data from studies that reported vaccination status in cases infected with Omicron or other variants. [file JCMM-27-1443-s001.docx]

**Supplementary Table 2: Clinical data from studies that reported vaccination status in cases infected with Omicron or other variants**

| **Author(s) & study** | **Variant** | **Unvaccinated** | | | **Vaccinated** | | | | **Notes / conclusion made by this study** |
| --- | --- | --- | --- | --- | --- | --- | --- | --- | --- |
|  |  | **Comorbidities % and name of disease** | **Previously infected % and variant** | **Severity %** | **Comorbidities % and name of disease** | **Type of vaccine** | **Previously infected % and variant** | **Severity %** |  |
| Fall et al. ^5^ | Omicron | HTN: 22.3%  Pregnancy: 6.1%  Lung disease: 22.7%  Kidney disease: 6.1%  IC: 10.5%  Diabetes: 8.8%  HF: 2.7%  AF: 1.6%  Smoker: 9.2%  CVD: 4.1%  Cancer: 21.1%  CAD: 6.4% | NR | SY: 91.1%  ASY: 8.9%  Admission: 1.8%  ICU: 0.4%  Death: 0.2% | HTN: 20.6%  Pregnancy: 8.0%  Lung disease: 18.4%  Kidney disease: 6.5%  IC: 11.1%  Diabetes: 9.8%  HF: 3.0%  AF: 1.5%  Smoker: 11.7%  CeVD: 4.3%  Cancer: 22.6%  CAD: 8.9% | 2 doses of Pfizer of Moderna or 1 dose J&J/ Janssen | NR | ASY: 8.9%  SY: 91.1%  Admission: 4.3%  ICU: 0.4%  Death: 0% | General conclusions:  Patients with Omicron infections (N= 1121) were more likely to be vaccinated compared to patients with Delta (N = 910), but were less likely to be admitted (p<0.00001), require ICU level care (p<0.00001), or have fatal outcomes (p=0.004). No statistically significant differences in infectious virus recovery between the boosted, fully vaccinated and unvaccinated groups.  Definitions:  Symptomatic and asymptomatic were not defined. ICU & death were not clarified to be a part of admissions or not. |
| - | Omicron | - | NR | - | HTN: 23.9%  Pregnancy: 9.7%  Lung disease: 16.4%  Kidney disease: 4.5%  IC: 11.2%  Diabetes: 11.2%  HF: 0.7%  AF: 1.5%  Smoker: 7.5%  CeVD: 3.0%  Cancer: 28.4%  CAD: 7.5% | 3 doses | NR | ASY: 5.2%  SY: 94.8%  Admission: 2.2%  ICU 0.7%  Death: 0% | Vaccine types:  Pfizer/BioNTech: 68.6%  Moderna: 26.6%  J&J/Janssen: 4.8%  Statistical significance (Omicron boosted and unvaccinated):  Admission: p=0.7  ICU: p=0.5  Death: p=1.0 |
| - | Delta | HTN: 28.8%  Pregnancy: 5.2%  Lung disease: 22.2%  Kidney disease: 13.9%  IC: 16.6%  Diabetes: 14.3%  HF: 5.8%  AF: 5.2%  Smoker: 16.2%  CVD: 7.9%  Cancer: 21.6%  CAD: 17.0% | NR | ASY: 6.2%  SY: 74.3%  Admission: 15.4%  ICU: 4.6%  Death: 1.2% | HTN: 34.8%  Pregnancy: 7.4%  Lung disease: 22.0%  Kidney disease: 12.1%  IC: 16.0%  Diabetes: 15.2%  HF: 8.5%  AF: 4.3%  Smoker: 14.9%  CeVD: 7.4%  Cancer: 23.0%  CAD: 16.0% | 2 doses of Pfizer or Moderna or 1 dose J&J/ Janssen | NR | ASY: 9.6%  SY: 90.4%  Admission: 10.6%  ICU: 2.1%  Death: 0.7% | Statistical significance (Delta boosted and unvaccinated):  Admission: p=1.0  ICU: p=0.6  Death: p=0.3  Statistical significance (Omicron and Delta boosted):  Admission: p=0.0001  ICU: p=0.6  Death: p=0.1 |
| - | Delta | - | NR | - | HTN: 33.7%  Pregnancy 3.6%  Lung disease: 26.5%  Kidney disease: 19.3%  IC: 24.1%  Diabetes: 20.5%  HF: 7.2%  AF: 7.2%  Smoker: 13.3%  CeVD: 6.0%  Cancer: 31.3%  CAD: 18.1% | 3 doses | NR | ASY: 10.8%  SY: 89.2%  Admission: 15.7%  ICU: 2.4%  Death: 2.4% |  |
| Goga et al. ^6^ | Omicron | NR | NR | NR | Cancer: 1.5%  TB: 0%  HIV: 8.7%  HTN: 21.3%  Diabetes: 10.4%  CLD: 0.6% | 1 or 2 doses of J&J | 0.16% (Beta)  4.5% (Delta) | Admission: 3.34%  High care: 0.21%  ICU: 0.11%  Oxygenation: 0.52%  Ventilation: 0.05% | Sample calculations:  Admissions = # of HCW hospitalized/total infected = 882/26393 = 3.34%  Notes/Definitions:  The percentages of comorbidities are specific to the hospitalized healthcare workers only. Percentages for severity indicators were calculated from the total numbers of infected HCWs, but the p-values are specific to hospitalized HCWs only.  Admissions includes patients in ICU and high care. High care was not defined.  Values for previously infected patients were specific to the first 30 day period. |
| - | Delta | NR | NR | NR | Cancer: 0.8%  TB: 0.1%  HIV: 5.6%  HTN: 34.7%  Diabetes: 22.6%  CLD: 1.2% | 1 or 2 doses of J&J | NR | Admission:  5.59 %  High care: 0.25%  ICU: 0.37%  Oxygenation: 2.26%  Ventilation: 0.40% | Sample calculations:  Admissions = # of HCW hospitalized/total infected = 850/15195 = 5.59 % |
| Abu-Raddad et al. ^7^ | Omicron | NR | NR | NR | NR | 2 doses Pfizer | 0% | ASY: 64.91%  SY: 35.09%  Admissions: 0.14%  Critical: 0%  Death: 0% | Definitions:  SY was reported as symptoms compatible with respiratory tract infections. Severe cases are those who required acute care admission.  Critical COVID-19 disease was defined per WHO classification as a SARS-CoV-2 infected  person with “acute respiratory distress syndrome, sepsis, septic shock, or other conditions that  would normally require the provision of life sustaining therapies such as mechanical ventilation (invasive or non-invasive) or vasopressor therapy” |
| - | Omicron | NR | NR | NR | NR | 3 doses Pfizer | 0% | ASY: 71.78%  SY: 28.22%  Admissions: 0%  Critical: 0%  Death: 0% | Notes:  This paper also reported vaccine effectiveness against Delta and Omicron variant, but no other data was provided. |
| - | Omicron | NR | NR | NR | NR | 2 doses Moderna | 0% | ASY: 68.67%  SY: 31.33%  Admissions: 0%  Critical: 0%  Death: 0% |  |
| - | Omicron | NR | NR | NR | NR | 3 doses Moderna | 0% | ASY: 78.33%  SY: 21.67%  Admissions: 0%  Critical: 0%  Death: 0% | Sample calculations:  ASY = # of asymptomatic cases/total infected patients = 1146/1463 = 78.33%  SY = # symptomatic cases/total infected patients = 317/1463 = 21.67% |
| BirolIlter et al. ^8^ | Omicron | HT: 7.7%  Asthma: 3.8%  Prepregnancy diabetes: 0%  Malignancy: 0%  IC: 0%  Pregnancy: 100% | NR | ASY/Mild: 90.4%  Moderate/Serious: 9.6%  ICU: 3.8%  Oxygen Support: 9.6%  Nasal oxygen support: 3.8%  NIV: 3.8%  IMV: 1.9%  ECMO: 0%  Death: 0% | HT: 7.2%  Asthma: 7.2%  Prepregnancy diabetes: 2.4%  Malignancy: 1.2%  IC: 1.2%  Pregnancy: 100% | 2+ doses of mRNA, inactive, or mixture of vaccines | NR | ASY/Mild: 100%  Moderate/serious: 0%  ICU: 0%  Oxygen Support: 0%  Nasal oxygen support: 0%  NIV: 0%  IMV: 0%  ECMO: 0%  Death: 0% | Definitions:  Mild cases had symptoms of COVID-19 without lower respiratory tract involvement (no dyspnea or abnormal lung imaging  Moderate: lower respiratory tract involvement without significant hypoxemia. Severe cases showed signs of hypoxemia, as evidenced by oxygen saturation (<94%)or imaging showing lung infiltrates >50%.  Percent of oxygen support included patients who needed needed nasal oxygenation, NIV, IMV, and ECMO.  Statistical significance (Omicron vaccinated and unvaccinated):  Oxygen support: p=0.015  Nasal oxygen support: p=0.285  NIV: p=0.285  IMV: p=0.812  Vaccination status:  Number of doses taken by vaccinated patients:  2 doses: 84.34%  3+ doses: 15.66%  Vaccine types:  mRNA: 94.0%  Inactivated: 2.4%  Mixture: 3.6% |
| Kuhlmann et al. ^9^ | Omicron | NR | NR | NR | NR | 3 doses | NR | Mild: 57.14%  Moderate: 42.86% | General conclusions:  The mild to moderate course of illness suggests that full vaccination followed by a booster dose still provides good protection against severe disease caused by omicron.  Definitions:  Moderate was defined as shortness of breath.  Vaccine types:  Pfizer: 71.43% (5/7)  2 doses Pfizer, 1/2 dose Moderna: 14.29% (1/7)  1 dose Astrazeneca, 2 doses Pfizer: 14.29% (1/7) |
| Lauring et al. ^10^ | Alpha | Tobacco use: 11.6% *  CVD: 54.9% *  Chronic pulmonary disease: 21.5% *  Diabetes mellitus: 29.5% *  IC: 16.0% *  Obesity: 58.3% * | NR | Admission: 100%  ICU: 37.39%  MI: 2.01%  IMV: 21.3%  NIV: 17.7%  HFOT: 34.3%  Stroke: 1.91%  VTEE: 5.83%  NRRT: 4.56%  Vasopressors: 20.23%  Death: 8.05% | Tobacco use: 11.6% *  CVD: 54.9% *  Chronic pulmonary disease: 21.5% *  Diabetes mellitus: 29.5% *  IC: 16.0% *  Obesity: 58.3% * | 2 doses or Pfizer, Moderna, or mixed | NR | Admission: 100%  ICU: 20.69%  MI: 0.86%  IMV: 6.0%  NIV: 12.9%  HFOT: 12.9%  Stroke: 0%  VTEE: 6.03%  NRRT: 5.17%  Vasopressors: 4.31%  Death: 4.31% | Notes:  The comorbidities are based on hospitalized patients during the Alpha period, whether they had CoVid or not. Severity was specifically for those with CoVid.  Vaccnie types:  Pfizer: 68.1%  Moderna: 31.1%  Mixed: 0.8%  Statistical significance (Alpha vaccinated vs unvaccinated):  ICU: p<0.001  MI: p=0.39  IMV: p<0.001  NIV: p=0.20  HFOT: p<0.001  Stroke: p=0.13  VTEE: p=0.93  NRRT: p=0.91  Vasopressors: p<0.001  Death: p=0.15 |
| - | Delta | Tobacco use: 10.6% *  Chronic cardiovascular disease: 54.2% *  Chronic pulmonary disease: 20.9% *  Diabetes mellitus: 28.7% *  IC: 16.7% *  Obesity: 53.7% * | NR | Admission: 100%  ICU: 47.69%  IMV: 24.8%  NIV: 17.1%  HFOT: 41.9%  MI: 2.12%  Stroke: 1.60%  Thromboembolic event: 9.11%  NRRT: 5.80%  Vasopressors: 23.59%  Death: 11.78% | Tobacco use: 10.6% *  Chronic cardiovascular disease: 54.2% *  Chronic pulmonary disease: 20.9% *  Diabetes mellitus: 28.7% *  IC: 16.7% *  Obesity: 53.7% * | 2-3 doses of Pfizer, Moderna, or mixed | NR | Admission: 100%  ICU: 30.72%  IMV: 14.5%  NIV: 14.4%  HFOT: 27.7%  MI: 2.78%  Stroke: 0.86%  Thromboembolic event: 4.40%  NRRT: 4.69%  Vasopressors: 14.83%  Death: 13.21% | Notes:  The comorbidities are based on hospitalized patients during the Delta period, whether they had CoVid or not. Severity was specifically for those with CoVid.  Vaccine types:  Pfizer: 65.56%  Modera: 34.07%  Mixed: 0.37%  Statistical significance (Delta vaccinated vs unvaccinated):  ICU: p<0.001  MI: p=0.23  IMV: p<0.001  NIV: p=0.046  HFOT: p<0.001  Stroke: p=0.08  VTEE: p<0.001  NRRT: p=0.18  Vasopressors: p<0.001  Death: p=0.23 |
| - | Omicron | Tobacco use: 12.6% *  Chronic cardiovascular disease: 64.2% *  Chronic pulmonary disease: 27.0% *  Diabetes mellitus: 29.3% *  IC: 24.7% *  Obesity: 46.8% * | NR | Admission: 100%  ICU: 32.84%  IMV: 18.0%  NIV: 15.8%  HFOT: 30.9%  MI: 1.84%  Stroke: 1.47%  Thromboembolic event: 8.09%  NRRT: 4.41%  Vasopressors: 16.91%  Death: 9.19% | Tobacco use: 12.6% *  Chronic cardiovascular disease: 64.2% *  Chronic pulmonary disease: 27.0% *  Diabetes mellitus: 29.3% *  IC: 24.7% *  Obesity: 46.8% * | 2-3 doses of Pfizer, Moderna, or mixed | NR | Admission: 100%  ICU: 22.53%  IMV: 11.9%  NIV: 13.3%  HFOT: 20.1%  MI: 1.71%  Stroke: 1.02%  Thromboembolic event: 5.12%  NRRT: 4.78%  Vasopressors: 12.29%  Death: 5.12% | General conclusions:  Severity between variants was compared with adjusted proportional odds ratios (aPOR) using WHO Clinical Progression Scale. Among unvaccinated cases, COVID-19 severity on the WHO Clinical Progression Scale was highest for the Delta group  (Delta vs Alpha aPOR 1.28, 95% CI: 1.11 to 1.46) and lowest for the Omicron group (Omicron vs Alpha aPOR 0.79, 95% CI: 0.62 to 1.01; Omicron vs Delta aPOR 0.61, 95% CI: 0.49 to 0.77)  Vaccine types:  Pfizer: 69.76%  Moderna: 28.87%  Mixed: 1.37%  Statistical significance (Omicron vaccinated vs unvaccinated):  ICU: p=0.006  MI: p=0.91  IMV: p=0.0043  NIV: p=0.40  HFOT: p=0.003  Stroke: p=0.63  VTEE: p=0.15  NRRT: p=0.84  Vasopressors: p=0.12  Death: p=0.059 |
| Lee et al. ^11^ | Omicron | Cancer: 100% | NR | ASY: 28.07% *  ER/outpatient: 84.0% *  Admission: 39.3% *  Death: 3.57% | Cancer: 100% | 2 or 3 doses of Pfizer or Moderna OR 1 dose of J&J | NR | ASY: 28.07% *  ER/outpatient: 84.0% *  Admission: 39.3% *  Death: 5.85% | Notes:  There were significantly increased odds of inpatient admission for unvaccinated cancer patients (OR 2.01 [95% CI: 1.10–3.67, p = 0.022]). There was also no difference in mortality between vaccinated and unvaccinated cancer patients.  Admissions and asymptomatic cases were not stratified based on vaccination status but were reported for the cohort as a whole. |
| - | Omicron | - | NR | - | Cancer: 100% | 1 dose of Pfizer or Moderna | NR | ASY: 28.07% *  ER/outpatient: 84.0% *  Admission: 39.3% *  Death: 11.1% |  |
| Veneti et al. ^12^ | Omicron | Medium-risk comorbidities: 8.9% *  High-risk comorbidity: 1.2% * | 4.1% | Admission: 0.186%  ICU: 0.02% *  Death: 0.03% * | Medium-risk comorbidities: 8.9% *  High-risk comorbidity: 1.2% * | 1 dose | NR | Admission: 0.16%  ICU: 0.02% *  Death: 0.03% * | Notes/General conclusions:  Seven Omicron patients were admitted to an ICU. Ten (seven non-hospitalised) deaths were reported among the 39,524 Omicron cases. Nine Omicron deaths were reported as COVID-19 related.  None of the unvaccinated patients who were infected previously required hospitalization, suggesting a lower risk when compared to only unvaccinated with no prior infection.  Definitions:  Medium risk comorbidities  include chronic liver disease or significant hepatic impairment, immunosuppressive therapy as in autoimmune diseases, diabetes, chronic lung disease including cystic fibrosis and severe asthma which have required the use  of high dose inhaled or oral steroids within the past year, obesity, dementia, chronic heart and vascular disease (with the exception of HTN) and stroke.  High risk comorbidities include having received an organ transplant, immunodeficiency, hematological cancer in the last five years, other active cancers, ongoing or recently discontinued treatment for cancer (especially immunosuppressive therapy, radiation therapy to the lungs or cytotoxic drugs), neurological or  neuromuscular diseases that cause impaired cough or lung function (e.g., ALS and cerebral palsy), Down syndrome and chronic kidney disease, or significant renal impairment. |
| - | Omicron | - | - | - | Medium-risk comorbidities: 8.9% *  High-risk comorbidity: 1.2% * | 2 doses | NR | Admisssion: 0.22%  ICU: 0.02% *  Death: 0.03% * |  |
| - | Omicron | - | - | - | Medium-risk comorbidities: 8.9% *  High-risk comorbidity: 1.2% * | 3 doses | NR | Admission: 0.87%  ICU: 0.02% *  Death: 0.03% * |  |
| - | Delta | Medium-risk comorbidities: 10% *  High-risk comorbidity: 1.6% * | 0.34% | Admission: 1.46%  ICU: 0.26% *  Death: 0.18% * | Medium-risk comorbidities: 10% *  High-risk comorbidity: 1.6% * | 1 dose | NR | Admission: 0.40%  ICU: 0.26% *  Death: 0.18% * | Notes:  92 (30 non-hospitalised) deaths were reported among the Delta cases. |
| - | Delta | - | - | - | Medium-risk comorbidities: 10% *  High-risk comorbidity: 1.6% * | 2 doses | NR | Admission: 0.64%  ICU: 0.26% *  Death: 0.18% * |  |
| - | Delta | - | - | - | Medium-risk comorbidities: 10% *  High-risk comorbidity: 1.6% * | 3 doses | NR | Admission: 3.65%  ICU: 0.26% *  Death: 0.18% * |  |
| Vieillard-Baron et al. ^13^ | Omicron | IC: 34.5% * | NR | Pneumonia: 80.7%  ICU: NR  IMV: 41.0% *  Death: 20.0% * | IC: 34.5% * | 1-3 doses of Pfizer, Moderna, or AstraZeneca | NR | Pneumonia: 62.1%  ICU: 67.2%  IMV: 41.0% *  Death: 20.0% * | Statistical significance (Omicron vaccinated and unvaccinated):  Pneumonia: p<0.001  Vaccination status (for vaccinated patients):  1 dose: 13  2 doses: 59  3 doses: 60 |
| - | Delta | IC: 14.8% * | NR | Pneumonia: 97.8%  ICU: NR  IMV: 51.0% *  Death: 27.9%* | IC: 14.8% * | 1-3 doses of Pfizer, Moderna, or AstraZeneca | NR | Pneumonia: 87.5%  ICU: 94.8%  IMV: 51.0% *  Death: 27.9% * | Statistical significance (Delta vaccinated and unvaccinated):  Pneumonia: p<0.001  Statistical signficance (Omicron and Delta):  ICU: p<0.001  IMV: p=0.02  Death: p=0.082  Vaccination status (for vaccinated patients):  1 dose: 4290  2 doses: 22771  3 doses: 1505 |
| Wang et al. ^14^ | Omicron | **After matching:**  Cancer: 1.7%  Congenital heart diseases: 1.8%  Asthma: 1.7%  Blood disorders: 2.4%  Influenza and pneumonia: 3.4%  Autism: 0.3%  Common cold: 2.2%  **Before matching:**  Cancer: 1.7%  Congenital heart diseases: 1.8%  Asthma: 1.7%  Blood disorders: 2.4%  Influenza and pneumonia: 3.4%  Autism: 0.3%  Common cold: 2.2% | 0% | **After matching:**  Admission: 1.04%  ED visits: 18.83%  ICU: 0.14%  MV: 0.33% | NR | NR | NR | NR |  |
| - | Delta | **After matching:**  Cancer: 1.5%  Congenital heart diseases: 1.6%  Asthma: 3.7%  Blood disorders: 2.3%  Influenza and pneumonia: 3.2%  Autism: 0.3%  Common cold: 1.8%  **Before matching:**  Cancer: 2.4%  Congenital heart diseases: 2.9%  T1D: 0.05%  T2D: 0.03%  Asthma: 3.7%  Blood disorders: 4.6%  Influenza and pneumonia: 5.0%  Autism: 0.7%  Common cold: 3.8% | 0% | **After matching:**  Admission: 3.14%  ED visits: 26.67%  ICU: 0.43%  IMV: 1.15% | NR | NR | NR | NR |  |
| Marks et al. ^15^ | Delta | NR | NR | ASY: 16.6%  SY: 83.4%  Admission: 100%  Admission related to CoVid: 70.8%  ICU: 31.6%  IMV: 7.1%  Death: 0.9% | NR | 2 doses | NR | ASY: 30.2%  SY: 69.8%  Admission: 100%  Admission related to CoVid: 35.8%  ICU: 15.1%  IMV: 9.4%  Death: 3.8% | General conclusions:  “Throughout the periods of Delta and Omicron predominance, hospitalization rates remained lower among fully vaccinated adolescents aged 12–17 years than among unvaccinated adolescents.” |
| - | Omicron | NR | NR | ASY: 21.0%  SY: 79.0%  Admission: 100%  Admission related to CoVid: 65.0%  ICU: 19.1%  IMV: 1.6%  Death: 0% | NR | 2 doses | NR | ASY: 11.1%  SY: 88.9%  Admission: 100%  Admission related to CoVid: 55.5%  ICU: 16.6%  IMV: 5.5%  Death: 0% | Statistical signficance (Unvaccniated and vaccinated):  SY: p=0.08  Admissions: p<0.001  ICU: p=0.009  IMV: p=0.54  Death: p=0.10 |
| Maisa et al. ^16^ | Omicron | NR | 14% | ASY: 11% *  SY: 89% *  Admission: 2% *  ICU: 0% *  Death: 0% * | NR | 1-3 doses | NR | ASY: 11% *  SY: 89% *  Admission: 2% *  ICU: 0% *  Death: 0% * |  |
| Maruki et al. ^17^ | Omicron | NR | NR | NR | NR | 2 doses Moderna | 0% | Mild: 100% | Notes:  This paper reports the first 2 cases of breakthrough COVID-19 caused by Omicron in Japan. |
| - | Omicron | NR | NR | NR | NR | 2 doses Pfizer | 0% | Mild: 100% |  |
| Loconsole et al. ^18^ | Omicron | NR | NR | NR | NR | 3 doses Pfizer | NR | ASY: 66.7%  Mild: 33.3% | General conclusions:  Omicron is highly transmissible and has a high capacity to cause outbreaks even among HCWs who have received the booster vaccination dose. |
| Micheli et al. ^19^ | Omicron | NR | NR | NR | NR | 2 doses Astrazeneca + 1 booster Pfizer | NR | Mild: 100% |  |
| Modes et al. ^20^ | Omicron | Obesity: 36.2% “  Kidney disease: 11.4% “  HTN: 12.4% “  CVD: 10.5% “  Diabetes: 5.7% “  COPD: 1.9% “ | NR | Admission: 100%  ICU: 19.0%  IMV: 10.8%  Death: 4.9% | Obesity: 36.2% “  Kidney disease: 11.4% “  HTN: 12.4% “  CVD: 10.5% “  Diabetes: 5.7% “  COPD: 1.9% “ | 2-3 doses | NR | Admission: 100%  ICU: 15.3%  IMV: 8.6%  Death: 3.4% | Notes:  Comorbidities were reported for hospitalizations specifically.  Admissions includes ICU, IMV, and death.  Denominator for ICU calculations excludes 70 fully vaccinated patients who received a booster dose.  Statistical significance (Omicron and Delta):  ICU: p=0.01  IMV: p=0.03  Death: p=0.01 |
| - | Delta | Obesity: 36.2% “  Kidney disease: 11.4% “  HTN: 12.4% “  CVD: 10.5% “  Diabetes: 5.7% “  COPD: 1.9% “ | NR | Admission: 100%  ICU: 22.8%  IMV: 15.4%  Death: 7.9% | Obesity: 36.2% “  Kidney disease: 11.4% “  HTN: 12.4% “  CVD: 10.5% “  Diabetes: 5.7% “  COPD: 1.9% “ | 2-3 doses | NR | Admission: 100%  ICU: 23.5%  IMV: 9.4%  Death: 4.9% | Statistical significance (unvaccinated Omicron and Delta):  ICU: p=0.27  IMV: p=0.11  Death: p=0.21  Statistical significance (vaccinated Omicron and Delta):  ICU: p=0.10  IMV: p=0.82  Death: p=0.02 |
| Robinson et al. ^21^ | Ancestral | IC: 8.2%  Diabetes: 41.3%  CVD: 49.6%  COPD: 33.6%  Malignancy: 30.4%  Pregnancy: 0.6% | 0% | Admission: 100%  Severe/Death: 1.98% | IC: 20.0%  Diabetes: 60.0%  CVD: 60.0%  COPD: 60.0%  Malignancy: 20.0%  Pregnancy: 0.0% | 1 dose J&J or 2 doses Pfizer or Moderna | 60.0% | Admission: 100%  Severe/Death: 0.0% |  |
| - | Alpha | IC: 17.8%  Diabetes: 43.0%  CVD: 50.5%  COPD: 36.4%  Malignancy: 34.1%  Pregnancy: 0.9% | 0% | Admission: 100%  Severe/Death: 30.84% | IC: 11.1%  Diabetes: 44.4%  CVD: 55.6%  COPD: 44.4%  Malignancy: 44.4% | 1 dose Astrazeneca or 2 doses Pfizer or Moderna | 11.11% | Admission: 100%  Severe/Death: 22.22% | Sample calculations:  Unvaccinated → Severe/Death = 66/214=30.84%  Vaccinated →Severe/Death = 2/9=22.22%  Notes:  Study reported the inferred and confirmed variants; only the number of confirmed cases were used for calculations |
| - | Delta | IC: 7.9%  Diabetes: 33.5%  CVD: 37.1%  COPD: 29.4%  Malignancy: 20.4%  Pregnancy: 1.2% | 0% | Admission: 100%  Severe/Death: 20.77% | IC: 25.1%  Diabetes: 49.8%  CVD: 61.9%  COPD: 39.9%  Malignancy: 47.9% | 1 dose Astrazeneca or 2 doses Pfizer or Moderna | 0.7% | Admission: 100%  Severe/Death: 17.18% | Sample calculations:  Unvaccinated →Severe/Death = 118/568=20.77%  Vaccinated →Severe/Death = 50/291=%  Notes:  Study reported the inferred and confirmed variants; only the number of confirmed cases were used for calculations |
| - | Omicron | IC: 6.9%  Diabetes: 38.1%  CVD: 49.2%  COPD: 29.3%  Malignancy: 24.1%  Pregnancy: 0.9% | 0% | Admission: 100%  Severe/Death: 9.35% | IC: 20.4%  Diabetes: 48.4%  CVD: 66.2%  COPD: 39.9%  Malignancy: 38.6% | 1 dose Astrazeneca or 2 doses Pfizer or Moderna | 0.2% | Admission: 100%  Severe/Death: 4.00% | Sample calculations:  Unvaccinated →Severe/Death = 20/214 = 9.35%  Vaccinated →Severe/Death = 20/500=%  Notes:  Study reported the inferred and confirmed variants; only the number of confirmed cases were used for calculations |
| - | Other variants | IC: 5.9%  Diabetes: 38.2%  CVD: 38.2%  COPD: 35.3%  Malignancy: 35.3%  Pregnancy: 0.0% | 0% | Admission: 100%  Severe/Death: 26.5% | IC: 33.3%  Diabetes: 0.0%  CVD: 66.7%  COPD: 33.3%  Malignancy: 33.3%  Pregnancy: 0.0% | 1 dose J&J or 2 doses Pfizer or Moderna | 33.3% | Admission: 100%  Severe/Death: 0.0% |  |
| Ludvigsson et al. ^22^ | Omicron | NR | 0% | Mild: 50%  ICU: 50%  Convulsions: 100% | UTI: 100% | 1 dose Pfizer | 0% | Mild: 100%  Convulsions: 100% | Notes:  Repeated UTIs in vaccinated child were in the past. No current complains of infections. Unvaccinated children had no comorbidities. |
| Piersiala et al. ^23^ | Omicron | Hepatitis B: 25% | 0% | Odynophagia: 100%  Sore throat: 100%  Hoarsness: 25%  Fever: 50% | NR | 1 dose | 0% | Odynophagia: 100%  Sore throat: 100%  Hoarsness: 100%  Fever: 100% |  |
| - | Omicron | - | NR | - | Bipolar disorder: 7.69% | 2 doses | 7.69% | Odynophagia: 100%  Sore throat: 100%  Hoarsness: 30.77%  Fever: 84.62% |  |
| - | Omicron | - | NR | - | Depression: 50% | 3 doses | 0% | Odynophagia: 100%  Sore throat: 100%  Hoarsness: 50%  Fever: 50% |  |
| Tseng et al. ^24^ | Delta | NR | NR | NR | Smoker: 16.5%  Kidney disease: 1.4%  Heart disease: 1.2%  Lung disease: 6.6%  Liver disease: 2.3%  Diabetes: 5.8%  IC: 1.1%  Autoimmune: 1.9%  Pregnancy: 1.9% | 1 dose Moderna | 2.7% | Outpatient/virtual visits: 84.4%  ED visits: 14.6%  Admission: 4.9% | Notes:  There were some cases where smoking status was unknown.  There were subclassifications of autoimmune diseases and immunocompromising diseases mentioned in the tables. Refer back to the table for more information. |
| - | Delta | NR | NR | NR | Smoker: 16.3%  Kidney disease: 1.9%  Heart disease: 1.3%  Lung disease: 6.9%  Liver disease: 2.7%  Diabetes: 7.5%  IC: 1.6%  Autoimmune: 2.3%  Pregnancy: 1.7% | 2 doses Moderna | 2.5% | Outpatient/virtual visits: 87.8%  ED visits: 15.0%  Admission: 4.7% |  |
| - | Delta | NR | NR | NR | Smoker: 16.9%  Kidney disease: 1.9%  Heart disease: 1.4%  Lung disease: 6.8%  Liver disease: 2.4%  Diabetes: 6.3%  IC: 1.5%  Autoimmune: 2.2%  Pregnancy: 1.9% | 3 doses Moderna | 3.0% | Outpatient/virtual visits: 85%  ED visits: 14.6%  Admission: 1.3% |  |
| - | Omicron | NR | NR | NR | Smoker: 14.2%  Kidney disease: 0.7%  Heart disease: 0.7%  Lung disease: 5.9%  Liver disease: 2.0%  Diabetes: 4.4%  IC: 1.5%  Autoimmune: 1.6%  Pregnancy: 2.2% | 1 dose Moderna | 18.1% | Outpatient/virtual visits: 86.6%  ED visits: 14.7%  Admission: 6.6% |  |
| - | Omicron | NR | NR | NR | Smoker: 14.0%  Kidney disease: 1.1%  Heart disease: 0.8%  Lung disease: 6.3%  Liver disease: 2.4%  Diabetes: 6.8%  IC: 1.7%  Autoimmune: 1.8%  Pregnancy: 1.8% | 2 doses Moderna | 13.6% | Outpatient/virtual visits: 91.6%  ED visits: 15.6%  Admission: 33.3% |  |
| - | Omicron | NR | NR | NR | Smoker: 14.7%  Kidney disease: 2.0%  Heart disease: 1.2%  Lung disease: 6.9%  Liver disease: 2.4%  Diabetes: 7.4%  IC: 2.4%  Autoimmune: 2.3%  Pregnancy: 2.0% | 3 doses Moderna | 15.4% | Outpatient/virtual visits: 89.3%  ED visits: 16.6%  Admission: 22.2% |  |
| Thompson et al. ^25^ | Delta | Chronic respiratory condition: 63.9% “  Chronic non-respiratory condition: 76.4% “ | NR | Admission: 39.1% | Chronic respiratory condition: 63.9% “  Chronic non-respiratory condition: 76.4% “ | 2 doses | NR | Admission: 34.0% | Definitions:  Chronic nonrespiratory condition included heart failure, ischemic heart disease, hypertension, other heart disease, stroke, other cerebrovascular disease, diabetes, metabolic disease, clinical obesity, clinically underweight, renal disease, liver disease, blood disorder, immunosuppression, organ transplant, cancer, dementia, neurologic disorder, musculoskeletal disorder, or Down syndrome. |
| - | Delta | Chronic respiratory condition: 63.9% “  Chronic non-respiratory condition: 76.4% “ | NR | - | Chronic respiratory condition: 63.9% “  Chronic non-respiratory condition: 76.4% “ | 3 doses | NR | Admission: 44.6% |  |
| - | Omicron | Chronic respiratory condition: 63.9% “  Chronic non-respiratory condition: 76.4% “ | NR | Admission: 5.12% | Chronic respiratory condition: 63.9% “  Chronic non-respiratory condition: 76.4% “ | 2 doses | NR | Admission: 3.81% |  |
| - | Omicron | Chronic respiratory condition: 63.9% “  Chronic non-respiratory condition: 76.4% “ | NR | - | Chronic respiratory condition: 63.9% “  Chronic non-respiratory condition: 76.4% “ | 3 doses | NR | Admission: 4.62% |  |
| Cloete et al. ^26^ | Omicron | Haematological or oncological disease: 6%  Type 1 diabetes: 5%  Cardiac disease: 4%  HIV: 4%  Cerebral palsy: 2%  Asthma: 1%  Other: 34% | NR | Mild: 79%  Oxygen therapy: 20%  Ventilation: 5%  HFOT: 1%  Nasal prong oxygen: 14%  Death: 3% | NR | NR | NR | NR | Notes:  Other comorbidities included Included neonatal jaundice and sepsis, epilepsy, tuberculosis, neurosurgical conditions, burn wounds, and other paediatric surgical and orthopaedic conditions. Some children’s data was missing comorbidities. |
| Goussard et al. ^27^ | Omicron | Prematurity | 0% | Apnea: 100%  Respiratory failure: 100%  Death: 100% | NR | NR | NR | NR |  |
| Ferdinands et al. ^28^ | Delta | Chronic respiratory condition: 16.13%  Chronic nonrespiratory condition: 22.44%  IC: 3.02% | NR | Mild/Moderate: 100%  Admission: 49.7% | Chronic respiratory condition: 18.40%  Chronic nonrespiratory condition: 27.76%  IC: 4.24% | 2 doses | NR | Mild/Moderate: 100%  Admission: 40.7% | Vaccine type (2 doses):  Pfizer: 60.76%  Moderna: 39.02%  Combination: 0.22%  Notes:  Comorbidities were stratified based on number of doses and was reported for both COVID-19 positive and negative patients. The paper also reported numbers for positive alone but it was not stratified based on variant or doses.  Comorbidities (overall):  Chronic respiratory condition: 11.75%  Chronic nonrespiratory condition: 19.23%  IC: 2.14% |
| - | Delta | - | NR | - | Chronic respiratory condition: 21.04%  Chronic nonrespiratory condition: 32.25%  IC: 6.91% | 3 doses | NR | Mild/Moderate: 100%  Admission: 56.2% | Vaccine type (3 doses):  Pfizer: 63.23%  Moderna: 31.60%  Combination: 5.17% |
| - | Omicron | Chronic respiratory condition: 16.13%  Chronic nonrespiratory condition: 22.44%  IC: 3.02% | NR | Mild/Moderate: 100%  Admission: 13.5% | Chronic respiratory condition: 18.40%  Chronic nonrespiratory condition: 27.76%  IC: 4.24% | 2 doses | NR | Mild/Moderate: 100%  Admission: 11.7% |  |
| - | Omicron | - | NR | - | Chronic respiratory condition: 21.04%  Chronic nonrespiratory condition: 32.25%  IC: 6.91% | 3 doses | NR | Mild/Moderate: 100%  Admission: 14.2% |  |
| Gray 2021 et al. ^29^ | Omicron | CDC Risk Factor 1: 20.1% *  CDC Risk Factor 2: 4.9% *  CDC Risk Factor 3+: 1.2% * | Ancestral: 4.1% *  Beta: 3.9% *  Delta: 5.7% * | Admission: 3.9% | CDC Risk Factor 1: 20.1% *  CDC Risk Factor 2: 4.9% *  CDC Risk Factor 3+: 1.2% * | 2 doses J&J | Ancestral: 4.1% *  Beta: 3.9% *  Delta: 5.7% * | Admission: 1.2% | Definitions:  CDC Risk Factor is based on the presence of diseases including cancer, CVD, renal disease, chronic respiratory disease, diabetes, HIV, HTN, liver disease, neurological disorders, obesity/overweight, mental disorders, and organ transplant. |
| Helmsdal et al. ^30^ | Omicron | NR | NR | NR | Any comorbidity: 19% | 3 doses Pfizer | NR | Fever: 62%  Headache: 52%  Dry cough: 62%  Wet cough: 38%  Dyspnea: 38%  Anosmia: 19%  Ageusia: 24%  Fatigue: 71%  Rhinorrhea: 57%  Sinusitis: 29%  Throat pain: 48%  Myalgia: 62%  Arthalgia: 52%  Chest pain: 19% | Notes:  The paper further classified the symptoms listed on the left as mild, moderate, and severe (ex. fever was reported as either mild, moderate, or severe). The values to the left are the overall presence of each of these symptoms, regardless of severity. |
| Ward et al. ^31^ | Delta | NR | NR | NR | 1-2 comorbidities: 11.1%  3 comorbidities: 0.4% | 1-3 doses of Pfizer, Moderna, or AstraZeneca | 1.0% | Death: 0.09% | Sample calculations:  1-2 comorbidities = (0.201 x 122586) / 221152 = 11.14%  Death = 189/221152 = 0.09%  Definitions:  Comorbidities included asthma, atrial fibrillation, cancer, kidney disease, COPD, diabetes, HF, stroke, etc.  Vaccine types:  UV; 32516  1 dose: 8404  2 doses AstraZeneca: 28307  2 doses Pfizer or Moderna: 130034  Booster: 20567 |
| - | Omicron | NR | NR | NR | 1-2 comorbidities: 12.0%  3 comorbidities: 0.4% | 1-3 doses of Pfizer, Moderna, or AstraZeneca | 6.6% | Death: 0.02% | Vaccine types:  UV: 78958  1 dose: ​​24420  2 doses AstraZeneca: 72446  2 doses Pfizer or Moderna: 425724  Booster: 212455 |
| Sami et al. ^32^ | Omicron | NR | NR | NR | NR | 1-3 doses of Pfizer, Moderna, or 1 dose J&J | NR | Admission: 0.84%  Death: 0% | Notes:  These results were based on a surveillance system after event-associated infections.  Vaccine types:  Partially vaccinated: 3  2 doses of Pfizer or Moderna or 1 dose J&J: 80  3 doses: 5 |
| - | Omicron | NR | NR | NR | NR | 1-3 doses of Pfizer, Moderna, or 1 dose J&J | NR | Mild: 70.8%  Admission: 0%  Death: 0% | Mild symptoms were reported as “COVID-19 compatible symptoms” which include nasal congestion or runny nose (91.2%) and fatigue (88.2%).  These results were based on an online survey.  Vaccine types:  Partially vaccinated: 4  2 doses of Pfizer or Moderna or 1 dose J&J: 37  3 doses: 6 |
| Patalon et al. ^33^ | Omicron | NR | NR | NR | NR | 3 doses Pfizer | NR | Admission: 0.21%  Death: 0.01% |  |
| - | Omicron | NR | NR | NR | NR | 2 doses Pfizer | NR | Admission: 0.72%  Death: 0% |  |

Comorbidity abbreviations: AF: Atrial fibrillation, CAD: Coronary artery disease, CeVD: Cerebrovascular disease, CLD: Chronic lung disease, COPD: Chronic obstructive pulmonary disease, CVD: Cardiovascular disease, HF: Heart failure, HIV: Human immunodeficiency virus, HT: Hypothyroidism, HTN: Hypertension, IC: Immunocompromised/Immunosuppressed, TB: Tuberculosis.

Severity abbreviations: ASY: Asymptomatic, ECMO: Extracorporeal membrane oxygenation, ED: Emergency departments, HFOT: High flow oxygen therapy, ICU: Intensive care unit, IMV: Invasive mechanical ventilation, LFOT: Low flow oxygen therapy, MI: Myocardial infarction, MV: Mechanical ventilation, NIV: Noninvasive ventilation, NRRT: New renal replacement therapy, SY: Symptomatic, VTEE: Venous thromboembolic events.

General abbreviations: HCW: Healthcare workers, NR: Not reported.

* Not stratified based on vaccination status

“ Not stratified based on vaccination status or COVID-19 variant

- Data is same as data in row above
