# Supplementary material for: Severity of the Omicron SARS‐CoV‐2 variant compared with the previous lineages: A systematic review
Source: J Cell Mol Med. 2023 May 18;27(11):1443–64. doi: 10.1111/jcmm.17747 (PMC10243162; doi:10.1111/jcmm.17747)
Supplement: Supplementary file 3 — TABLE S3 Demographic and clinical data from studies that did not report vaccination status in cases infected with Omicron or other variants. [file JCMM-27-1443-s004.docx]

**Supplementary Table 3: Demographic and clinical data from studies that did not report vaccination status in cases infected with Omicron or other variants**

| **Author(s) and study**  **Source of data**  **Type of population** | **Type of study and country** | **Variant** | **N (number of subjects) and gender (XF/YM)** | **Age mean±SD or median (IQR)** | **Comorbidities**  **% and name of disease)** | **Previously infected %** | **Severity %** | **Notes / conclusion made by this study** |
| --- | --- | --- | --- | --- | --- | --- | --- | --- |
| Ebell et al. ^34^  Lehigh Valley Hospital, Inc. (LVH)  General population | Observational cohort (United States) | NR | 9649 | 41.9 (non- hospitalized) and 60.2 (hospitalized) | Diabetes: 9.82%  Asthma: 10.04%  COPD or chronic bronchitis: 2.69%  HTN: 22.87%  CVD: 4.60%  Kidney disease: 2.13%  Liver disease: 4.16%  Cancer: 4.03% | NR | Dyspnea: 10.32%  Admission: 4.18% | Notes:  No variant was specifically mentioned, however, the data was collected between March 2020- September 2021.  Age was reported as the mean and ranges were stratified based on hospitalization status.  Calculations for comorbidities and symptom severity were the combination of both hospitalized & non-hospitalized patients.  Overall age categories (hospitalized and non-hospitalized)  <50: 6051  50-59: 1715  60-69: 1132  70+: 751  Statistical significance:  The p-value for all comorbidities except asthma when comparing hospitalized to non-hospitalized patients was < 0.001. |
| Dinh et al. ^35^  Covidom, a telesurveillance solution for home monitoring of patients with COVID-19  General population | Cohort study (France) | Alpha | 88666 (54061/34605) | **First surge:** 44.2±14.5  **Second surge:**  42.0±14.3 | HTN: 5.53%  Diabetes: 2.39%  IC: 0.22%  Chronic pulmonary disease: 6.54%  Chronic cardiac failure: 1.20%  Active neoplasia: 0.79% | NR | Respiratory symptoms: 58.50%  Digestive symptoms: 33.96%  Systemic symptoms: 58.44%  Admission: 0.19%  ICU: 0.10% | Age was reported as ranges and means for each surge separately.  <40: 36874  40-60: 34005  60-70: 7673  70-75: 6479  >75: 3635  Notes:  The Alpha wave happened in two surges: Mar 2020-May 2020 and Sept 2020-Nov 2020. The number of patients was added for both surges. ICU was labeled as an “emergency unit” in the paper. |
| - | - | Delta | 64188 (36395/27793) | **First surge:**  40.7±14.1  **Second surge:**  35.6±13.0 | HTN: 3.08%  Diabetes: 1.62%  IC: 0.09%  Chronic pulmonary disease: 3.08%  Chronic cardiac failure: 0.29%  Active neoplasia: 0.41% | NR | Respiratory symptoms: 62.39%  Digestive symptoms: 63.67%  Systemic symptoms: 64.14%  Admission: 0.46%  ICU: 0.07% | Age was reported as ranges and means for each surge separately.  <40: 33669  40-60: 23273  60-70: 4013  70-75: 1818  >75: 1415  Notes:  The Delta wave happened in two surges: Mar 2021-May 2021 and July 2021-Sept 2021. The number of patients was added for both surges. |
| - | - | Omicron | 72394 (42279/30115) | 38.4±13.0 | HTN: 1.1%  Diabetes: 0.9%  IC: 0.1%  Chronic pulmonary disease: 1.6%  Chronic cardiac failure: 0.1%  Active neoplasia: 0.2% | NR | Respiratory symptoms: 49.8%  Digestive symptoms: 45.1%  Systemic symptoms: 51.8%  Admission: 0.06%  ICU: 0.03% | Age was reported as ranges and means for each surge separately.  <40: 38157  40-60: 25353  60-70: 3325  70-75: 4569  >75: 990  Statistical significance (Omicron and Alpha):  Respiratory symptoms: p<0.001  Statistical significance (Omicron and previous waves):  Admissions: p<0.0001  ICU: p<0.0001  Vaccination status (only reported for Omicron):  Unvaccinated: 3625  1 dose: 421  2 doses: 9950  3 doses: 1380 |
| Abdullah et al. ^36^  National Institute for Communicable Diseases, the National Department of Health and the South African Medical Research Council  Hospitalized patients | Cohort study (South Africa) | Omicron | 466 | 39±22.4 | NR | NR | Admission: 100%  ICU: 1%  Death: 4.5%  CoVid pneumonia: 36.7%  Oxygenation: 31.63%  MV: 4.08%  NIV: 4.08% | Notes:  Admission includes ICU and deaths.  Cause of death in 10 of the 21 patients was due to CoVid pneumonia, another cause exacerbated by CoVid pneumonia in 4, and 7 died of causes unrelated to CoVid.  Data about CoVid pneumonia, oxygenation, MV, and NIV are specific to the hospitalized patients only (98/466). |
| - | - | Ancestral, Beta, and Delta | 3962 | 49.8±21.8 | NR | NR | Admission: 100%  ICU: 4.3%  Death: 21.3% | Statistical significance (Omicron and Delta):  ICU: p=0.0007  Death: p<0.00001 |
| Wolter et al. ^37^  National Institute for Communicable Diseases (NICD)  Notifiable Medical Conditions Surveillance System (NMCSS)    National Health Laboratory Service (NHLS)  DATCOV    Normal population | Retro- spective Cohort Study    South Africa | Omicron BA1 | 87,194 (52,547/ 42,017) “ | NR | NR | 9.8% “ | Admission: 3.4% | Reported age as ranges for full cohort:  <5: 1681  5-12: 4426  13-18: 5278  19-24: 7127  25-39: 35551  40-59: 30953  >60: 10454  Notes:  Calculations were based on Supplementary Table 1.  Sample calculations:  Previously infected= 9384/ 95470 = 9.8%  Admission = 2,965/87,194  = 3.4% |
| - | - | Omicron BA2 | 8,276  (52,547/ 42,017) “ | NR | NR | 9.8% “ | Admission: 3.6% | Sample calculations:  Admission = 295/8,276  = 3.6% |
| Wang et al. ^38^    TriNetX Analytics    Normal population | Matched, retros- pective Cohort Study  (United States) | Omicron | **After matching:**  14,040)  (7,778/6,248)  14 Unknown  **Before matching:**  14,054  (7,786/6,268) | **After matching:**  36.4±24.3  **Before matching:**  36.4±24.3 | **After matching:**  HTN: 14.6%  Heart diseases: 3.7%  CeVD: 2.2%  Obesity: 10.6%  T2D: 5.6%  Cancers: 11.4%  Chronic respiratory diseases: 10.7%  Liver diseases: 2.1%  Chronic kidney disease: 2.2%  Blood disorders involving immune mechanisms: 10.2%  HIV: 0.16%  Dementia: 0.3%  Substance use disorders: 7.7%  Depression: 6.7%  Anxiety: 12.1%  Smoking: 1.9%  Alcohol abuse: 0.9%  Organ Transplant: 0.4%  **Before matching:**  HTN: 14.6%  Heart diseases: 3.7%  CeVD: 2.2%  Obesity: 10.6%  T2D: 5.6%  Cancer: 11.4%  Chronic respiratory diseases: 10.7%  Liver diseases: 2.1%  Chronic kidney disease: 2.2%  Blood disorders involving immune mechanisms: 10.3%  HIV: 0.16%  Dementia: 0.3%  Substance use disorders: 7.7%  Depression: 6.6%  Anxiety: 12.1%  Smoking: 1.9%  Alcohol abuse: 0.9%  Organ Transplant: 0.4% | 0% | **After matching:**  ED visits: 4.55%  Admission: 1.75%  ICU: 0.26%  MV: 0.07% | **Omicron:** (Date 12/15/2021–12/24/2021)  Vaccination status:  Vaccinated: 3.57%  Patients took Pfizer, Moderna, or J&J.  Notes:  Severity outcomes were reported for the cohorts after matching.  Sample calculations (before matching):  Gender calculated by multiplying the paper reported percentages by the total:  F: 55.4% x 14054  M: 44.6% x 14054 |
| - | - | Delta | **After matching:** 14,040  (7,750/6,276)  **Before matching:**  563,884  (303,933/ 259,387) | **After matching:**  36.1±24.4  **Before matching:**  36.1±25.3 | **After matching:**  HTN: 13.7%  Heart diseases: 3.3%  CeVD: 2.1%  Obesity: 9.8%  T2D: 5.3%  Cancers: 10.3%  Chronic respiratory diseases: 10.2%  Liver diseases: 2%  Chronic kidney disease: 2.1%  Blood disorders involving immune mechanisms: 9.5%  HIV: 0.16%  Dementia: 0.3%  Substance use disorders: 7.5%  Depression: 6%  Anxiety: 11.5%  Smoking: 1.4%  Alcohol abuse: 0.8%  Organ Transplant: 0.3%  **Before matching:**  HTN: 17.8%  Heart diseases: 4.7%  CeVD: 3.4%  Obesity: 11.3%  T2D: 7.3%  Cancer: 13.6%  Chronic respiratory diseases: 13.1%  Liver diseases: 3.5%  Chronic kidney disease: 3.2%  Blood disorders involving immune mechanisms: 12.7%  HIV: 0.23%  Dementia: 0.5%  Substance use disorders: 9.3%  Depression: 8.6%  Anxiety: 13.9%  Smoking: 2.4%  Alcohol abuse: 1.4%  Organ Transplant: 0.5% | 0% | **After matching:**  ED visits: 15.22%  Admission: 3.95%  ICU: 0.78%  MV: 0.43% | Notes:  Delta: (Date 9/1/2021–11/15/2021)  Severity outcomes were reported for the cohorts after matching.  Vaccination status:  Vaccinated: 4.35%  Sample calculations (before matching):  Gender calculated by multiplying the paper reported percentages by the total:  F: 53.9% x 563884  M: 46% x 563884 |
| Wang et al. ^39^    TriNetX Analytics    General population | Retro- spective Cohort Study  (United States) | Omicron | **After matching:**  147,107  (83,410/ 63,550)  147 Unknown  **Before matching:**  147,964  (83,896/ 63,920)  148 Unkown | **After matching**  39.1± 23.4  **Before matching**  39.1±23.4 | **After matching:**  Hypertension: 17.8%  Heart diseases: 3.9%  Cerebrovascular diseases: 2.8%  Obesity: 11.5%  T2D: 6.9%  Cancer: 12.6%  Chronic respiratory diseases: 11.2%  Liver diseases: 2.9%  Chronic kidney disease: 2.9%  Blood disorders involving immune mechanisms: 11.1%  HIV: 0.17%  Dementia: 0.4%  Substance use disorders: 7.7%  Depression: 8%  Anxiety: 14%  Smoking: 2.3%  Alcohol abuse: 1%  Organ Transplant: 0.4%  **Before matching:**  Hypertension: 17.8%  Heart diseases: 3.9%  CeVD: 2.8%  Obesity: 11.5%  T2D: 6.9%  Cancer: 12.6%  Chronic respiratory diseases: 11.1%  Liver diseases: 2.9%  Chronic kidney disease: 2.9%  Blood disorders involving immune mechanisms: 11.1%  HIV: 0.17%  Dementia: 0.4%  Substance use disorders: 7.7%  Depression: 8%  Anxiety: 14%  Smoking: 2.3%  Alcohol abuse: 1%  Organ Transplant: 1% | 0% | **After matching:**  ED visits: 10.19%  Admission: 2.6%  ICU: 0.47%  MV: 0.08% | Notes:  Omicron**:** (Date 12/26/2021–1/16/2022)  148 patients had unkown gender.  Severity outcomes were reported for the cohorts after matching.  Vaccination status (before matching):  Vaccinated Omicron cases who are above 5 years old: 7.7%  Vaccinated Delta cases who are above 5 years old: 13.6%  Sample calculations (before matching):  Gender calculated by multiplying the paper reported percentages by the total:  F: 56.7% x 147964  M: 43.2% x 147964 |
| - | - | Delta | **After matching:**  147,107  (82,968/ 64,139)  14 unknown  **Before matching:**  633,581  (342,767/ 290,180)  634 Unknown | **After matching:**  38.9±23.9  **Before matching:**  36.4±25.2 | **After matching:**  HTN: 16.9%  Heart diseases: 3.7%  CeVD: 2.6%  Obesity: 10.7%  T2D: 6.3%  Cancer: 11.9%  Chronic respiratory diseases: 10.6%  Liver diseases: 2.6%  Chronic kidney disease: 2.6%  Blood disorders involving immune mechanisms: 10.4%  HIV infection: 0.16%  Dementia: 0.4%  Substance use disorders: 7.4%  Depression: 7.4%  Anxiety: 13%  Smoking: 2.1%  Alcohol abuse: 1%  Organ Transplant: 0.3%  **Before matching:**  HTN: 18.3%  Heart diseases: 4.9%  CeVD: 3.6%  Obesity: 11.5%  Type 2 diabetes: 7.6%  Cancers: 13.7%  Chronic respiratory diseases: 13.3%  Liver diseases: 3.7%  Chronic kidney disease: 3.4%  Blood disorders involving immune mechanisms: 13%  HIV: 0.23%  Dementia: 0.6%  Substance use disorders: 9.5%  Depression: 8.7%  Anxiety: 13.9%  Smoking: 2.4%  Alcohol abuse: 1.5%  Organ Transplant: 0.5% | 0% | **After matching:**  ED visits: 14.63%  Admission: 4.44%  ICU: 1%  MV: 0.3% | Notes:  Delta: (Date 9/1/2021–11/15/2021)  Severity outcomes were reported for the cohorts after matching.  Sample calculations (before matching):  Gender calculated by multiplying the paper reported percentages by the total:  F: 54.1% x 633581  M: 45.8% x 633581 |
| Wolter et al. ^56^    National Institute for Communicable Diseases (NICD),  Notifiable Medical Conditions Surveillance System (NMCSS), National Health Laboratory Service (NHLS)  DATCOV    Normal population | Retro- spective Cohort Study  (South Africa) | Omicron  (SGTF) | 10547 (6,375/5029) “ | NR | NR | 9.9% “ | Admission: 2.43% | Reported as age groups  <5: 156  5-12: 622  13-18: 669  19-24: 1115  25-39: 4969  40-59: 3156  >60: 808  Sample calculations:  SGTF Admission = 256/10547  = 2.43% |
| - | - | Unknown (Non- SGTF) | 948 (6,375/5029) “ | NR | NR | 9.9% “ | Admission: 12.76% | Notes:  Non-SGTF wasn’t clearly defined in terms of which variants it’s a proxy for  Sample calculations:  Non-SGTF Admission = 121/948  = 12.76% |
| Maslo et al. ^40^  Netcare Private Health Group  General population | Case- control study (South Africa) | Ancestral | 3875 (1337/1291) | 53 (21.75) | Patients with comorbidites: 56.0%  Include diabetes, heart conditions and hypertension,  chronic kidney failure, chronicpulmonary conditions, and cancer | NR | Treated in hospital: 100%  Acute respiratory condition: 72.6%  Admission: 67.8%  Oxygen therapy: 54.68%  MV: 11.12%  ICU: 28.49%  Deaths: 13.42% | Notes:  Paper does not mention whether there is overlap between the percentages for severity. |
| - | - | Beta | 4632 (1657/1541) | 54 (21) | Patients with comorbidites: 58.4%  Include diabetes, heart conditions and hypertension,  chronic kidney failure, chronic pulmonary conditions, and cancer | NR | Treated in hospital: 100%  Acute respiratory condition: 87.0%  Admission: 69.0%  Oxygen therapy: 56.65%  MV: 5.59%  ICU: 25.30%  Deaths: 17.06% |  |
| - | - | Delta | 6342 (2035/2365) | 59 (24) | Patients with comorbidites: 52.5%  Include diabetes, heart conditions and hypertension,  chronic kidney failure, chronicpulmonary conditions, and cancer | NR | Treated in hospital: 100%  Acute respiratory condition: 91.2%  Admission: 69.3%  Oxygen therapy: 51.40%  MV: 8.64%  ICU: 20.78%  Deaths: 20.25% |  |
| - | - | Omicron | 880 (590/381) | 36 (32) | Patients with comorbidities: 23.3%  Include diabetes, heart conditions and hypertension,  chronic kidney failure, chronic pulmonary conditions, and cancer | NR | Treated in hospital: 100%  Acute respiratory condition: 31.6%  Admission: 41.3%  Oxygen therapy: 7.27%  MV: 0.68%  ICU: 7.66%  Deaths: 1.15% | Vaccination status:  Unvaccinted: 645  2 doses Pfizer or 1 dose J&J: 235  Statistical significance (Delta and Omicron):  Admissions: p<0.001  Oxygen therapy: p<0.001  MV: p<0.001  ICU: p<0.001  Death: p<0.001 |
| Jassat et al. ^41^  Laboratory reports and DATCOV, an active surveillance programme established specifically for COVID-19  General population | Cohort study (South Africa) | D614G | 553530 | NR | Comorbid condition: 62.2% “ | NR | Admission: 12.72%  Severe: 6.65%  Supplemental oxygen: 4.68%  ICU: 2.01%  Death: 2.73% | Age was reported as ranges:  <5: 6280  5-19: 41829  20-39: 223169  40-59: 208211  >=60: 74041  Notes:  Vaccination status was mentioned per age group, but not well established for each variant.  Percent of comorbid conditions was reported specifically for severe cases in this study.  Percent admission includes patients who had severe disease, needed supplemental oxygen, and those treated in ICU in this study (these values were calculated from the total infected and were based on patients with known outcomes).  Repeat admissions were included in analysis.  Sample calculations:  ICU = 11125/553530 = 2.01% |
| - | - | Beta | 726772 | NR | Comorbid condition: 62.2% “ | NR | Admission: 12.43%  Severe: 7.88%  Supplemental oxygen: 5.95%  ICU: 1.60%  Death: 3.58% | Age was reported as ranges:  <5: 8522  5-19: 56873  20-39: 271440  40-59: 258981  >=60: 130956  Notes:  Admissions were based on hospitalized patients with known outcomes. |
| - | - | Delta | 1306260 | NR | Comorbid condition: 62.2% “ | NR | Admission: 10.0%  Severe: 6.20%  Supplemental oxygen: 4.69%  ICU: 1.44%  Death: 2.60% | Age was reported as ranges:  <5: 19019  5-19: 188689  20-39: 483252  40-59: 437093  >=60: 178207  Notes:  Admissions were based on hospitalized patients with known outcomes. |
| - | - | Omicron | 629617 | NR | Comorbid condition: 62.2% “ | NR | Admission: 7.29%  Severe: 2.45%  Supplemental oxygen: 1.68%  ICU: 0.46%  Death: 0.78% | Age was reported as ranges:  <5: 14050  5-19: 68778  20-39: 273902  40-59: 188486  >=60: 84401  Notes:  Admissions were based on hospitalized patients with known outcomes.  Statistical significance (D614G, Beta, Delta, and Omicron):  Admissions: p<0.001 |
| Iuliano et al. ^42^  National Syndromic Surveillance Program (NSSP), Unified  Hospital Data Surveillance System, and CDC using BD Insights Research Database (BD)  Hospitalized patients | Cohort study  (United States) | Omicron | 12,800 | NR | NR | NR | ICU: 13%  IMV: 3.5%  Death: 7.1% | Age was reported as ranges:  0-17: 405  18-50: 3988  50+: 8407  General conclusions:  Despite Omicron seeing the highest reported numbers of COVID-19 cases and hospitalizations during the pandemic, disease severity indicators, including length of stay, ICU admission, and death, were lower than during previous pandemic peaks.  Although disease severity appears lower with the Omicron variant, the high volume of hospitalizations can strain local health care systems and the average daily number of deaths remains substantial. |
| - | - | Delta | 10,440 | NR | NR | NR | ICU: 17.5%  IMV: 6.6%  Death: 12.3% | Age was reported as ranges:  0-17: 272  18-50: 3304  50+: 6864 |
| - | - | NR | 12,963 | NR | NR | NR | ICU: 18.2%  IMV: 7.5%  Death: 12.9% | Notes:  The data was reported as winter period from January 1, 2021 - January 21, 2021.  Age was reported as ranges:  0-17: 147  18-50: 2474  50+: 10342 |
| Martin et al. ^43^  US National COVID Cohort Collaborative (N3C)  Hospitalized UAI Pediatric Population | Retro-  spective Observa- tional Cohort Study  (United States) | Pre- Omicron | 206 (79/127) | 4.4±4.5 | Obese: <9.7%  Diabetes: <9.7%  Asthma: 19.42% | NR | Moderate: 63.6%  Severe: 38.8%  IMV: 34.0% | Definitions:  Severe includes patients who needed IMV, vasoactive inotropes, or extracorporeal membrane oxygenation support or who died, whereas moderate disease includes hospitalized children without any of these. |
| - | - | Omicron | 178 (53/125) | 2.1±2.1 | Obese: <11%  Diabetes: <11%  Asthma: 19.42% | NR | Moderate: 96.6%  Severe: <11%  IMV: <11% | Statistical significance (Omicron and pre-Omicron):  Severe: p < 0.001 |
| Niemann et al. ^44^  electronic health records (EHR)  Normal population with CLL | Retro- spective cohort study, (Denmark) | Period 1 (March 2020 - 31/12/20) | 59 (27/32) | 71 (64.5-80.5) | CLL: 100% | NR | Admission: 69.5%  ICU: 11.9%  Death Rate: 16.9% | Notes:  Period 1: Pre-Omicron in Denmark |
| - | - | Period 2  (1/1/21 - 25/11/21) | 40 (17/23) | 77 (68.2-82.0) | CLL: 100% | NR | Admission: 82.5%  ICU: 12.5%;  Death: 17.5% | Notes:  Period 2: first Omicron case in Denmark |
| - | - | Period 3 (26/11/21 - 31/12/21) | 32 (12/20) | 74.5 (69.8-83.0) | CLL: 100% | NR | Admission: 59.4%  ICU: 3.1%;  Deaths: 9.4% | Notes:  Period 3: Omicron emergence  5 out of the 6 deaths in this period were caused by the Delta variant.  During Period 3 (Omicron emergence) and Period 4 (Omicron dominance), mAbs were administered on an outpatient basis, which may in part explain the lower 30-day admission rates (56-60% vs 83%) |
| - | - | Period 4 (1/1/22 - 28/1/22) | 22 (9/13) | 76 (72.0-80.5] | CLL: 100% | NR | Admission: 54.5%  ICU: 0.0%  Death: 22.7% | Notes:  Period 4: Omicron sub-lineage BA.2 dominating from 1st January 2022.  General conclusions:  Patients above 70 with CLL and one or more comorbidities should be considered for closer monitoring and pre-emptive antiviral therapy upon a positive SARS-CoV-2 test. |
| Vallejo et al. ^45^  La Paz University Hospital    Pediatric population | Case series  (Spain) | Omicron | 2 (0/2) | 7-year-old and 11-year-old | None | No | ICU: 100%  CVST: 100%  Sinusitis: 100% | General conclusions:  Omicron may be associated with increased risk of thrombotic events.  Notes:  One case is Omicron, other case is unspecified but likely to be Omicron (December 2021)  First case: cavernous sinus and both internal jugular veins were affected.  Second case: superior sagittal sinus thrombosis |
| Brandal et al. ^46^ | Cohort study (Norway) | Omicron | 81 (35/46) | 38**±**8.6 | NR | NR | ASY: 1.23%  Mild: 98.77%  Hyposmia: 12.35%  Decreased appetite: 33.33%  Hypogeusia: 23.46% | Definitions:  Mild symptoms included hyposmia, decreased appetite, and ageusia, as well as runny nose, fatigue, sore throat, headache, fever, etc.  Vaccination status:  Unvaccinated: 11%  2 doses: 89%  Types of vaccines:  Pfizer: 55%  Moderna: 23% |
| Krutikov et al. ^47^  National hospital admission and mortality records  General population | Cohort study  (England) | Omicron | 1864 (1287/577) | 84.6 (77.8-90.0) | NR | 12.7% | Admission: 4.5%  Death: 5.3% | Vaccination status:  Unvaccinated: 12.7%  2 doses: 12.3%  3 doses: 75.1%  Types of vaccines:  AstraZeneca: 59.6%  Pfizer: 35.3%  NR: 5.1%  Notes:  Admissions did not include deaths.  The number of deaths due to Omicron is incomplete because of the time lag in data collection.  Statistical significance (Omicron and Delta):  Admissions: p<0.0001  Death: p<0.0001  Vaccination: p<0.0001  Previous infection: p<0.0001 |
| - | - | Delta | 400 (272/128) | 84.5  (78.0-90.1) | NR | 4.3% | Admission: 10.5%  Death: 12.8% | Vaccination status:  Unvaccinated: 16.5%  2 doses: 66.2%  3 doses: 17.3%  Types of vaccines:  AstraZeneca: 65.3%  Pfizer: 30.5%  NR: 4.2% |
| AraujodaSilva et al. ^48^  Pediatric hospitals  Hospitalized children  (Aged 0-18) | Observ- ational retro- spective cohort study  (Brazil) | Pre- Omicron  (Variant NR) | 240 (104/136) | NR | NR | NR | Admission: 100%  PICUs: 53.3%  Death: 2.5% | Age was reported as ranges:  0-2 yr: 80  2-5 yr: 52  5-11 yr: 70  12-18 yr: 38  Notes:  Pre-Omicron period was 2020-2021.  Admissions included patients in PICU and deaths. |
| - | - | Omicron | 60 (30/30) | NR | NR | NR | Admission: 100%  PICUs: 51.6%  Death: 1.7% | Age was reported as ranges:  0-2 yr: 32  2-5 yr: 6  5-11 yr: 17  12-18 yr: 5  Notes:  Omicron wave was in 2022. Children aged from 12-17 were only vaccinated with two doses in December 2021, and children aged 5-11 received vaccines starting January 2022.  96.7% did not receive complete COVID-19 vaccine scheme.  Statistical significance (Omicron and pre-Omicron):  PICUs: p=0.817  Death: p=0.894 |
| Kim et al. ^49^  National Medical Center  General population | Case series (South Korea) | Omicron | 40 (22/18) | 39.5 (16-50.3) | NR | 0% | ASY: 47.5%  Mild: 52.5%  Loss of taste/smell: 2.5%  Needed antipyretics: 7.5%  Lung infiltrations on CXR/CT: 15% | Definitions:  Mild symptoms included sore throat, fever, headache, cough, sputum, runny nose, loss of taste/smell, etc  Vaccination status:  Unvaccinated: 55%  1 dose: 2.5%  2 doses: 40%  3 doses: 2.5%  Vaccine types:  Pfizer: 41%  Moderna: 29%  AstraZeneca: 18%  J&J: 12%  General Conclusions:  Omicron variant might cause less severe disease than the Delta variant. However, the limitations of the study such as a small number of mostly young patients without comorbidities should be kept in mind. |
| Madhi et al. ^50^  South African National Institute for Communicable Diseases  General population | Sero-  epidem- iologic survey  (South Africa) | Wild-Type | 232,130 | NR | NR | NR | Admission: 14.35%  Recorded death: 2.78%  Excess death: 5.81% | Notes:  Values for admission, recorded deaths, and excess deaths, were recorded at varying dates within the same wave.  Definitions:  Excess deaths are the number  of deaths that are happening more than normally expected.” |
| - | - | Beta | 182,564 | NR | NR | NR | Admission: 16.81%  Recorded death: 3.88%  Excess death: 6.56% |  |
| - | - | Delta | 511,638 | NR | NR | NR | Admission: 12.05%  Recorded death: 2.79%  Excess death: 5.97% |  |
| - | - | Omicron | 226,932 | NR | NR | NR | Admission: 6.96%  Recorded death: 0.49%  Excess death: 0.85% |  |
| Lewnard et al. ^51^  Kaiser Permanente Southern California healthcare system  General population | Cross sectional cohort (United States) | Delta | 23305 (12926/10379) | NR | MI: 0.7%  HF: 1.0%  PVD: 3.5%  CeVD: 0.8%  Diabetes: 8.8%  Liver disease: 2.5%  Kidney disease: 2.2%  Asthma: 7.5%  COPD: 8.0%  Apnea: 3.4%  HT: 3.2%  HIV: 0.1%  Cancer: 1.5%  Depression: 8.0%  Dementia: 0.2%  Hyperlipidemia: 15.1%  Rheumatic disease: 0.9%  PUD: 0.2%  Organ transplant: 0.2%  Paraplegia and hemiplegia: 0.2% | 0.4% | SY: 1.37%  Admission: 1.57%  MV: 0.01%  ICU: 0.12%  Death: 0.08% | Age reported as ranges.  <1 yr: 391  1-4 yr: 1,023  5-9 yr: 1,321  10-19 yr: 3,218  20-29 yr: 3,096  30-39 yr: 3,942  40-49 yr: 3,770  50-59 yr: 3,310  60-69 yr: 1,973  70-79 yr: 802  80+ yr: 264  Definitions:  SY was reported as “symptomatic hospital admission”  Vaccination status:  Unvaccinated: 9802  1 dose J&J: 717  1 dose J&J + booster: 170  1 dose Pfizer or Moderna: 646  2 doses Pfizer or Moderna: 9714  3 doses Pfizer or Moderna: 2256  Notes:  This study does not specifically mentioned whether admissions includes ICU, MV, and death, but it seems like it does.  This data was recorded from Dec 2021-Jan 2022. |
| - | - | Omicron | 222688 (123227/ 99461) | NR | MI: 0.5%  HF: 0.7%  PVD: 2.6%  CeVD: 0.6%  Diabetes: 7.8%  Liver disease: 2.3%  Kidney disease: 1.6%  Asthma: 6.8%  COPD: 7.2%  Apnea: 3.0%  HT: 2.7%  HIV: 0.1%  Cancer: 1.3%  Depression: 8.0%  Dementia: 0.1%  Hyperlipidemia: 13.6%  Rheumatic disease: 0.7%  PUD: 0.1%  Organ transplant: 0.1%  Paraplegia and hemiplegia: 0.1% | 0.5% | SY: 0.55%  Admission: 0.73%  MV: 0.01%  ICU: 0.03%  Death: 0.01% | Age reported as ranges.  <1 yr: 391  1-4 yr: 1,023  5-9 yr: 1,321  10-19 yr: 3,218  20-29 yr: 3,096  30-39 yr: 3,942  40-49 yr: 3,770  50-59 yr: 3,310  60-69 yr: 1,973  70-79 yr: 802  80+ yr: 264  Vaccination status:  Unvaccinated: 65480  1 dose J&J: 6874  1 dose J&J + booster: 2329  1 dose Pfizer or Moderna: 6266  2 doses Pfizer or Moderna: 108223  3 doses Pfizer or Moderna: 33516  Notes:  This data was recorded from Dec 2021-Jan 2022. |
| - | - | Omicron BA2 | 1905 (1040/865) | NR | NR | 0.4% | SY: 1.26%  Admission: 1.47%  MV: 0.05%  ICU: 0.05%  Death: 0.1% | Age reported as ranges.  <1 yr: 53  1-4 yr: 104  5-9 yr: 89  10-19 yr: 217  20-29 yr: 197  30-39 yr: 315  40-49 yr: 271  50-59 yr: 272  60-69 yr: 230  70-79 yr: 102  80+ yr: 39  Vaccination status:  Unvaccinated: 594  1 dose J&J: 34  1 dose J&J + booster: 38  1 dose Pfizer or Moderna: 33  2 doses Pfizer or Moderna: 565  3 doses Pfizer or Moderna: 641  Notes:  This data was recorded from Feb 2022-Mar 2022.  0.3% of unvaccinated patients were previously infected. NR for vaccinated. |
| - | - | Omicron BA1 | 12756 (6844/5912) | NR | NR | 0.6% | SY: 1.15%  Admission: 1.33%  MV: 0.01%  ICU: 0.05%  Death: 0.13% | Age reported as ranges.  <1 yr: 157  1-4 yr: 608  5-9 yr: 904  10-19 yr: 1432  20-29 yr: 1393  30-39 yr: 2421  40-49 yr: 2090  50-59 yr: 1624  60-69 yr: 1240  70-79 yr: 632  80+ yr: 218  Vaccination status:  Unvaccinated: 3858  1 dose J&J: 273  1 dose J&J + booster: 203  1 dose Pfizer or Moderna: 287  2 doses Pfizer or Moderna: 4314  3 doses Pfizer or Moderna: 3821 |
| Paredes et al. ^52^  Washington Disease Reporting System  General Population | Retro- spective cohort study  (United States) | Ancestral | 5178 (2410/2595) | NR | NR | 0% | Admission: 2.3% | Age was reported as a range:  0-9: 418  10-19: 814  20-29: 1053  30-39: 984  40:49: 741  50-59: 569  60-69: 378  70-79: 139  80-89: 60  90+: 22 |
| - | - | Alpha | 8729 (4234/4328) | NR | NR | 0% | Admission: 2.7% | Age was reported as a range:  0-9: 888  10-19: 1561  20-29: 1948  30-39: 1640  40:49: 1273  50-59: 797  60-69: 389  70-79: 150  80-89: 68  90+: 15  Vaccination status:  Unvaccinated: 8412  1 dose: 309  1 dose+booster: 2  Vaccination covariate included Pfizer, Moderna, and J&J. |
| - | - | Beta | 231 (104/123) | NR | NR | 0% | Admission: 4.8% | Age was reported as a range:  0-9: 888  10-19: 1561  20-29: 1948  30-39: 1640  40:49: 1273  50-59: 797  60-69: 389  70-79: 150  80-89: 68  90+: 15  Vaccination status:  Unvaccinated: 221  1 dose: 10  1 dose+booster: 0  Vaccination covariate included Pfizer, Moderna, and J&J. |
| - | - | Gamma | 2103 (1026/1040) | NR | NR | 0% | Admission: 5.3% | Age was reported as a range:  0-9: 198  10-19: 272  20-29: 527  30-39: 432  40:49: 317  50-59: 186  60-69: 108  70-79: 33  80-89: 22  90+: 8  Vaccination status:  Unvaccinated: 1946  1 dose: 154  1 dose+booster: 1  Vaccination covariate included Pfizer, Moderna, and J&J. |
| - | - | Delta | 33115 (16053/16513) | NR | NR | 0% | Admission: 3.3% | Age was reported as a range:  0-9: 3412  10-19: 4548  20-29: 6485  30-39: 6285  40:49: 4583  50-59: 3529  60-69: 2429  70-79: 1223  80-89: 462  90+: 159  Vaccination status:  Unvaccinated: 23108  1 dose: 9434  1 dose+booster: 565  Vaccination covariate included Pfizer, Moderna, and J&J. |
| - | - | Epsilon | 3526 (1666/1739) | NR | NR | 0% | Admission: 2.1% | Age was reported as a range:  0-9: 307  10-19: 590  20-29: 760  30-39: 657  40:49: 524  50-59: 383  60-69: 209  70-79: 62  80-89: 31  90+: 3  Vaccination status:  Unvaccinated: 3438  1 dose: 86  1 dose+booster: 2  Vaccination covariate included Pfizer, Moderna, and J&J. |
| - | - | Iota | 638 (292/330) | NR | NR | 0% | Admission: 2.0% | Age was reported as a range:  0-9: 54  10-19: 113  20-29: 154  30-39: 128  40:49: 93  50-59: 56  60-69: 25  70-79: 11  80-89: 3  90+: 1  Vaccination status:  Unvaccinated: 620  1 dose: 18  1 dose+booster: 0  Vaccination covariate included Pfizer, Moderna, and J&J. |
| - | - | Omicron | 5362 (2733/2551) | NR | NR | 0% | Admission: 0.7% | Age was reported as a range:  0-9: 362  10-19: 1004  20-29: 1362  30-39: 991  40:49: 780  50-59: 470  60-69: 260  70-79: 84  80-89: 35  90+: 14  Vaccination status:  Unvaccinated: 2000  1 dose: 2809  1 dose+booster: 553  Vaccination covariate included Pfizer, Moderna, and J&J. |
| Lee et al. ^53^  National surveillance system  Inbound international travelers | Retro- spective Observat- ional Cohort Study  (Korea) | Omicron | 80 (43/37) | NR | NR | NR | ASY: 27.5%  Mild: 72.5%  Anosmia/ageusia: 1.3%  Severe: 0%  Death: 0% | Age was reported as ranges:  <20: 17  20-39: 37  40-59: 19  60+: 7  Definition:  Mild symptoms included fever, chills, cough, sputum, sore throat, headache, myalgia, and anosmia/ageusia.  Vaccination status:  Unvaccinated: 48  1 dose: 5  2 doses: 25  Only 79 patients had VS reported  Vaccine types:  Pfizer: 52%  Moderna: 20%  J&J: 16%  AstraZeneca: 12% |
| Peralta-Santos et al. ^54^  Three laboratories, National Institute of Health Dr Ricardo Jorge, national epidemiological and laboratory surveillance platform (SINAVE), electronic death certificate platform (SICO), electronic vaccination platform (VACINAS), hospital admission platform of the National Health System (SONHO)  General population | Cohort study  (Portugal) | Omicron | 6581 (3366/3215) | 37.1±14.8 | NR | 6.8% | Admission: 0.2%  ICU: 0%  Death: 0% | Vaccination status:  Unvaccinated: 785  1 dose of Pfizer, Moderna, or  Astrazeneca: 109  2 doses of Pfizer, Moderna, or AstraZeneca or 1 dose J&J: 5392  3 doses: 295 |
| - | - | Delta | 9397 (4632/4765) | 43.4±15.8 | NR | 1.6% | Admission: 1.39%  ICU: 0.18%  Death: 0.3% | Vaccination status:  Unvaccinated: 1032  1 dose of Pfizer, Moderna, or  Astrazeneca: 128  2 doses of Pfizer, Moderna, or AstraZeneca or 1 dose J&J: 8036  3 doses: 201 |
| Sharma et al. ^55^  Rajasthan received at SMS Medical College Jaipur  General population | Cohort study  (India) | Omicron | 291 (126/165) | NR | NR | 43.2% | ASY: 56.7%  Mild: 33.3%  Moderate: 10.0%  Admission: 1.03%  Oxygenation: 0.7%  Death: 0.3% | Age was reported as ranged:  0-9: 6  10-18: 44  19-29: 64  30-39: 39  40-49: 59  50-59: 36  60-69: 28  70-79: 14  >=80: 1  Vaccination status:  Unvaccinated: 40  Partially vaccinated: 15  Fully vaccinated: 236  Vaccine types:  Covishield: 70.3%  Covaxin: 20.9 %  Pfizer: 6.6%  Astrazaneca: 2.2% |
| Espenhain et al. ^57^  Routine Danish surveillance  General population | Cohort study (Denmark) | Delta | 19137 (9637/9500) | NR | NR | 0.9% | SY: NR  Admission: 1.5%  ICU: 0.11%  Death: 0.07% | Age was reported as a ranges:  0-9: 3081  10-14: 2434  15-19: 962  20-29: 2317  30-39: 2548  40-49: 2973  50-64: 2952  >=65: 1870  Vaccination status:  Unvaccinated: 8199  1 dose: 484  2 doses of Pfizer, AstraZeneca, or Moderna or 1 dose of J&J: 9269  3 doses: 597  Notes:  Unclear whether admissions include ICU and death or not. |
| - | - | Omicron | 785 (352/433) | NR | NR | 4.3% | SY: 65%  Admission: 1.2%  ICU: 0.13%  Death: 0% | Age was reported as a ranges:  0-9: 30  10-14: 23  15-19: 102  20-29: 214  30-39: 110  40-49: 111  50-64: 144  >=65: 51  Vaccination status:  Unvaccinated: 111  1 dose: 19  2 doses of Pfizer, AstraZeneca, or Moderna or 1 dose of J&J: 599  3 doses: 56  Notes:  SY was self-reported. Of all Omicron patients, 65% reported to have symptoms, 20% did reported no symptoms, and 15% were unknown. |
| Boscolo-Rizzo et al. ^58^ | Prospective Cohort  (Italy) | Omicron | 338 (183/155) | 46 | Alcohol drinking: 27.5%  IC: 3.8%  Diabetes: 5.8%  Obesity: 8.9%  CVD: 16.6%  Malignancy: 3.6%  Chronic respiratory diseases: 8.3%  Kidney failure: 5.3%  Liver disease: 4.7% | 5.3% | Mild: 100%  Smell and taste impairment: 19.2%  Only smell impairment: 5.3%  Only taste impairment: 7.7% | Age was reported as median (range): 46 (34-59).  Definition:  Mild symptoms included smell and taste impairments, dry cough, fever, loss of appetite, chest and joint pain, etc.  .  Vaccination status:  Unvaccinated: 49  Partially vaccinated: 23  2 doses: 266 |
| Auvigne et al. ^59^  COVID-19 national  surveillance databases  General population | Retro- spective cohort study (France) | Delta | 92182 (50265/41917) | NR | Medium risk: 12.5%  Very high risk: 3.1% | NR | Admission: 0.89%  Severe hospital events: 0.89%  ICU: 0.38% “  Death: 0.13% “ | Age was reported as ranges:  18-40: 50073  40-65: 35353  65-80: 5219  80+: 1537  Definitions:  Medium-risk comorbidities included obesity, diabetes, chronic renal failure, chronic obstructive pulmonary disease, respiratory failure, HTN, and HF. Very-high-risk comorbidities included cancers, hematological malignancies undergoing chemotherapy, severe chronic kidney disease, chronic dialysis, solid organ transplants, hematopoietic stem cell allografts, and chronic multi-disease conditions with two or more organ failures, certain rare diseases and those at particular risk of infection, and Down’s syndrome.  Vaccination status:  Unvaccinated: 32421  2 doses: 53362  3 doses: 6399  Vaccine types (first dose for both Delta and Omicron):  Pfizer: 84%  Moderna: 9.8%  AstraZeneca: 4.8%  Janssen: 1.2% |
| - | - | Omicron | 92182 (49996/42186) | NR | Medium risk: 11.3%  Very high risk: 2.6% | NR | Admission: 0.12%  Severe hospital events: 0.12%  ICU: 0.38% “  Death: 0.13% “ | Age was reported as ranges:  18-40: 50073  40-65: 35353  65-80: 5219  80+: 1537  Statistical significance (Omicron and Delta):  Severe hospital events: p<0·001 |
| Christensen et al. ^60^  Houston Methodist Hospital Database  General population | Cohort study (United States) | Omicron | 4468 (2584/1884) | 44.3 | NR | NR | Admission: 19.8%  Room air: 43.6%  IMV: 5.5%  NIV: 7.1%  ECMO: 0.1%  HFOT: 8.1%  LFOT: 35.5%  Death: 0.9% | Age was reported as a median.  Vaccination status:  Unvaccinated: 1815  1 dose: 156  2 doses of Pfizer or Moderna or 1 dose of J&J: 1786  3 doses: 711  Vaccine types (2-3 doses):  Pfizer: 73%  Moderna: 22%  J&J: 5% |
| - | - | Alpha | 3149 (1617/1532) | 50.0 | NR | NR | Admission: 54.6%  Room air: 18.6%  IMV: 8.4%  NIV: 8.7%  ECMO: 0.3%  HFOT: 16.7%  LFOT: 39.8%  Death: 5.4% | Age was reported as a median.  Vaccination status:  Unvaccinated: 3048  Fully vaccinated: 101  Statistical significance (Omicron and Alpha):  Admission: p <0.0001  IMV: p<0.0001  NIV: p<0.0001  ECMO: p<0.0001  HFOT: p<0.0001  LFOT: p<0.0001  Death: p<0.0001 |
| - | - | Delta | 15728 (8123/7605) | 48.3 | NR | NR | Admission: 43.1%  Room air: 19.3%  IMV: 10.7%  NIV: 9.5%  ECMO: 0.3%  HFOT: 26.5%  LFOT: 33.8%  Death: 5.3% | Age was reported as a median.  Vaccination status:  Unvaccinated: 1815  1 dose: 494  2 doses:: 3679  3 doses: 140  Statistical significance (Omicron and Delta):  Admission: p <0.0001  IMV: p<0.0001  NIV: p<0.0001  ECMO: p<0.0001  HFOT: p<0.0001  LFOT: p<0.0001  Death: p<0.0001 |
| Houhamdi et al. ^61^  Institut Hospitalo-Universitaire Méditerranée Infection, Marseille, France  General population | Single- center retrospective cohort study (France) | Omicron | 1119 (611/508) | 33 | NR | NR | ASY: 36.5%  SY: 63.5%  Admission: 1.9%  ICU: 0.1%  Death: 0.1% | Age was reported as median (range): 33 (0-93)  Vaccination status:  Unvaccinated: 443  1 dose: 30  2 doses: 257  3 doses: 95  4 doses: 1  Statistical significance (Omicron and Delta):  Self-reported symptoms: p<0.0001  Admission: p<0.0001  ICU: p<0.0001  Death: p<0.0001 |
| - | - | Delta | 3075 (1576/1499) | 42 | NR | NR | ASY: 22.4%  SY: 77.6%  Admission: 11.9%  ICU: 3.1%  Death: 1.3% | Age was reported as median (range): 42 (0-100)  Notes:  Total number of Delta is much higher than total number of unvaccinated and vaccinated patients, but paper did not explain why.  Symptoms were self-reported but not defined specifically in the paper.  Was not mentioned whether admissions included ICU patients. “Most of the positive patients … were hospitalized in dedicated infectious disease units at IHU, according to the severity of the disease. When required, patients were transferred to ICU at AP-HM”  Patients with a previous CoVid infection were considered to have received one dose of a vaccine.  Vaccination status:  Unvaccinated: 1166  1 dose: 64  2 doses: 630  3 doses: 93  4 doses: 1 |
| Davies et al. ^62^  Western Cape Provincial Health Data Centre (WCPHDC)  General population | Cohort study (South Africa) | Omicron | 5144 (3407/1737) | NR | Diabetes: 7.9%  HTN: 16.3%  Kidney disease: 1.7%  Chronic pulmonary disease/asthma: 7.8%  Previous TB: 7.2%  Current TB: 1.2%  HIV: 13.8% | 11.3% | Admission (not severe): 6.3%  Severe admission: 0.9%  Death: 0.8% | Age was reported as ranges:  20-39: 3318  40-49: 851  50-59: 571  60-69: 266  >=70: 138  Notes:  Admissions does not include deaths or severe admissions. Severe admission is admission to an intensive care unit, mechanical ventilation or prescription of oral or intravenous steroids; not reported for wave one as steroids not widely used until after 16 June 2020.  Vaccination status:  Unvaccinated: 2934  1 dose Pfizer: 269  2 doses Pfizer or 1 dose J&J: 1941 |
| - | - | Delta | 4403 (2765/1638) | NR | Diabetes: 17.4%  HTN: 26.3%  Kidney disease: 4.7%  Chronic pulmonary disease/asthma: 8.0%  Previous TB: 6.3%  Current TB: 1.0%  HIV: 6.6% | 3.2% | Admission (not severe): 10.4%  Severe admission: 4.3%  Death: 5.7% | Age was reported as ranges:  20-39: 1923  40-49: 847  50-59: 787  60-69: 472  >=70: 374  Vaccination status:  Unvaccinated: 4205  1 dose Pfizer: 26  2 doses Pfizer or 1 dose J&J: 127 |
| - | - | Beta | 3902 (2526/1376) | NR | Diabetes: 16.6%  HTN: 24%  Kidney disease: 3.7%  Chronic pulmonary disease/asthma: 7.0%  Previous TB: 7.5%  Current TB: 1.7%  HIV: 20.8% | 1.9% | Admission (not severe): 11.0%  Severe admission: 3.4%  Death: 3.4% | Age was reported as ranges:  20-39: 1915  40-49: 767  50-59: 624  60-69: 360  >=70: 236 |
| - | - | Ancestral | 3304 (2415/889) | NR | Diabetes: 12.3%  HTN: 20.6%  Kidney disease: 2.5%  Chronic pulmonary disease/asthma: 5.5%  Previous TB: 9.1%  Current TB: 1.0%  HIV: 20.8% | 0% | Admission (not severe): 8.2%  Severe admissions: N/A  Death: 1.8% | Age was reported as ranges:  20-39: 2034  40-49: 666  50-59: 391  60-69: 144  >=70: 69 |
| Hajjo et al. ^63^  Jordan CDC  General population | Question- naire/survey  (Jordan) | Omicron | 500 (229/225) | 30 | NR | 8.6% | ASY: 31.4%  Mild: 45.4%  Moderate: 5.00%  Severe: 1.00%  Loss of tase and smell: 1.2% | 46 of the patients had unspecified gender; Age was reported as median.  Vaccination status:  Unvaccinated: 167  2 doses: 269  3 doses: 64 |
| Accorsi et al. ^64^  National pharmacy-based testing program  General adult population | Case-control analysis  (United States) | Omicron | 13098 (7577/5497) | NR | HTN: 12.7%  Overweight: 11.9%  Smoker: 6.2%  Diabetes: 4.8%  Lung disease or asthma: 3.5%  Heart condition: 2.9%  Kidney disease: 0.2%  Liver disease: 0.1%  1 condition: 18.9%  2+ conditions: 9.9% | 0% | SY: 100% | Age reported as ranges:  18-24: 2471  25-34: 4591  35-44: 2588  45-54: 1707  55-64: 1153  >=65: 588  Vaccination status:  Unvaccinated: 3412  2 doses: 7245  3 doses: 2441  Notes:  This study was looking into the characteristics and variants in symptomatic patients with CoVid-19. |
| - | - | Delta | 10293 (5392/4881) | NR | HTN: 13.9%  Overweight: 12.3%  Smoker: 10.9%  Diabetes: 5.1%  Lung disease or asthma: 3.9%  Heart condition: 3.5%  Kidney disease: 0.2%  Liver disease: 0.2%  1 condition: 20.3%  2+ conditions: 12.0% | 0% | SY: 100% | Age reported as ranges:  18-24: 1713  25-34: 3191  35-44: 2171  45-54: 1515  55-64: 1131  >=65: 572  Vaccination status:  Unvaccinated: 5044  2 doses: 4570  3 doses: 679 |
| Ulloa et al. ^65^  Public Health Case and Contact Management Solution  General population | Retro- spective population- wide matched cohort study  (Canada) | Delta | 24432 (12038/12331) | 33.0 (13.0-49.0) | NR | NR | Admission: 2.28%  ICU: 0.47%  Death: 0.54% | Notes:  Admissions do not include ICU and deaths.  The results reported in this table are based on the full cohort, not matched.  Vaccination status:  Unvaccinated: 10900  1 dose: 1409  2 doses: 10084  3 doses: 2039 |
| - | - | Omicron | 37296 (18682/18577) | 30.0 (21.0-44.0) | NR | NR | Admission: 0.24%  ICU: 0.02%  Death: 0.01% | Vaccination status:  Unvaccinated: 4784  1 dose: 1859  2 doses: 26681  3 doses: 3972 |
| CDC team ^66^  National SARS-CoV-2 Strain Surveillance, diagnostic laboratories, and public repositories  General population | Cohort study (United States) | Omicron | 43 (25/17) | NR | NR | 14% | SY: 93%  Mild: 16.28%  Admission: 2%  Death: 0%  Mild symptoms included:  Cough: 89%  Fatigue: 65%  Congestion/runny nose: 59%  Fever: 38%  Nausea/vomiting: 22%  Shortness of breath/difficulty breathing: 16%  Diarrhea: 11%  Hyposmia/ Hypogeusia: 8% | Age was reported as ranges:  <18: 4  18-39: 25  40-64: 10  >=65: 4  Notes:  Previous SARS-CoV-2 infections were unknown for 16 patients.  Definitions:  Mild symptoms included cough, fatigue, congestion, fever, etc. The paper mentioned that 7 (16.28%) of the 43 patients had mild symptoms, but did not specify for the other patients.  Vaccination status:  Unvaccinated: 8  2 doses: 20  3 doses: 14 |
| Klein et al. ^67^  VISION Network  Children and adolescents with ED or UC encounters | Observa- tional cohort study (United States) | Omicron | 5484 (4883/4369) “ | NR | Chronic respiratory condition: 4.93% “  Chronic nonrespiratory condition: 4.10% “ | NR | Admission: 2.75% | Age was reported as ranges:  5-11 yr: 2527  12-15 yr: 1777  16-17 yr: 1180  Vaccination status:  Unvaccinated: 4434  2 doses Pfizer: 638  3 doses Pfizer: 16  Definitions:  Chronic respiratory condition was defined as the presence of discharge code for asthma, sleep apnea, or other lung disease using ICD-9 and ICD-10 diagnosis codes.  Chronic nonrespiratory condition was defined as the presence of discharge code for heart failure, ischemic heart disease, hypertension, other heart disease, stroke, other cerebrovascular disease, diabetes type I or II, other diabetes, metabolic disease, clinical obesity, clinically underweight, renal disease, liver disease, blood disorder, immunosuppression, organ transplant, cancer, neurologic disorder, musculoskeletal disorder, Down Syndrome, congenital heart disease, neurologic conditions, muscular dystrophy, sickle cell disease, prematurity (<24 weeks), developmental delay, technology dependence, or chronic gastrointestinal disease/irritable bowel syndrome. |
| - | - | Delta | 3655 (4883/4369) “ | NR | Chronic respiratory condition: 4.93% “  Chronic nonrespiratory condition: 4.10% “ | NR | Admission: 6.13% | Age was reported as ranges:  5-11 yr: 249  12-15 yr: 2090  16-17 yr: 1316  Vaccination status:  Unvaccinated: 3169  2 doses Pfizer: 237  3 doses Pfizer: 0 |

Comorbidity abbreviations: CeVD: Cerebrovascular disease, CLL: Chronic lymphocytic leukemia COPD: Chronic obstructive pulmonary disease, CVD: Cardiovascular disease, HF: Heart failure, HIV: Human immunodeficiency virus, HT: Hypothyroidism, HTN: Hypertension, IC: Immunocompromised/Immunosuppressed, PUD: Peptic ulcer disease, PVD: Peripheral vascular disease, TB: Tuberculosis.

Severity abbreviations: ASY: Asymptomatic, CVST: Cerebral venous sinus thrombosis, ECMO: Extracorporeal membrane oxygenation, ED: Emergency department, HFOT: High flow oxygen therapy, ICU: Intensive care unit, IMV: Invasive mechanical ventilation, LFOT: Low flow oxygen therapy, MI: Myocardial infarction, MV: Mechanical ventilation, NIV: Noninvasive ventilation, SY: Symptomatic.

General abbreviations: NR: Not reported, UAI: Upper airway infection.

“ Not stratified on COVID-19 variant

- Data is same as data in row above
