# Supplementary material for: Severity of the Omicron SARS‐CoV‐2 variant compared with the previous lineages: A systematic review
Source: J Cell Mol Med. 2023 May 18;27(11):1443–64. doi: 10.1111/jcmm.17747 (PMC10243162; doi:10.1111/jcmm.17747)
Supplement: Supplementary file 4 — TABLE S4 Hospital admissions reported according to the vaccination status in cases infected with Omicron or other variants. [file JCMM-27-1443-s007.docx]

**Supplementary Table 4: Hospital admissions reported according to the vaccination status in cases infected with Omicron or other variants**

| Study | Vaccine type | Omicron (no of doses) (%) | | | | | | Other variants (no of doses) (%) | | | | | | | P values or CI  Risk factors |
| --- | --- | --- | --- | --- | --- | --- | --- | --- | --- | --- | --- | --- | --- | --- | --- |
|  |  | Unvaccinated | Partially vaccinated | Fully vaccinated | Boosted | Dose NR | VS NR | Variant | Unvaccinated | Partially vaccinated | Fully vaccinated | Boosted | Dose NR | VS NR |  |
| Fall et al. ^5^ | Pfizer, Moderna, or J&J | 1.8 | - | 4.3 | 2.2 | - | - | Delta | 15.4 | - | 10.6 | 15.7 | - | - | P<0.0001 (Omicron & Delta)  p=0.7 (Omicron boosted and unvaccinated)  p=1.0 (Delta boosted and unvaccinated)  p=0.00007 (Delta and Omicron boosted) |
| Goga et al. ^6^ | J&J | - | - | - | - | 3.34  (1-2 doses) | - | Delta | - | - | - | - | 5.59  (1-2 doses) | - | p<0.001 (Beta, Delta, and Omicron vaccinated) |
| Abu-Raddad et al. ^7^ ** | Pfizer | - | - | 0.14 | 0 | - | - | - | - | - | - | - | - | - |  |
|  | Moderna | - | - | 0 | 0 | - | - | - | - | - | - | - | - | - |  |
| Lee et al. ^11^ | Pfizer, Moderna, or J&J | - | - | - | - | - | 39.3 (0-3 doses) | - | - | - | - | - | - | - | Cancer |
| Veneti et al. ^12^ | NR | 0.186 | 0.16 | 0.22 | 0.87 | - | - | Delta | 1.46 | 0.40 | 0.64 | 3.65 | - | - |  |
| Wang et al. ^14^ ** | NR | 1.04 | - | - | - | - | - | Delta | 3.14 | - | - | - | - | - | Pediatric |
| Maisa et al. ^16^ | NR | - | - | - | - | - | 2  (0-3 doses) | - | - | - | - | - | - | - |  |
| Tseng et al. ^24^ | Moderna | - | 4.3 | 3.7 | 4.2 | - | - | Delta | - | 4.9 | 4.7 | 4.9 | - | - |  |
| Thompson et al. ^25^ | Moderna, Pfizer, or mixture | 5.12 | - | 3.81 | 4.62 | - | - | Delta | 39.1 | - | 34.0 | 44.6 | - | - | Patients who had ED or UC encounters |
| Davies et al. ^62^ | Pfizer or J&J | - | - | - | - | - | 6.3 | Ancestral | 8.2 | - | - | - | - | - |  |
|  | NR | - | - | - | - | - | - | Beta | 11.0 | - | - | - | - | - |  |
|  | Pfizer or J&J | - | - | - | - | - | - | Delta | - | - | - | - | - | 10.4 |  |
| Ferdinands et al. ^28^ | Pfizer, Moderna, or mix | 13.5 | - | 11.7 | 14.2 | - | - | Delta | 49.7 | - | 40.7 | 56.2 | - | - |  |
| Gray et al. ^29^ | J&J | 3.9 | - | 1.2 | - | - | - | - | - | - | - | - | - | - |  |
| Sami et al. ^32^ | Pfizer, Moderna, or J&J | - | - | - | - | 0.84  (1-3 doses) | - | - | - | - | - | - | - | - |  |
|  | Pfizer, Moderna, or J&J | - | - | - | - | 0  (1-3 doses) | - | - | - | - | - | - | - | - |  |
| Patalon et al. ^33^ | Pfizer | - | - | 0.72 | 0.21 | - | - | - | - | - | - | - | - | - |  |
| Dinh et al. ^35^ | NR | - | - | - | - | - | 0.06 | Alpha | - | - | - | - | - | 0.19 | p<0.0001 (Alpha & Omicron) |
|  | NR | - | - | - | - | - | - | Delta | - | - | - | - | - | 0.46 |  |
| Ebell et al. ^34^ | NR | - | - | - | - | - | - | NR | - | - | - | - | - | 4.18 |  |
| Wolter et al. ^37^ | NR | - | - | - | - | - | 3.4  (BA1)  3.6  (BA2) | - | - | - | - | - | - | - |  |
| Wang et al. ^38^ ** | Pfizer, Moderna, or J&J | - | - | - | - | - | 1.75 | Delta | - | - | - | - | - | 3.95 |  |
| Wang et al. ^39^ ** | NR | - | - | - | - | - | 2.6 | Delta | - | - | - | - | - | 4.44 |  |
| Wolter et al. ^56^ | NR | - | - | - | - | - | 2.43 | NR | - | - | - | - | - | 12.76 |  |
| Marks et al. ^15^ | NR | 65.0 | - | 55.5 | - | - | - | Delta | 70.8 | - | 35.8 | - | - | - | p<0.001 (vaccinated & unvaccinated) |
| Maslo et al. ^40^ | Pfizer or J&J | - | - | - | - | - | 41.3 | Ancestral | - | - | - | - | - | 67.8 |  |
|  | Pfizer or J&J | - | - | - | - | - | - | Beta | - | - | - | - | - | 69.0 |  |
|  | Pfizer or J&J | - | - | - | - | - | - | Delta | - | - | - | - | - | 69.3 | p<0.001 (Delta & Omicron) |
| Jassat et al. ^41^ | Pfizer or J&J | - | - | - | - |  | 7.29 | D614G | - | - | - | - | - | 12.72 | p<0.001 (D614G & Omicron) |
|  | Pfizer or J&J | - | - | - | - | - | - | Beta | - | - | - | - | - | 12.43 | p<0.001 (Beta & Omicron) |
|  | Pfizer or J&J | - | - | - | - | - | - | Delta | - | - | - | - | - | 9.84 | p<0.001 (Delta & Omicron) |
| Niemann et al. ^44^ | NR | - | - | - | - | - | 59.4 (Period 3) | (Period 1) | - | - | - | - | - | 69.5 | CLL |
|  | NR | - | - | - | - | - | 54.5 (Period 4) | First case Omicron (Period 2) | - | - | - | - | - | 82.5 |  |
| Krutikov et al. ^47^ | Pfizer, AstraZeneca, or NR | - | - | - | - | - | 4.5 | Delta | - | - | - | - | - | 10.5 | p<0.0001 (Omicron & Delta) |
| Madhi et al. ^50^ | NR | - | - | - | - | - | 6.96 | Wild- Type | - | - | - | - | - | 14.35 |  |
|  | NR | - | - | - | - | - | - | Beta | - | - | - | - | - | 16.81 |  |
|  | NR | - | - | - | - | - | - | Delta | - | - | - | - | - | 12.05 |  |
| Lewnard et al. ^51^ | Pfizer, Moderna, or J&J | - | - | - | - | - | 0.73 | Delta | - | - | - | - | - | 1.57 |  |
|  | Pfizer, Moderna, or J&J | - | - | - | - | - | 1.47 | - | - | - | - | - | - | - |  |
|  | Pfizer, Moderna, or J&J | - | - | - | - | - | 1.33 | - | - | - | - | - | - | - |  |
| Paredes et al. ^52^ ** | Pfizer, Moderna, or J&J | - | - | - | - | - | 0.7 | Alpha | - | - | - | - | - | 2.7 |  |
|  | Pfizer, Moderna, or J&J | - | - | - | - | - | - | Beta | - | - | - | - | - | 4.8 |  |
|  | Pfizer, Moderna, or J&J | - | - | - | - | - | - | Gamma | - | - | - | - | - | 5.3 |  |
|  | Pfizer, Moderna, or J&J | - | - | - | - | - | - | Delta | - | - | - | - | - | 3.3 |  |
|  | Pfizer, Moderna, or J&J | - | - | - | - | - | - | Epsilon | - | - | - | - | - | 2.1 |  |
|  | Pfizer, Moderna, or J&J | - | - | - | - | - | - | Iota | - | - | - | - | - | 2.0 |  |
| Peralta- Santos et al. ^54^ | Pfizer, Moderna, AstraZeneca, or J&J | - | - | - | - | - | 0.2 | Delta | - | - | - | - | - | 1.39 |  |
| Sharma et al. ^55^ | Pfizer, AstraZeneca, Covishield, Covaxin | - | - | - | - | - | 1.03 | - | - | - | - | - | - | - |  |
| Espenhain et al. ^57^ | Pfizer, Moderna, AztraZeneca, or J&J | - | - | - | - | - | 1.2 | Delta | - | - | - | - | - | 1.5 |  |
| Auvigne et al. ^59^ | Pfizer, Moderna, AztraZeneca, or J&J | - | - | - | - | - | 0.12 | Delta | - | - | - | - | - | 0.89 |  |
| Christensen et al. ^60^ | Pfizer, Moderna, or J&J | - | - | - | - | - | 19.8 | Alpha | - | - | - | - | - | 54.6 | p<0.0001 (Alpha & Omicron) |
|  | Pfizer, Moderna, or J&J | - | - | - | - | - | - | Delta | - | - | - | - | - | 43.1 | p<0.0001 (Delta & Omicron) |
| Houhamdi et al. ^61^ | NR | - | - | - | - | - | 1.9 | Delta | - | - | - | - | - | 11.9 | p<0.0001 (Delta & Omicron) |
| Ulloa et al. ^65^ | NR | - | - | - | - | - | 0.24 | Delta | - | - | - | - | - | 2.28 |  |
| CDC team ^66^ | NR | - | - | - | - | - | 2 | - | - | - | - | - | - | - |  |
| Klein et al. ^67^ | Pfizer | - | - | - | - | - | 2.75 | Delta | - | - | - | - | - | 6.13 |  |

Abbreviations: NR: Not reported.

*Case series

** No previously infected patients
