# Supplementary material for: Severity of the Omicron SARS‐CoV‐2 variant compared with the previous lineages: A systematic review
Source: J Cell Mol Med. 2023 May 18;27(11):1443–64. doi: 10.1111/jcmm.17747 (PMC10243162; doi:10.1111/jcmm.17747)
Supplement: Supplementary file 5 — TABLE S5 ICU admissions reported according to the vaccination status in cases infected with Omicron or other variants. [file JCMM-27-1443-s002.docx]

**Supplementary Table 5: ICU admissions reported according to the vaccination status in cases infected with Omicron or other variants**

| Study | Vaccine type | Omicron (no of doses) (%) | | | | | | Other variants (no of doses) (%) | | | | | | | P values or CI  Risk factors |
| --- | --- | --- | --- | --- | --- | --- | --- | --- | --- | --- | --- | --- | --- | --- | --- |
|  |  | Unvaccinated | Partially vaccinated | Fully vaccinated | Boosted | Dose NR | VS NR | Variant | Unvaccinated | Partially vaccinated | Fully vaccinated | Boosted | Dose NR | VS NR |  |
| Fall et al. ^5^ | Pfizer, Moderna, or J&J | 0.4 | - | 0.4 | 0.7 | - | - | Delta | 4.6 | - | 2.1 | 2.4 | - | - | p<0.00001 (Omicron & Delta)  p=0.5 (Omicron boosted and unvaccinated)  p=0.6 (Delta boosted and unvaccinated)  p=0.6 (Delta and Omicron boosted) |
| Goga et al. ^6^ | J&J | - | - | - | - | 0.11  (1-2 doses) | - | Delta | - | - | - | - | 0.37  (1-2 doses) | - | p<0.001 (Beta, Delta, and Omicron vaccinated) |
| Abu-Raddad et al. ^7^ ** | Pfizer | - | - | 0 | 0 | - | - | - | - | - | - | - | - | - |  |
|  | Moderna | - | - | 0 | 0 | - | - | - | - | - | - | - | - | - |  |
| Birollter et al. ^8^ | mRNA, inactive, or mixed | 3.8 | - | - | - | 0  (2+ doses) | - | - | - | - | - | - | - | - | p=0.285 (vaccinated vs unvaccinated)  Pregnant women |
| Lauring et al. ^10^ | Pfizer, Moderna, or mixed | 32.84 | - | - | - | 22.53  (2-3 doses) | - | Alpha | 37.39 | - | 20.69 | - | - | - | p=0.006 (Omicron vaccinated vs unvaccinated)  p<0.001 (Alpha vaccinated vs unvaccinated)  Hospitalized |
|  | Pfizer, Moderna, or mixed | - | - | - | - | - | - | Delta | 47.49 | - | - | - | 30.72  (2-3 doses) | - | p<0.001 (Delta vaccinated vs unvaccinated)  Hospitalized |
| Veneti et al. ^12^ | NR | - | - | - | - | - | 0.02 | Delta | - | - | - | - | - | 0.26 |  |
| Vieillard Baron et al. ^13^ | Pfizer, Moderna, or Astra Zeneca | - | - | - | - | - | 67.2 | Delta | - | - | - | - | - | 94.8 | p<0.001 (Omicron & Delta)  Hospitalized |
| Wang et al. ^14^ ** | NR | 0.14 | - | - | - | - | - | Delta | 0.43 | - | - | - | - | - | Pediatric |
| Marks et al. ^15^ | NR | 19.1 | - | 16.6 | - | - | - | Delta | 31.6 | - | 15.1 | - | - | - | p=0.009 (Vaccinated vs Unvaccinated)  Hospitalized adolescents and children |
| Iuliano et al. ^42^ | NR | - | - | - | - | - | 13.0 | Delta | - | - | - | - | - | 17.5 | p<0.05 (Omicron and Delta) Hospitalized |
|  | NR | - | - | - | - | - | - | Winter- Period | - | - | - | - | - | 18.2 | p<0.05 (Omicron and Winter- period) Hospitalized |
| Maisa et al. ^16^ | NR | - | - | - | - | 0  (0-3 doses) | - | - | - | - | - | - | - | - |  |
| Modes et al. ^20^ | mRNA vaccine | 19.0 | - | - | - | 15.3  (2-3 doses) | - | Delta | 22.8 | - | - | - | 23.5  (2-3 doses) | - | p=0.10 (Vaccinated Omicron and Delta)  p=0.27 (Unvaccinated Omicron and Delta)  p=0.01 (Omicron and Delta)  Hospitalized |
| Ludvingsson et al. ^22^ * ** | Pfizer | 50 | 0 | - | - | - | - | - | - | - | - | - | - | - | Children with convulsions |
| Dinh et al. ^35^ | NR | - | - | - | - | - | 0.03 | Delta | - | - | - | - | - | 0.07 |  |
|  | NR | - | - | - | - | - | - | Alpha | - | - | - | - | - | 0.10 | p<0.0001 (Alpha & Omicron) |
| Abdullah et al. ^36^ | NR | - | - | - | - | - | 1.0 | Ancestral, Beta, Delta | - | - | - | - | - | 4.3 | p=0.0007 (Delta & Omicron)  Hospitalized |
| Wang et al. ^38^ ** | Pfizer, Moderna, or J&J | - | - | - | - | - | 0.26 | Delta | - | - | - | - | - | 0.78 |  |
| Wang et al. ^39^ ** | NR | - | - | - | - | - | 0.47 | Delta | - | - | - | - | - | 1.0 |  |
| Maslo et al. ^40^ | Pfizer or J&J | - | - | - | - | - | 7.66 | Ancestral | - | - | - | - | - | 28.49 |  |
|  | NR | - | - | - | - | - | - | Beta | - | - | - | - | - | 25.30 |  |
|  | NR | - | - | - | - | - | - | Delta | - | - | - | - | - | 20.78 | p<0.001 (Delta & Omicron) |
| Jassat et al. ^41^ | NR | - | - | - | - | - | 0.46 | D614G | - | - | - | - | - | 2.01 | p<0.001 (D614G & Omicron) |
|  | NR | - | - | - | - | - | - | Beta | - | - | - | - | - | 1.60 | p<0.001 (Beta & Omicron) |
|  | NR | - | - | - | - | - | - | Delta | - | - | - | - | - | 1.44 | p<0.001 (Delta & Omicron) |
| Niemann et al. ^44^ | NR | - | - | - | - | - | 3.1 (Period 3) | (Period 1) | - | - | - | - | - | 11.9 | CLL |
|  | NR | - | - | - | - | - | 0.0 (Period 4) | (Period 2) | - | - | - | - | - | 12.5 | CLL |
| Vallejo et al. ^45^ * ** | NR | - | - | - | - | - | 100 | - | - | - | - | - | - | - | Pediatric |
| AraujodaSilva et al. ^48^ | NR | - | - | - | - | - | 51.6 | Pre-Omicron | - | - | - | - | - | 53.3 | p=0.894 (pre-Omicron & Omicron)  Hospitalized children |
| Lewnard et al. ^51^ | Pfizer, Moderna, or J&J | - | - | - | - | - | 0.03 | Delta | - | - | - | - | - | 0.12 |  |
|  | Pfizer, Moderna, or J&J | - | - | - | - | - | 0.05 | - | - | - | - | - | - | - |  |
|  | Pfizer, Moderna, or J&J | - | - | - | - | - | 0.05 | - | - | - | - | - | - | - |  |
| Peralta- Santos et al. ^54^ | Pfizer, Moderna, AstraZeneca, or J&J | - | - | - | - | - | 0 | Delta | - | - | - | - | - | 0.18 |  |
| Espenhain et al. ^57^ | Pfizer, Moderna, AztraZeneca, or J&J | - | - | - | - | - | 0.13 | Delta | - | - | - | - | - | 0.11 |  |
| Houhamdi et al. ^61^ | NR | - | - | - | - | - | 0.1 | Delta | - | - | - | - | - | 3.1 | p<0.0001 (Delta & Omicron) |
| Davies et al. ^62^ | Pfizer or J&J | - | - | - | - | - | 6.3 | Delta | - | - | - | - | - | 10.4 |  |
|  | Pfizer or J&J | - | - | - | - | - | 0.9 | Delta | - | - | - | - | - | 4.3 |  |
| Ulloa et al. ^65^ | NR | - | - | - | - | - | 0.02 | Delta | - | - | - | - | - | 0.47 |  |

Abbreviations: NR: Not reported.

*Case series

** No previously infected patients
