# Supplementary material for: Severity of the Omicron SARS‐CoV‐2 variant compared with the previous lineages: A systematic review
Source: J Cell Mol Med. 2023 May 18;27(11):1443–64. doi: 10.1111/jcmm.17747 (PMC10243162; doi:10.1111/jcmm.17747)
Supplement: Supplementary file 6 — TABLE S6 Cases in need of oxygenation/ventilation reported according to the vaccination status in cases infected with Omicron or other variants. [file JCMM-27-1443-s006.docx]

**Supplementary Table 6: Cases in need of oxygenation/ventilation reported according to the vaccination status in cases infected with Omicron or other variants**

| Study | Vaccine type | Type of treatment | Omicron (no of doses) (%) | | | | | | Other variants (no of doses) (%) | | | | | | | P values or CI  Risk factors |
| --- | --- | --- | --- | --- | --- | --- | --- | --- | --- | --- | --- | --- | --- | --- | --- | --- |
|  |  |  | Unvaccinated | Partially vaccinated | Fully vaccinated | Boosted | Dose NR | VS NR | Variant | Unvaccinated | Partially vaccinated | Fully vaccinated | Boosted | Dose NR | VS NR |  |
| Goga et al. ^6^ | J&J | Ventilation | - | - | - | - | 0.05  (1-2 doses) | - | Delta | - | - | - | - | 0.40  (1-2 doses) | - | p<0.001 (Beta, Delta, and Omicron vaccinated)  Hospitalized |
|  | J&J | Ventilation | - | - | - | - | - | - | Beta | - | - | - | - | 0.99  (1-2 doses) | - |  |
|  | J&J | Oxygenation | - | - | - | - | 0.52 | - | Delta | - | - | - | - | 2.26  (1-2 doses) | - | p<0.001 (Beta, Delta, and Omicron vaccinated)  Hospitalized |
|  | J&J | Oxygenation | - | - | - | - | - | - | Beta | - | - | - | - | 5.25  (1-2 doses) | - |  |
| Birollter et al. ^8^ | mRNA, active, or mixture | Oxygenation | 9.6 | - | - | - | 0  (2+ doses) | - | - | - | - | - | - | - | - | p=0.015 (vaccinated vs unvaccinated)  Pregnant women |
|  | mRNA, active, or mixture | Nasal oxygen support | 3.8 | - | - | - | 0  (2+ doses) | - | - | - | - | - | - | - | - | p=0.285 (vaccinated vs unvaccinated)  Pregnant women |
|  | mRNA, active, or mixture | NIV | 3.8 | - | - | - | 0  (2+ doses) | - | - | - | - | - | - | - | - | p=0.285  (vaccinated vs unvaccinated)  Pregnant women |
|  | mRNA, active, or mixture | IMV | 1.9 | - | - | - | 0  (2+ doses) | - | - | - | - | - | - | - | - | p=0.812 (vaccinated vs unvaccinated)  Pregnant women |
|  | mRNA, active, or mixture | ECMO | 0 | - | - | - | 0  (2+ doses) | - | - | - | - | - | - | - | - | Pregnant women |
| Lauring et al. ^10^ | Pfizer, Moderna, or mixed | IMV | 18.0 | - | - | - | 11.9  (2-3 doses) | - | Alpha | 21.3 | - | 6.0 | - | - | - | p=0.0043 (Omicron vaccinated vs unvaccinated)  p<0.001 (Alpha vaccinated vs unvaccinated)  Hospitalized |
|  | Pfizer, Moderna, or mixed | IMV | - | - | - | - | - | - | Delta | 24.8 | - | - | - | 14.5  (2-3 doses) | - | p=0<0.001 (Delta vaccinated vs unvaccinated)  Hospitalized |
|  | Pfizer, Moderna, or mixed | NIV | 15.8 | - | - | - | 13.3  (2-3 doses) | - | Alpha | 17.7 | - | 12.9 | - | - | - | p=0.40 (Omicron vaccinated vs unvaccinated)  p=0.20 (Alpha vaccinated vs unvaccinated)  Hospitalized |
|  | Pfizer, Moderna, or mixed | NIV | - | - | - | - | - | - | Delta | 17.1 | - | - | - | 14.4  (2-3 doses) | - | p=0.046 (Delta vaccinated vs unvaccinated)  Hospitalized |
|  | Pfizer, Moderna, or mixed | HFOT | 30.9 | - | - | - | 20.1  (2-3 doses) | - | Alpha | 34.3 | - | 12.9 | - | - | - | p=0.003 (Omicron vaccinated vs unvaccinated)  p<0.001 (Alpha vaccinated vs unvaccinated)  Hospitalized |
|  | Pfizer, Moderna, or mixed | HFOT | - | - | - | - | - | - | Delta | 41.9 | - | - | - | 27.7  (2-3 doses) | - | P<0.001 (Delta vaccinated vs unvaccinated)  Hospitalized |
| Vieillard- Baron et al. ^12^ | Pfizer, Moderna, or AstraZeneca | IMV | - | - | - | - | - | 41.0 | Delta | - | - | - | - | - | 51.0 | p=0.02 (Delta & Omicron)  Hospitalized |
| Wang et al. ^14^ ** | NR | MV | 0.33 | - | - | - | - | - | Delta | 1.15 | - | - | - | - | - | Pediatric |
| Marks et al. ^15^ | NR | IMV | 1.6 | - | 5.5 | - | - | - | Delta | 7.1 | - | 9.4 | - | - | - | p=0.54 (Vaccinated & Unvaccinated)  Hospitalized adolescents and children |
| Modes et al. ^20^ | mRNA vaccine | IMV | 10.8 | - | - | - | 8.6  (2-3 doses) | - | Delta | 15.4 | - | - | - | 9.4  (2-3 doses) | - | p=0.82 (Vaccinated Omicron and Delta)  p=0.11 (Unvaccinated Omicron and Delta)  p=0.03 (Omicron and Delta)  Hospitalized |
| Cloete et al. ^26^ | NR | Oxygen therapy | 20 | - | - | - | - | - | - | - | - | - | - | - | - | Pediatric children |
|  | NR | IMV | 5 | - | - | - | - | - | - | - | - | - | - | - | - | Pediatric children |
|  | NR | HFOT | 1 | - | - | - | - | - | - | - | - | - | - | - | - | Pediatric children |
|  | NR | Nasal prong oxygen | 14 | - | - | - | - | - | - | - | - | - | - | - | - | Pediatric children |
| Abdullah et al. ^36^ | NR | Oxygenation | - | - | - | - | - | 31.63 | - | - | - | - | - | - | - | Hospitalized |
|  | NR | IMV | - | - | - | - | - | 4.08 | - | - | - | - | - | - | - | Hospitalized |
|  | NR | NIV | - | - | - | - | - | 4.08 | - | - | - | - | - | - | - | Hospitalized |
| Wang et al. ^38^ ** | NR | MV | - | - | - | - | - | 0.07 | Delta | - | - | - | - | - | 0.43 |  |
| Wang et al. ^39^ ** | NR | MV | - | - | - | - | - | 0.08 | Delta | - | - | - | - | - | 0.3 |  |
| Maslo et al. ^40^ | Pfizer of J&J | Oxygen therapy | - | - | - | - | - | 7.27 | Ancestral | - | - | - | - | - | 54.68 |  |
|  | Pfizer or J&J | Oxygen therapy | - | - | - | - | - | - | Beta | - | - | - | - | - | 56.65 |  |
|  | Pfizer or J&J | Oxygen therapy | - | - | - | - | - | - | Delta | - | - | - | - | - | 51.40 | p<0.001 (Delta & Omicron) |
|  | Pfizer or J&J | MV | - | - | - | - | - | 0.68 | Ancestral | - | - | - | - | - | 11.12 |  |
|  | Pfizer or J&J | MV | - | - | - | - | - | - | Beta | - | - | - | - | - | 5.59 |  |
|  | Pfizer or J&J | MV | - | - | - | - | - | - | Delta | - | - | - | - | - | 8.64 | p<0.001 (Delta & Omicron) |
| Jassat et al. ^41^ | Pfizer or J&J | Supplemental oxygen | - | - | - | - | - | 1.68 | D614G | - | - | - | - | - | 4.68 | p<0.001 (D614G & Omicron) |
|  | Pfizer or J&J | Supplemental oxygen | - | - | - | - | - | - | Beta | - | - | - | - | - | 5.95 | p<0.001 (Beta & Omicron) |
|  | Pfizer or J&J | Supplemental oxygen | - | - | - | - | - | - | Delta | - | - | - | - | - | 4.69 | p<0.001 (Delta & Omicron) |
| Iuliano et al. ^42^ | NR | IMV | - | - | - | - | - | 3.5 | Delta | - | - | - | - | - | 6.6 | p<0.001 (Omicron and Delta)  Hospitalized |
|  | NR | IMV | - | - | - | - | - | - | Winter- Period | - | - | - | - | - | 7.5 | p<0.001 (Omicron and Winter-period)  Hospitalized |
| Martin et al. ^43^ | NR | MV | - | - | - | - | - | <11 | Pre- Omicron | - | - | - | - | - | 34.0 | Hospitalized pediatric with UAI |
| Lewnard et al. ^51^ | Pfizer, Moderna, or J&J | MV | - | - | - | - | - | 0.1 | Delta | - | - | - | - | - | 0.1 |  |
|  | Pfizer, Moderna, or J&J | MV | - | - | - | - | - | 0.05 | - | - | - | - | - | - | - |  |
|  | Pfizer, Moderna, or J&J | MV | - | - | - | - | - | 0.01 | - | - | - | - | - | - | - |  |
| Sharma et al. ^55^ | Pfizer, AstraZeneca, Covishield, Covaxin | Oxygenation | - | - | - | - | - | 0.7 |  |  |  |  |  |  |  |  |
| Christensen et al. ^60^ | Pfizer, Moderna, or J&J | IMV | - | - | - | - | - | 5.5 | Alpha | - | - | - | - | - | 8.4 | p<0.0001 (Alpha & Omicron) |
|  | Pfizer, Moderna, or J&J | IMV | - | - | - | - | - | - | Delta | - | - | - | - | - | 10.7 | p<0.0001 (Delta & Omicron) |
|  | Pfizer, Moderna, or J&J | NIV | - | - | - | - | - | 7.1 | Alpha | - | - | - | - | - | 9.5 | p<0.0001 (Alpha & Omicron) |
|  | Pfizer, Moderna, or J&J | NIV | - | - | - | - | - | - | Delta | - | - | - | - | - | 9.5 | p<0.0001 (Delta & Omicron) |
|  | Pfizer, Moderna, or J&J | ECMO | - | - | - | - | - | 0.1 | Alpha | - | - | - | - | - | 0.4 | p<0.0001 (Alpha & Omicron) |
|  | Pfizer, Moderna, or J&J | ECMO | - | - | - | - | - | - | Delta | - | - | - | - | - | 0.3 | p<0.0001 (Delta & Omicron) |
|  | Pfizer, Moderna, or J&J | HFOT | - | - | - | - | - | 8.1 | Alpha | - | - | - | - | - | 21.2 | p<0.0001 (Alpha & Omicron) |
|  | Pfizer, Moderna, or J&J | HFOT | - | - | - | - | - | - | Delta | - | - | - | - | - | 26.5 | p<0.0001 (Delta & Omicron) |
|  | Pfizer, Moderna, or J&J | LFOT | - | - | - | - | - | 35.5 | Alpha | - | - | - | - | - | 42.0 | p<0.0001 (Alpha & Omicron) |
|  | Pfizer, Moderna, or J&J | LFOT | - | - | - | - | - | - | Delta | - | - | - | - | - | 33.8 | p<0.0001 (Delta & Omicron) |

Abbreviations: ECMO: Extracorporeal membrane oxygenation, HFOT: High flow oxygen therapy, IMV: Invasive mechanical ventilation, LFOT: Low flow oxygen therapy, MV: Mechanical ventilation, NIV: Noninvasive ventilation, NR: Not reported.

*Case series

** No previously infected patients
