# Supplementary material for: Severity of the Omicron SARS‐CoV‐2 variant compared with the previous lineages: A systematic review
Source: J Cell Mol Med. 2023 May 18;27(11):1443–64. doi: 10.1111/jcmm.17747 (PMC10243162; doi:10.1111/jcmm.17747)
Supplement: Supplementary file 7 — TABLE S7 Cases of cardiovascular and haematological complications reported according vaccination status in cases infected with Omicron or other variants. [file JCMM-27-1443-s010.docx]

**Supplementary Table 7: Cases of cardiovascular and hematological complications reported according vaccination status in cases infected with Omicron or other variants**

| Study | Vaccine type | Type of event | Omicron (no of doses) (%) | | | | | | Other variants (no of doses) (%) | | | | | | | P values or CI  Risk factors |
| --- | --- | --- | --- | --- | --- | --- | --- | --- | --- | --- | --- | --- | --- | --- | --- | --- |
|  |  |  | Unvaccinated | Partially vaccinated | Fully vaccinated | Boosted | Dose NR | VS NR | Variant | Unvaccinated | Partially vaccinated | Fully vaccinated | Boosted | Dose NR | VS NR |  |
| Lauring et al. ^10^ | Pfizer, Moderna, or mixed | MI | 1.84 | - | - | - | 1.71  (2-3 doses) | - | Alpha | 2.01 | - | 0.86 | - | - | - | p=0.91 (Omicron vaccinated vs unvaccinated)  p=0.39 (Alpha vaccinated vs unvaccinated)  Hospitalized |
|  | Pfizer, Moderna, or mixed | MI | - | - | - | - | - | - | Delta | 2.12 | - | - | - | 2.78 | - | p=0.23 (Delta vaccinated vs unvaccinated)  Hospitalized |
|  | Pfizer, Moderna, or mixed | Stroke | 1.47 | - | - | - | 1.02 | - | Alpha | 1.91 | - | 0 | - | - | - | p=0.63 (Omicron vaccinated vs unvaccinated)  p=0.13 (Alpha vaccinated vs unvaccinated)  Hospitalized |
|  | Pfizer, Moderna, or mixed | Stroke | - | - | - | - | - | - | Delta | 1.60 | - | - | - | 0.86 | - | p=0.08 (Delta vaccinated vs unvaccinated)  Hospitalized |
|  | Pfizer, Moderna, or mixed | VTEE | 8.09 | - | - | - | 5.12  (2-3 doses) | - | Alpha | 5.83 | - | 6.03 | - | - | - | p=0.15 (Omicron vaccinated vs unvaccinated)  p=0.93 (Alpha vaccinated vs unvaccinated)  Hospitalized |
|  | Pfizer, Moderna, or mixed | VTEE | - | - | - | - | - | - | Delta | 9.11 | - | - | - | 4.40  (2-3 doses) | - | p<0.001 (Delta vaccinated vs unvaccinated)  Hospitalized |
| Vallejo et al. ^45^ * ** | NR | CVST | - | - | - | - | - | 100 | - | - | - | - | - | - | - | Pediatric |

Abbreviations: CVST: Cerebral venous sinus thrombosis, MI: Myocardial infarction, NR: Not reported, VTEE: Venous thromboembolic events.

*Case series

** No previously infected patients
