# Supplementary material for: Severity of the Omicron SARS‐CoV‐2 variant compared with the previous lineages: A systematic review
Source: J Cell Mol Med. 2023 May 18;27(11):1443–64. doi: 10.1111/jcmm.17747 (PMC10243162; doi:10.1111/jcmm.17747)
Supplement: Supplementary file 8 — TABLE S8 Death cases reported according to the vaccination status in cases infected with Omicron or other variants. [file JCMM-27-1443-s003.docx]

**Supplementary Table 8: Death cases reported according to the vaccination status in cases infected with Omicron or other variants**

| Study | Vaccine type | Omicron (no of doses) (%) | | | | | | Other variants (no of doses) (%) | | | | | | | P values or CI  Risk factors |
| --- | --- | --- | --- | --- | --- | --- | --- | --- | --- | --- | --- | --- | --- | --- | --- |
|  |  | Unvaccinated | Partially vaccinated | Fully vaccinated | Boosted | Dose NR | VS NR | Variant | Unvaccinated | Partially vaccinated | Fully vaccinated | Boosted | Dose NR | VS NR |  |
| Fall et al. ^5^ | Pfizer, Moderna, or J&J | 0.2 | - | 0 | 0 | - | - | Delta | 1.2 | - | 0.7 | 2.4 | - | - | p<0.00001 (Delta & Omicron)  p=1.0 (Omicron boosted and unvaccinated)  p=0.3 (Delta boosted and unvaccinated)  p=0.1 (Delta and Omicron boosted) |
| Abu-Raddad et al. ^7^ ** | Pfizer | - | - | 0 | 0 | - | - | - | - | - | - | - | - | - |  |
|  | Moderna | - | - | 0 | 0 | - | - | - | - | - | - | - | - | - |  |
| Birollter et al. ^8^ | mRNA, inactive, or mixture | - | - | - | - | 0  (2-3 doses) | - | - | - | - | - | - | - | - |  |
| Lauring et al. ^10^ | Pfizer, Moderna, or mixed | 9.19 | - | - | - | 5.12  (2-3 doses) | - | Alpha | 8.05 | - | 4.31 | - | - | - | p=0.15 (Alpha vaccinated vs unvaccinated)  p=0.059 (Omicron vaccinated vs unvaccinated)  Hospitalized |
|  | Pfizer, Moderna, or mixed | - | - | - | - | - | - | Delta | 11.78 | - | - | - | 13.21  (2-3 doses) | - | p=0.23 (Delta vaccinated vs unvaccinated)  Hospitalized |
| Lee et al. ^11^ | Pfizer, Moderna, or J&J | 3.57 | 11.1 | - | - | 5.85  (2-3 doses) | - | - | - | - | - | - | - | - | Cancer |
| Veneti et al. ^12^ | NR | - | - | - | - | - | 0.03 | Delta | - | - | - | - | - | 0.18 |  |
| Viellard- Baron et al. ^13^ | Pfizer, Moderna, or AstraZeneca | - | - | - | - | - | 20.0 | Delta | - | - | - | - | - | 27.9 | p=0.08 (Delta & Omicron)  Hospitalized |
| Marks et al. ^15^ | NR | 0 | - | 0 | - | - | - | Delta | 0.9 | - | 3.8 | - | - | - | p=0.10 (Vaccinated vs Unvaccinated)  Hospitalized adolescents and children |
| Maisa et al. ^16^ | NR | - | - | - | - | - | 0  (0-3 doses) | - | - | - | - | - | - | - |  |
| Modes et al. ^20^ | mRNA vaccine | 4.9 | - | - | - | 3.4  (2-3 doses) | - | Delta | 7.9 | - | - | - | 4.9  (2-3 doses) | - | p=0.02 (Vaccinated Omicron and Delta)  p=0.21 (Unvaccinated Omicron and Delta)  p=0.01 (Omicron and Delta)  Hospitalized |
| Cloete et al. ^26^ | NR | 3 | - | - | - | - | - | - | - | - | - | - | - | - | Pediatric patients |
| Goussard et al. ^27^ * ** | NR | 100 | - | - | - | - | - | - | - | - | - | - | - | - | Prematurity |
| Ward et al. ^31^ | Pfizer, Moderna, or AstraZeneca | - | - | - | - | 0.02  (1-3 doses) | - | Delta | - | - | - | - | 0.09 |  |  |
| Sami et al. ^32^ | Pfizer, Moderna, or J&J | - | - | - | - | 0  (1-3 doses) | - | - | - | - | - | - | - | - |  |
|  | Pfizer, Moderna, or J&J | - | - | - | - | 0  (1-3 doses) | - | - | - | - | - | - | - | - |  |
| Patalon et al. ^33^ | Pfizer | - | - | 0 | 0.01 | - | - | - | - | - | - | - | - | - |  |
| Abdullah et al. ^36^ | NR | - | - | - | - | - | 4.5 | Ancestral, Beta, Delta | - | - | - | - | - | 21.3 | p<0.00001 (Delta & Omicron)  Hospitalized |
| Maslo et al. ^40^ | Pfizer or J&J | - | - | - | - | - | 1.15 | Ancestral | - | - | - | - | - | 13.42 |  |
|  | Pfizer or J&J | - | - | - | - | - | - | Beta | - | - | - | - | - | 17.06 |  |
|  | Pfizer or J&J | - | - | - | - | - | - | Delta | - | - | - | - | - | 20.25 | p<0.001 (Delta & Omicron) |
| Jassat et al. ^41^ | Pfizer or J&J | - | - | - | - | - | 0.78 | D614G | - | - | - | - | - | 2.73 | p<0.001 (D614G & Omicron) |
|  | Pfizer or J&J | - | - | - | - | - | - | Beta | - | - | - | - | - | 3.58 | p<0.001 (Beta & Omicron) |
|  | Pfizer or J&J | - | - | - | - | - | - | Delta | - | - | - | - | - | 2.60 | p<0.001 (Delta & Omicron) |
| Iuliano et al. ^42^ | NR | - | - | - | - | - | 7.1 | Delta | - | - | - | - | - | 12.3 | Hospitalized |
|  | NR | - | - | - | - | - | - | Winter- Period | - | - | - | - | - | 12.9 | Hospitalized |
| Niemann et al. ^44^ | NR | - | - | - | - | - | 9.4 (Period 3) | (Period 1) | - | - | - | - | - | 16.9 |  |
|  | NR | - | - | - | - | - | 22.7 (Period 4) | (Period 2) | - | - | - | - | - | 17.5 |  |
| Krutikov et al. ^47^ | AstraZeneca, Pfizer, or NR | - | - | - | - | - | 5.3 | Delta | - | - | - | - | - | 12.8 | p<0.0001 (Omicron & Delta) |
| AraujodaSilva et al. ^48^ | NR | - | - | - | - | - | 1.7 | Pre-Omicron | - | - | - | - | - | 2.5 | p=0.894 (pre-Omicron & Omicron)  Hospitalized children |
| Madhi et al. ^50^ | NR | - | - | - | - | - | 0.49 | Wild- Type | - | - | - | - | - | 2.78 |  |
|  | NR | - | - | - | - | - | - | Beta | - | - | - | - | - | 3.88 |  |
|  | NR | - | - | - | - | - | - | Delta | - | - | - | - | - | 2.79 |  |
| Lewnard et al. ^51^ | Pfizer, Moderna, or J&J | - | - | - | - | - | 0.01 | Delta | - | - | - | - | - | 0.08 |  |
|  | Pfizer, Moderna, or J&J | - | - | - | - | - | 0.1 | - | - | - | - | - | - | - |  |
|  | Pfizer, Moderna, or J&J | - | - | - | - | - | 0.13 | - | - | - | - | - | - | - |  |
| Lee et al. ^53^ | Pfizer, Moderna, J&J, AstraZeneca | - | - | - | - | - | 0 | - | - | - | - | - | - | - |  |
| Peralta- Santos et al. ^54^ | Pfizer, Moderna, AstraZeneca, or J&J | - | - | - | - | - | 0 | Delta | - | - | - | - | - | 0.3 |  |
| Sharma et al. ^55^ | Pfizer, AstraZeneca, Covishield, Covaxin | - | - | - | - | - | 0.3 |  |  |  |  |  |  |  |  |
| Espenhain et al. ^57^ | Pfizer, Moderna, AztraZeneca, or J&J | - | - | - | - | - | 0 | Delta | - | - | - | - | - | 0.07 |  |
| Christensen et al. ^60^ | Pfizer, Moderna, or J&J | - | - | - | - | - | 0.9 | Alpha | - | - | - | - | - | 5.4 | p<0.0001 (Alpha & Omicron) |
|  | Pfizer, Moderna, or J&J | - | - | - | - | - | - | Delta | - | - | - | - | - | 5.3 | p<0.0001 (Delta & Omicron) |
| Houhamdi et al. ^61^ | NR | - | - | - | - | - | 0.1 | Delta | - | - | - | - | - | 1.3 | p<0.0001 (Delta & Omicron) |
| Davies et al. ^62^ | Pfizer or J&J | - | - | - | - | - | 0.8 | Delta | - | - | - | - | - | 5.7 |  |
|  | NR | - | - | - | - | - | - | NR | 1.8 | - | - | - | - | - |  |
|  | NR | - | - | - | - | - | - | NR | 3.4 | - | - | - | - | - |  |
| Ulloa et al. ^65^ | NR | - | - | - | - | - | 0.01 | Delta | - | - | - | - | - | 0.54 |  |
| CDC Team ^66^ | NR | - | - | - | - | - | 0 | - | - | - | - | - | - | - |  |

Abbreviations: NR: Not reported.

*Case series

** No previously infected patients
