# Supplementary material for: Severity of the Omicron SARS‐CoV‐2 variant compared with the previous lineages: A systematic review
Source: J Cell Mol Med. 2023 May 18;27(11):1443–64. doi: 10.1111/jcmm.17747 (PMC10243162; doi:10.1111/jcmm.17747)
Supplement: Supplementary file 9 — TABLE S9 Cases with other complications reported according to number of doses in cases with Omicron and other variants. [file JCMM-27-1443-s008.docx]

**S9: Cases with other complications reported according to number of doses in cases with Omicron and other variants**

| **Study** | **Vaccine type** | **Type of event** | **Omicron (no of doses) (%)** | | | | | | **Other variants (no of doses) (%)** | | | | | | | **P values or CI**  **Risk factors** |
| --- | --- | --- | --- | --- | --- | --- | --- | --- | --- | --- | --- | --- | --- | --- | --- | --- |
|  |  |  | 0 | 1 | 2 | 3 | Dose NR | VS NR | Variant | 0 | 1 | 2 | 3 | Dose NR | VS NR |  |
| Birollter et al. ^8^ | mRNA, inactive, or mixed | Moderate/ serious symptoms | 9.6 | - | - | - | 0  (2+ doses) | - | - | - | - | - | - | - | - | Pregnant women |
| Sharma et al. ^55^ | Pfizer, AstraZeneca, Covishield, Covaxin | Moderate | - | - | - | - | - | 10.0 | - | - | - | - | - | - | - |  |
| Wolter et al. ^37^ | J&J or Pfizer | Severe | - | - | - | - | - | 33.5 (BA1)  30.5 (BA2) | - | - | - | - | - | - | - | Hospitalized |
| Wolter et al. ^56^ | J&J or Pfizer | Severe | - | - | - | - | 30.9 | - | Omicron & Delta (mixed set) | - | - | - | - | 44.4  (1+ doses) | - |  |
| Goga et al. ^6^ | J&J | High care | - | - | - | - | 0.21  (1-2 doses) | - | Delta | - | - | - | - | 0.25  (1-2 doses) | - |  |
| Robinson et al. ^21^ | Pfizer, Moderna, or J&J | Severe/ Death | 9.35 | - | 4.00 | - | - | - | Alpha | 30.84 | - | 22.22 | - | - | - | Hospitalized |
|  | Pfizer, Moderna, or J&J | Severe/ Death | - | - | - | - | - | - | Delta | 20.77 | - | 17.18 | - | - | - | Hospitalized |
|  | Pfizer, Moderna, or J&J | Severe/ Death | - | - | - | - | - | - | Ancestral | 1.98 | - | 0 | - | - | - | Hospitalized |
|  | Pfizer, Moderna, or J&J | Severe/Death | - | - | - | - | - | - | Other | 26.5 | - | 0 | - | - | - | Hospitalized |
| Davies et al. ^62^ | Pfizer or J&J | Severe admissions | - | - | - | - | - | 0.9 | Delta | - | - | - | - | - | 4.3 |  |
|  | Pfizer or J&J | Severe admissions | - | - | - | - | - | - | Beta | - | - | - | - | - | 3.4 |  |
| Jassat et al. ^41^ | Pfizer or J&J | Severe symptoms | - | - | - | - | - | 2.45 | D614G | - | - | - | - | - | 6.65 | p<0.001 (D614G & Omicron) |
|  | Pfizer or J&J | Severe symptoms | - | - | - | - | - | - | Beta | - | - | - | - | - | 7.88 | p<0.001 (Beta & Omicron) |
|  | Pfizer or J&J | Severe symptoms | - | - | - | - | - | - | Delta | - | - | - | - | - | 6.20 | p<0.001 (Delta & Omicron) |
| Martin et al. ^43^ | NR | Severe symptoms | - | - | - | - | - | <11 | Pre- Omicron | - | - | - | - | - | 38.8 | p<0.001 (Omicron and pre-Omicron)  Hospitalized pediatric |
| Lee et al. ^53^ | Pfizer, Moderna, J&J, AstraZeneca | Severe symptoms | - | - | - | - | - | 0 | - | - | - | - | - | - | - |  |
| Sharma et al. ^55^ | Covishield, Covaxin, Pfizer, AstraZeneca | Severe symptoms | - | - | - | - | - | 0.7 | - | - | - | - | - | - | - |  |
| Auvigne et al. ^59^ | Pfizer, Moderna, AstraZeneca, or J&J | Severe hospital events | - | - | - | - | - | 0.12 | Delta | - | - | - | - | - | 0.89 | p<0.001 (Delta & Omicron) |
| Helmsdal et al. ^30^ | Pfizer | Anosmia | - | - | - | 19 | - | - | - | - | - | - | - | - | - |  |
|  | Pfizer | Ageusia | - | - | - | 24 | - | - | - | - | - | - | - | - | - |  |
| Brandal et al. ^46^ | Pfizer or Moderna | Hyposmia | - | - | - | - | - | 12.35 | - | - | - | - | - | - | - |  |
|  | Pfizer or Moderna | Hyposgeusia | - | - | - | - | - | 23.46 | - | - | - | - | - | - | - |  |
| Kim et al. ^49^ ** | Pfizer, Moderna, AstraZeneca, J&J | Hyposmia/ Hypogeusia | - | - | - | - | - | 2.5 | - | - | - | - | - | - | - |  |
| Lee et al. ^53^ | Pfizer, Moderna, J&J, AstraZeneca | Anosmia/ Ageusia | - | - | - | - | - | 1.3 | - | - | - | - | - | - | - |  |
| Boscolo- Rizzo et al. ^58^ | NR | Smell and taste impariments | - | - | - | - | - | 19.2 | - | - | - | - | - | - | - |  |
|  | NR | Smell impariment (only) | - | - | - | - | - | 5.3 | - | - | - | - | - | - | - |  |
|  | NR | Taste impairment (only) | - | - | - | - | - | 7.7 | - | - | - | - | - | - | - |  |
| Hajjo et al. ^63^ | NR | Hyposmia/ Hypogeusia | - | - | - | - | - | 1.2 | - | - | - | - | - | - | - |  |
| CDC Team ^66^ | Pfizer | Hyposmia/ Hypogeusia | - | - | - | - | - | 8 | - | - | - | - | - | - | - |  |
| Wang et al. ^14^ ** | NR | ED visits | 18.83 | - | - | - | - | - | Delta | 26.67 | - | - | - | - | - | Pediatric |
| Wang et al. ^38^ ** | Pfizer, Moderna, or J&J | ED visits | - | - | - | - | - | 4.55 | Delta | - | - | - | - | - | 15.22 |  |
| Wang et al. ^39^ ** | NR | ED visits | - | - | - | - | - | 10.19 | Delta | - | - | - | - | - | 14.63 |  |
| Tseng et al. ^24^ | Moderna | ED visits | - | 14.7 | 15.6 | 16.6 | - | - | Delta | - | 14.6 | 15.0 | 14.6 | - | - |  |
| Lee et al. ^11^ | Pfizer, Moderna, or J&J | ER/outpatient | - | - | - | - | - | 84.0  (0-3 doses) | - | - | - | - | - | - | - |  |
| Piersiala et al. ^23^ | NR | Odynophagia | 100 | 100 | 100 | 100 | - | - | - | - | - | - | - | - | - | Patients with odynophagia |
| Lauring et al. ^10^ | Pfizer, Moderna, or mixed | NRRT | 4.41 | - | - | - | 4.78  (2-3 doses) | - | Alpha | 4.56 | - | 5.17 | - | - | - | p=0.91 (Alpha vaccinated vs unvaccinated)  p=0.84 (Omicron vaccinated vs unvaccinated)  Hospitalized |
|  | Pfizer, Moderna, or mixed | NRRT | - | - | - | - | - | - | Delta | 5.80 | - | - | - | 4.69  (2-3 doses) | - | p=0.18 (Delta vaccinated vs unvaccinated)  Hospitalized |
|  | Pfizer, Moderna, or mixed | Vasopressors | 16.91 | - | - | - | 12.29  (2-3 doses) | - | Alpha | 20.23 | - | 4.31 | - | - | - | p<0.001 (Alpha vaccinated vs unvaccinated)  p<0.001 (Omicron vaccinated vs unvaccinated)  Hospitalized |
|  | Pfizer, Moderna, or mixed | Vasopressors | - | - | - | - | - | - | Delta | 23.59 | - | - | - | 14.83  (2-3 doses) | - | p=0.12 (Delta vaccinated vs unvaccinated)  Hospitalized |
| Viellard- Baron et al. ^13^ | Pfizer, Moderna, or AstraZeneca | Pneumonia | 80.7 | - | - | - | 62.1  (1-3 doses) | - | Delta | 97.8 | - | - | - | 87.5  (1-3 doses) | - | p<0.001 (Omicron vaccinated & unvaccinated)  p<0.01 (Delta vaccinated & unvaccinated)  p<0.001 (Delta & Omicron)  Hospitalized |
| Abdullah et al. ^36^ | NR | Pneumonia | - | - | - | - | - | 36.7 | - | - | - | - | - | - | - |  |
| Goussard et al. ^27^ * ** | NR | Apnea | 100 | - | - | - | - | - | - | - | - | - | - | - | - | Pediatric/ prematurity |
|  | NR | Respiratory failure | 100 | - | - | - | - | - | - | - | - | - | - | - | - | Pediatric/ prematurity |
| Dinh et al. ^35^ | NR | Respiratory symptoms | - | - | - | - | - | 49.8 | Alpha | - | - | - | - | - | 58.5 | p<0.001 (Omicron & Alpha) |
|  | NR | Respiratory symptoms | - | - | - | - | - | - | Delta | - | - | - | - | - | 62.4 |  |
|  | NR | Digestive symptoms | - | - | - | - | - | 45.1 | Alpha | - | - | - | - | - | 34.0 |  |
|  | NR | Digestive symptoms | - | - | - | - | - | - | Delta | - | - | - | - | - | 63.7 |  |
|  | NR | Systemic symptoms | - | - | - | - | - | 51.8 | Alpha | - | - | - | - | - | 58.4 |  |
|  | NR | Systemic symptoms | - | - | - | - | - | - | Delta | - | - | - | - | - | 64.1 |  |
| Martin et al. ^43^ | NR | Moderate symptoms | - | - | - | - | - | 96.6 | Pre- Omicron | - | - | - | - | - | 63.6 | Hospitalized pediatric with UAI |
| Kim et al. ^49^ ** | Pfizer, Moderna, AstraZeneca, J&J | Lung filtrations on CXR/CT | - | - | - | - | - | 15 | - | - | - | - | - | - | - |  |
| Vallejo et al. ^45^ * ** | NR | Sinusitis | - | - | - | - | - | 100 | - | - | - | - | - | - | - |  |
| Ebell et al. ^34^ | NR | Dyspnea | - | - | - | - | - | 4.18 | - | - | - | - | - | - | - |  |

Abbreviations: CXR/CT: Chest x-ray/CT, ED: Emergency department, NR: Not reported, NRRT: New renal replacement therapy.

*Case series

** No previously infected patients
